# Weight regain and mental health outcomes following behavioural weight management programmes: A systematic review with meta‐analyses

**DOI:** 10.1111/cob.12575

**Published:** 2023-01-09

**Authors:** Annika Theodoulou, Jamie Hartmann‐Boyce, Jordan Gorenberg, Jason L. Oke, Ailsa R. Butler, Anastasios Bastounis, Susan A. Jebb, Paul Aveyard

**Affiliations:** ^1^ Nuffield Department of Primary Care Health Sciences University of Oxford Oxford UK; ^2^ NIHR Oxford Biomedical Research Centre Oxford University Hospitals NHS Foundation Trust Oxford UK; ^3^ Division of Epidemiology and Public Health, School of Medicine University of Nottingham Nottingham UK

**Keywords:** mental health, meta‐analysis, obesity, systematic review, weight

## Abstract

Behavioural weight management programmes (BWMPs) lead to weight loss but subsequent weight regain may harm mental health outcomes. We searched for randomised trials of BWMPs in adults with overweight/obesity with follow‐up ≥12 months from baseline that measured weight change both at and after programme‐end. We included only studies reporting mental health at or after programme‐end. We meta‐analysed changes in various mental health outcomes using a random‐effects model by nature of the comparator group and by time since programme end. Subgroup analysis explored heterogeneity. We used mixed models and meta‐regression to analyse the association between change in weight and change in depression and/or anxiety over time, with higher scores indicating greater depression and/or anxiety. We included 47 studies. When comparing BWMPs (diet and/or exercise) to control, most estimates included the possibility of no difference, but pooled estimates for psychological wellbeing, self‐esteem and mental‐health composite scores at programme‐end, anxiety at 1–6 months, and depression at 7–12 months after programme‐end suggested improvements in intervention arms relative to control, with 95% CIs excluding no difference. Pooled estimates found no evidence that BWMPs harmed mental health at programme end or beyond. Mental health composite scores at programme‐end favoured diet and exercise interventions over diet alone, with 95% CIs excluding no difference. All other measures and timepoints included the possibility of no difference or could not be meta‐analysed due to high heterogeneity or a paucity of data. Mixed models and meta‐regression of the association between change in depression and/or anxiety scores over time, and change in weight, were inconclusive. Despite weight regain after BWMPs, our meta‐analyses found no evidence of mental health harm and some evidence that BWMPs may improve some dimensions of mental health at and after programme‐end.

**What is already known about this subject**

- Behavioural weight management programmes (BWMPs) help people living with overweight or obesity lose weight and improve physical health.^1^ However, weight regain is common, and there are concerns that this may worsen mental health.
- Evidence suggests improvements in depression, mental health‐related quality‐of‐life and self‐efficacy after a BWMP and at 12‐months from baseline compared to minimal intervention or usual care.^2^ However the longer‐term impact on mental health, and the impact of BWMP type and weight regain after BWMP end is unknown.

**What this study adds**

- This secondary analysis of a companion review identified 47 randomised trials of BWMPs in adults with overweight/obesity reporting mental health at programme‐end and ≥12 months from baseline.
- Despite weight regain, meta‐analyses found no evidence of mental health harm and some evidence that BWMPs may improve some dimensions of mental health at and after programme‐end. When comparing BWMP (diet and/or exercise) to control, most estimates included the possibility of no difference, but pooled estimates for psychological wellbeing, self‐esteem and mental‐health composite scores at programme‐end, anxiety at 1–6 months, and depression at 7–12 months after programme‐end suggested improvements in BWMP relative to control. Mental health composite scores at programme‐end favoured diet and exercise interventions over diet alone.
- Evidence on the association between change in depression and/or anxiety over time, and change in weight were inconclusive.

## 1 INTRODUCTION

Behavioural weight management programmes (BWMPs) lead to weight loss and at 1 year there is evidence of improved physical health.^1^ A recent systematic review investigated the impact of BWMPs on mental health related outcomes and reported improvements in depression, mental health‐related quality‐of‐life (QoL) and self‐efficacy at intervention end and at 12‐months compared to minimal intervention or usual care comparators.^2^ No difference in anxiety, overall QoL, self‐esteem or stress was found at intervention end.^2^ However, weight regain after programme end is common, and there are concerns that this may worsen mental health.^3^

Other evidence syntheses have investigated the impact of behavioural weight loss interventions on mental health but have been limited in scope based on programme type, population,^4^ or short term follow‐up.^2^, ^4^, ^5^, ^6^ We aim to take a more expansive approach, synthesising evidence on mental health outcomes following BWMPs assessed in randomised controlled trials (RCTs) with longer‐term follow‐up (≥12‐months from baseline). In addition, we set out to assess the extent to which changes in depression and/or anxiety following programme‐end are associated with weight change trajectories. Specifically, we aimed to address the following questions:

1. What is the effect of BWMPs compared with no/minimal interventions on mental health and psychological variables at programme‐end and after programme‐end?
2. Is any effect on mental health modified by the type of BWMP?
3. Is there an association between weight change and change in mental health, specifically depression and/or anxiety?

## 2 MATERIALS AND METHODS

This secondary analysis is based on a parent review investigating weight change and cardiometabolic outcomes,^1^, ^7^ and associated programme characteristics^3^ following BWMPs. The protocol for this analysis was pre‐registered on PROSPERO (CRD42020196101).^8^

### 2.1 Search strategy and eligibility criteria

Full details on the search strategy and eligibility criteria are available elsewhere.^1^, ^3^, ^7^, ^8^ In brief, we searched clinical trial registries, 11 electronic databases and the University of Aberdeen register of weight loss trials. Studies had to be RCTs of adults (≥18 years) with overweight or obesity. Interventions included any weight management programme which aimed to achieve weight loss through changes to diet and/or activity delivered in any setting. These included but were not limited to single or multi‐component behavioural counselling, self‐help programmes and/or diet replacement programmes.

Comparators included another BWMP, an intervention of lesser intensity, or no intervention. We excluded studies in pregnancy, interventions targeting multiple risk factors and interventions involving medications and/or surgery. Studies had to follow participants for ≥12 months from baseline, measure weight change both at programme‐end and after programme‐end, and for the present review, measure a mental health outcome at or after programme‐end. Where interventions varied in levels of support offered, we defined programme‐end as the point at which contact intensity markedly reduced.

### 2.2 Outcomes

Any measure of mental health and/or psychological variables, including overall composite scales and condition‐specific mental health scales, for example indexes of depression (e.g. Beck Depression Inventory) or self‐esteem (e.g. Rosenberg Self‐Esteem Scale). Weight change was analysed as an explanatory factor when considering influences on mental health.

### 2.3 Study selection, data extraction and quality assessment

Two reviewers independently screened studies for inclusion. Data extraction and risk of bias (RoB) assessment were conducted by one reviewer and checked by a second. We assessed study‐level RoB in: random sequence generation; allocation concealment; blinding of outcome assessment; attrition; other RoB using the Cochrane RoB tool (v1) for randomised trials. Any disagreements were resolved by discussion or referral to a third reviewer.

### 2.4 Data synthesis

Studies were grouped for analysis by the mental health outcome measured and the comparator group. As previously, no/minimal intervention comparator groups were labelled as ‘controls’, subdivided into groups 1–4 based on intensity^9^, ^10^:

1. No intervention at all or leaflet/s only
2. Discussion/advice/counselling in one‐off session +/−leaflet
3. Seeing someone more than once for discussion of something other than weight loss.
4. Seeing someone more than once for weight management, person untrained +/− leaflets

We pooled studies comparing BWMPs to any of the control groups, and sub‐grouped by control group intensity.^1^, ^2^, ^3^, ^4^ We pooled studies comparing diet and exercise programmes to diet only or exercise only programmes. Studies comparing diet and exercise programmes head‐to‐head or describing a unique intervention that did not fit our coding system were not pooled but presented in forest plots and reported narratively.

Data conversions were undertaken as necessary (e.g., converting standard error [SE] to standard deviations [SDs]) following Cochrane guidelines.^11^ Where the direction of the scale varied between studies, mean values were multiplied where necessary by −1 to ensure all scales pointed in the same direction for a specific mental health condition.

### 2.5 Statistical synthesis

We assessed the impact of BWMPs versus comparators on changes in mental health at and after programmes ceased using a random‐effects model. Similar outcomes were pooled by mental health condition, nature of comparison and at similar times after programme‐end (grouping outcomes that occurred in any 6‐month period until the longest follow‐up available). Analysis used Review Manager 5.4.1.^12^ We present mean differences (MDs) or standardised mean differences (SMDs) (when different scales were used to measure the same mental health condition) and 95% confidence intervals (CIs).

Statistical heterogeneity was measured using I^2^. Subgroup analysis was used to explore heterogeneity due to the nature of the control. Forest plots are ordered by mean weight change difference between intervention minus comparator/control groups, where possible, to visually explore the impact of weight difference on mental health outcomes. An I^2^ of >75% was used as an upper threshold for considering appropriateness of pooling estimates when heterogeneity could not be explained by weight change or subgrouping. Test for publication bias using funnel plots was only possible for one comparison due to insufficient data (≤10 studies).

We conducted post‐hoc analyses to model change in depression and/or anxiety over time and to test for associations between weight change and depression and/or anxiety outcomes following methods used in a previous paper.^1^ For studies reporting more than one of these outcomes, we preferentially analysed data of outcomes in order of ‘Depression and Anxiety’, ‘Depression’, or ‘Anxiety’.

We assessed whether weight regain was associated with change in mental health over time using three methods (in R 4.0.2) to assess whether the results were sensitive to the choice of synthesis method:

- Mixed model with a random intercept for each study, regressing outcomes at any time since follow‐up on time since programme‐end; unweighted by study precision.
- Meta‐regression against time since programme‐end, assuming linear increases in outcomes plotted as baseline and value at longest follow‐up. This weights studies by their variance (precision). We also used meta‐regression to examine if weight regain relative to control was associated with depression and/or anxiety.
- Time‐to‐event Kaplan‐Meir, evaluating the time at which half of the studies had an estimate for the difference between BWMP and control that reached zero.

Meta‐analysis results are presented using complete case data, where available. Missing data on the number of participants (*N*) were imputed using N at baseline or the next available timepoint. A baseline weight of 100 kg for each study arm was estimated to calculate weight change in rare instances where only a percentage weight change at follow‐up was reported. All imputations are reported in forest plot footnotes (supplementary figures).

Sensitivity analyses were conducted removing studies at high RoB overall (judged to be at high RoB in at least one domain).

## 3 RESULTS

### 3.1 Search results

Initial searches retrieved 17 085 references, plus 246 identified through forward‐citation searching and screening of trial websites, of which 4482 progressed to full‐text screening. A total of 87 studies met our criteria and collected data on mental health outcomes but only 47 studies either reported this data or it was provided by the authors upon request (Figure S1).

### 3.2 Characteristics of included studies

Table 1 displays summary information for the 47 included studies with further details in Supplementary Tables S1 (primary references), S2 (RoB assessments), S3 (key characteristics), S4 (baseline demographics) and S5 (intervention characteristics). Scales that contribute to each mental health outcome category are given in Table S6.

**TABLE 1:** Summary information on characteristics of included studies

Characteristic	Number of studies (total *n* = 47)		
Geographical region	Australia and New Zealand: 5 Europe and the United Kingdom: 13 North America: 28 South America: 1		
Recruitment method	Self‐initiated: 21 Prompted: 15 Required: 0 Not reported: 0		
Intervention content/type, by study arm	(By study arm, *n* = 104) Diet and exercise: 74 Diet only: 14 Exercise only: 2 No diet or exercise (control): 12 Not reported: 2		
Intervention mental health component	(By study arm, *n* = 104) Mental health component included: 41 No mental health component: 63		
Intervention delivery mode	(By study arm, note some arms may include more than one mode) In person: 82 Telephone: 33 Internet: 14 App: 1 Print: 42 Video: 0 Text message: 4 Other: 7		
Intervention setting	(By study arm, note some arms may include more than one setting) Inpatient: 4 Residential: 0 Healthcare: 27 Community: 56 Workplace: 2 Home: 22		
	*Median* (*IQR*)		
Age in years^a^	49.9 (10.5); *n* = 88 study arms; *n* = 39 studies		
Baseline BMI^a^	34.3 kg/m^2^ (4.4); *n* = 82 study arms; *n* = 37 studies		
	*Mean* (*Min–Max*) (*n = 47 studies*) *in months*		
Length of follow‐up for mental Health outcome (months)	19.5 (3–120)		
Programme length (months) (most intensive intervention arm)	5.5 (1.5–12)		
	*Category*	*n = scales*	*n = studies*
Mental health outcome categories	Depression	8	23
	Anxiety	6	8
	Depression and anxiety	5	8
	Self‐esteem	1	6
	Mental health composite score	4	18
	Stress	6	7
	Psychological wellbeing	6	6
	Impact of weight on quality of life	2	1
	Body image	9	5
	Eating disorders	6	7

^a^ Baseline demographics based on total samples randomised at baseline for the primary randomised controlled trial, regardless of whether mental health was only measured in a subset of participants.

### 3.3 Risk of bias

A total of 87 studies reported having measured mental health outcomes, however data was only available for 47. Twenty‐nine of these studies were at unclear RoB, primarily because they did not fully report randomisation procedures, eight at low risk and 10 at high risk (Tables 2 and S2).

**TABLE 2:** RoB summary

RoB domain	Number of studies (*n* = 47)		
	Low risk	Unclear risk	High risk
*Overall RoB*	8	29	10
Selection bias (random sequence generation and allocation concealment)	15	32	0
Detection bias	42	3	2
Attrition bias	41	1	5
Other RoB^a^	—	2	3

^a^ Only assessed where suspected, as per Cochrane guidance. Number of studies listed.

### 3.4 Intervention effects on mental health outcomes

**FIGURE 1:**
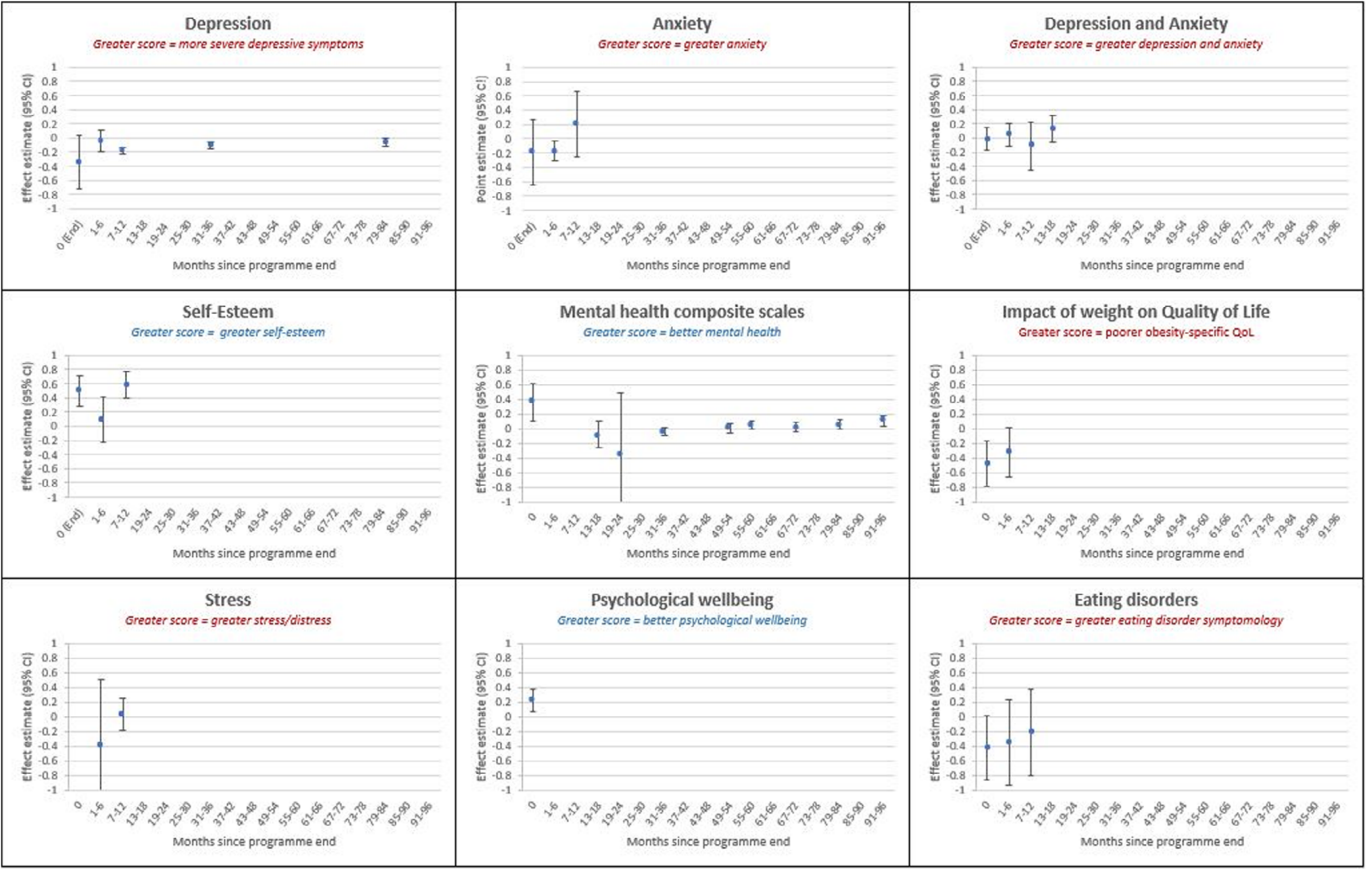
(A–I) Summary of all pooled and singular effect estimates for behavioural weight management programmes (diet and/or exercise) intervention versus control, for each mental health outcome at and after programme‐end. All estimates (including those with one study) are presented as standard mean difference (SMD); Estimates with one study are reported as mean difference in the text.

A summary of all effect estimates is presented in Table 3 unless otherwise stated. Figure 1 depicts effect estimates for comparisons between a BWMP versus control, for each mental health condition. All sensitivity analysis results are presented in Table S7, and head‐to‐head intervention comparisons are reported in the supplementary information.

**TABLE 3:** Effect estimate summary table for mental health outcomes

Follow‐up timepoint at or after programme‐end	Studies (*n*)	Total participants (*n*)	Heterogeneity (I^2^, %)	Effect estimate (SMD/MD^a^ [95% CIs])	See figure
*Depression* (*Intervention* vs. *control*)					
At end	6	5518	92%	−0.34 [−0.71, 0.03]	S2A
1–6 months	8	3180	59%	−0.04 [−0.19, 0.11]	S2B
7–12 months	5	5395	0%	**−0.18 [−0.23, −0.13]**	S2C
19–24 months	2	4901	99%	—	S2D
31–36 months	1	4598	N/A	**−0.24 [−0.37, −0.11]**	S2E
79–84 months	1	4344	N/A	**−0.14 [−0.27, −0.01]**	S2F
*Depression* (*Diet and exercise intervention* vs. *Diet only comparator*)					
At end	3	124	85%	—	S2G
1–6 months	2	289	87%	—	S2H
7–12 months	1	32	N/A	**−7.62 [−10.98, −4.26]**	S2I
13–18 months	2	101	31%	−0.24 [−0.75, 0.27]	S2J
31–36 months	1	32	N/A	−2.52 [−5.30, 0.26]	S2K
*Anxiety* (*Intervention* vs. *Control*)					
At end	1	74	N/A	−0.5 [−1.67, 0.67]	S3A
1–6 months	2	2179	27%	**−0.17 [−0.31, −0.03]**	S3B
7–12 months	1	74	N/A	0.60 [−0.76, 1.96]	S3C
*Anxiety* (*Diet and exercise intervention* vs. *Diet only comparator*)					
At end	1	27	N/A	0.00 [−2.02, 2.02]	S3D
1–6 months	1	213	N/A	0.40 [−0.13, 0.93]	S3E
13–18 months	1	27	N/A	1.00 [−0.21, 2.21]	S3F
*Depression and anxiety* (*Intervention* vs. *Control*)					
At end	4	1017	31%^b^	−0.02 [−0.18, 0.14]	S4A
1–6 months	2	604	0%	0.05 [−0.11, 0.21]	S4B
7–12 months	4	887	77%^b^	−0.11 [−0.45, 0.22]	S4C
13–18 months	1	518	N/A	0.04 [−0.01, 0.09]	S4D
*Depression and anxiety* (*Diet and exercise intervention* vs. *Diet only comparator*)					
At end	1	27	N/A	0.00 [−0.73, 0.73]	S4E
13–18 months	1	27	N/A	0.00 [−1.21, 1.21]	S4F
*Self‐esteem* (*Intervention* vs. *Control*)^c^					
At end	2	836	39%	**0.50 [0.29, 0.71]**	S5A
1–6 months	1	150	N/A	0.30 [−0.71, 1.31]	S5B
7–12 months	1	667	N/A	**0.20 [0.14, 0.26]**	S5C
*Mental health composite score* (*Intervention* vs. *Control*)					
At end	11	7352	94%	**0.36 [0.10, 0.62]**	S6A
1–6 months	4	2909	90%	—	S6B
7–12 months	11	6951	91%	—	S6C
13–18 months	2	616	22%	−0.08 [−0.26, 0.10]	S6D
19–24 months	3	4946	97%	−0.36 [−1.10, 0.47]	S6E
31–36 months	1	4594	N/A	−0.11 [−0.27, 0.05]	S6F
48–54 months	1	4503	N/A	0.04 [−0.18, 0.26]	S6G
55–60 months	1	4464	N/A	0.20 [−0.03, 0.43]	S6H
67–72 months	1	4410	N/A	0.04 [−0.11, 0.19]	S6I
79–84 months	1	4364	N/A	**0.25 [0.01, 0.49]**	S6J
91–96 months	1	3565	N/A	**0.41 [0.17, 0.65]**	S6K
103–108 months	1	1917	N/A	0.06 [−0.25, 0.37]	S6L
*Mental health composite score* (*Diet and exercise intervention* vs. *Diet only comparator*)					
At end	2	437	0%	**0.31 [0.12, 0.50]**	S6M
1–6 months	1	213	N/A	−0.20 [−1.17, 0.77]	S6N
7–12 months	1	265	N/A	−0.10 [−0.98, 0.78]	S6O
*Mental health composite score* (*Diet and exercise intervention* vs. *Exercise only comparator*)					
At end	2	377	98%	—	S6P
1–6 months	1	93	N/A	0.30 [−0.25, 0.85]	S6Q
7–12 months	1	270	N/A	0.00 [−0.76, 0.76]	S6R
*Stress* (*Intervention* vs. *Control*)					
1–6 months	2	486	95%	−0.38 [−1.27, 0.50]	S7A
7–12 months	1	307	N/A	0.17 [−0.79, 1.13]	S7B
*Stress* (*Diet and exercise intervention* vs. *Diet only comparator*)					
1–6 months	1	213	N/A	**−0.42 [−0.61, −0.23]**	S7C
*Psychological wellbeing* (*Intervention* vs. *Control*)					
At end	2	738	0%	**0.22 [0.07, 0.36]**	S8A1
At end^d^	2	373	52%	**0.52 [0.18, 0.87]**	S8A2
7–12 months	2	710	92%	—	S8B1
7–12 months^d^	2	710	90%	—	S8B2
*Impact of weight on QoL* (*Intervention* vs. *Control*)					
At end	1	162	N/A	**−3.20 [−5.24, −1.16]**	S9A
1–6 months	1	150	N/A	**−3.50 [−6.92, −0.08]**	S9B
*Impact of weight on QoL* (*Diet and exercise intervention* vs. *Exercise only comparator*)					
At end	1	109	N/A	**−1.40 [−1.95, −0.85]**	S9C
1–6 months	1	93	N/A	**0.80 [0.19, 1.41]**	S9D
*Eating disorders* (*Intervention* vs. *Control*)					
At end	2	207	44%	−0.42 [−0.85, 0.01]	S11A
1–6 months	2	195	67%	−0.35 [0.93, 0.23]	S11B
7–12 months	1	44	N/A	−0.10 [−0.37, 0.17]	S11C
*Eating disorders* (*Diet and exercise intervention* vs. *Diet only comparator*)					
At end	1	65	N/A	**0.20 [0.09, 0.31]**	S11D
1–6 months	1	76	N/A	**0.30 [0.14, 0.46]**	S11E
13–18 months	1	74	N/A	**0.20 [0.04, 0.36]**	S11F

Mental Health Scale direction	
*Depression*: higher score = worse depression	
*Anxiety*: higher score = greater anxiety	
*Depression and Anxiety*: higher score = greater depression and anxiety	
*Self‐esteem*: higher score = higher self‐esteem	
*Mental health composite score*: higher score = better mental health
*Stress*: higher score = greater stress/distress	
*Psychological wellbeing*: higher score = greater psychological wellbeing
*Impact of weight on QoL*: higher score = poorer weight related QoL	
*Eating disorders*: higher score = greater eating disorder symptomology or binge eating tendencies

*Note*: ‘—’ not appropriate to present pooled estimate. Bolded effect estimates are statistically significant: *p*‐value < 0.05.

Abbreviations: CIs, confidence intervals; MD, mean difference; n, numbers; N/A, not applicable; QoL, quality of life; S, supporting information; SMD, standard mean difference.

^a^ MD are presented when only one study contributes data; SMDs used where multiple studies contribute data.

^b^ I^2^ reduces to 0% when high risk of bias study is removed (see Table S7).

^c^ SMDs used where multiple studies contribute data as some studies reported a normalised Rosenberg Self‐Esteem Scale.

^d^ Same study sample with different measure of psychological wellbeing.

#### 3.4.1 Depression

Eight scales measuring depression were extracted from 23 studies (higher score = worse depression symptoms).

##### BWMP versus control

Twelve studies compared a BWMP to no or minimal intervention control,^13^, ^14^, ^15^, ^16^, ^17^, ^18^, ^19^, ^20^, ^21^, ^22^, ^23^, ^24^ with no evidence of a difference in depression between groups at programme end, although the direction of effect favoured greater reduction in depression in the BWMP (SMD −0.34 [−0.71, 0.03]; I^2^ = 92%), seemingly when greater weight loss was achieved (Figure S2A). The heterogeneity (92%) was not explained by subgrouping by comparator intensity, but as differences were due to magnitude rather than direction of effect, we present a pooled estimate. Sensitivity analyses removing studies at high RoB did not change the direction of effect (Table S7).

There was no evidence of a difference in changes in mean depression scores between BWMPs and control at 1–6 months after programme‐end (SMD −0.04 [−0.19, 0.11]; I^2^ = 59%; Figure S2B); this did not change when a study at high RoB was removed (Table S7).^19^ Heterogeneity (I^2^ = 59%) was somewhat reduced by subgrouping by control group intensity. At 7–12 months after programme‐end, the pooled effect estimate favoured greater reduction in depression in the BWMP versus control (SMD −0.18 [−0.23, −0.13]; I^2^ = 0%; Figure S2C), with 95% CIs excluding no difference. Removing one study due to high RoB did not change the direction of effect however 95% CIs included the possibility of no difference as well as favouring the control (Table S7).^24^

At 19–24 months, there were only two studies with considerable unexplained heterogeneity (I^2^ = 99%; Figure S2D). One study showed greater reduction in depression in the control group (SMD 2.24 [1.92, 2.57]),^13^ while the second study favoured the intervention group (SMD –0.15 [−0.21, −0.09]), however this study was at high RoB.^24^

One study, at high RoB, measured change in depression scores at 31–36 months and 79–84 months after programme‐end.^24^ At 31–36 months, the direction of effect and 95% CI favoured the intervention group (MD −0.24 [−0.37, −0.11]). Improvements in depression persisted at 79–84 months after programme‐end in the BWMP compared with control (MD −0.14 [−0.27, −0.01]).

One study reported absolute median (IQR) depression scores and could not be included in the statistical synthesis.^17^ At programme‐end, a greater reduction in depression was reported in the BWMP intervention compared with control (−2.0 vs. 0.50; *p* = .06), however no differences were reported at 7–12 months after programme‐end.^17^

##### Direct comparisons between BWMPs

Four studies compared a diet and exercise intervention to a diet only comparator.^21^, ^25^, ^26^, ^27^ For estimates at programme‐end and at 1–6 months later, 95% CIs included no difference and there was considerable unexplained heterogeneity (I^2^ = 85% and 87%, respectively; Figure S2G,H). At 7–12 months, a single study showed a greater reduction in depression in the diet and exercise arm versus diet only comparator (MD −7.62 [−10.98, −4.26]; Figure S2I).^27^ This direction of effect continued at 13–18 and 31–36 months after programme‐end however the 95% CI included the possibility of favouring diet alone ((SMD –0.24 [−0.75, 0.27]; I^2^ = 31%); MD −2.52 [−5.3, 0.26]; Figure S2J,K). No studies were at high overall RoB. No studies compared a diet and exercise intervention to an exercise only comparator.

Eight studies made head‐to‐head intervention comparisons between BWMPs.^27^, ^28^, ^29^, ^30^, ^31^, ^32^, ^33^, ^34^ At programme‐end, one study found reduced depression after a self‐guided leaflet‐based intervention (top 10 tips) compared to an intervention focussed on increasing behavioural flexibility by breaking daily habits (Figure S2L).^31^ Another study found reduced depression after a cognitive behavioural therapy (CBT) versus behavioural weight loss treatment, which persisted to between 7 and 12 months after programme‐end (Figure S2N).^34^ At 1–6 months after programme‐end, reduced depression was found after a group versus mail‐delivered non‐dieting programme, which persisted at 7–12 months after programme‐end.^33^ At 31–36 months after programme‐end, reduced depression was observed after a very low‐calorie diet plus behavioural therapy intervention versus behavioural therapy alone^27^ (Figure S2P). No other head‐to‐head intervention comparisons found differences at any other timepoints measured (Figure S2L–Q).

#### 3.4.2 Anxiety

Eight studies using six different scales measured change in anxiety scores, with a higher score indicating greater anxiety.^19^, ^21^, ^26^, ^31^, ^33^, ^34^, ^35^, ^36^

##### BWMP versus control

Three studies included a no/minimal control group.^19^, ^21^, ^35^ One measured anxiety at programme‐end where the point estimate favoured a greater reduction in anxiety in the BWMP but the 95% CI included the possibility of favouring the control (MD –0.5 [−1.67, 0.67]; Figure S3A).^35^ Between 1 and 6 months after programme‐end, a decrease in anxiety favoured the BWMP (SMD −0.17 [−0.31, −0.03], I^2^ = 27%; Figure S3B). Removing the one study at high RoB did not significantly alter the estimate.^19^ By 7–12 months after programme‐end, the point estimate favoured the control, but the 95% CI included the possibility of favouring the BWMP intervention (MD 0.6 [−0.76, 1.96]; Figure S3C).

##### Direct comparisons between BWMPs

Single studies each at programme‐end,^26^ and 1–6 months^21^ and 13–18 months^26^ after programme‐end showed 95% CIs that included the possibility of no difference between BWMP and diet only groups, however point estimates favoured diet only after programme end. None of the studies were at high overall RoB. No studies compared a diet and exercise intervention to an exercise only comparator.

Three studies made other direct comparisons between BWMPs; one found reduced anxiety in a CBT versus behavioural weight loss treatment at programme‐end which persisted at 7–12 months after programme‐end.^34^ Another study favoured the self‐guided leaflet‐based intervention compared to an intervention focussed on breaking daily habits at programme‐end.^31^ The final study found no differences between a mail‐delivered ‘non‐dieting’ program compared with a group ‘non‐dieting’ programme, and a relaxation response training group ‘non‐dieting’ programme, at any other timepoints (Figure S3G–I).^33^

#### 3.4.3 Combined depression and anxiety

Eight studies using five different outcomes measured change in combined depression and anxiety scores, with a higher score indicating greater depression and anxiety.^15^, ^16^, ^20^, ^26^, ^37^, ^38^, ^39^, ^40^

##### BWMP versus control

Six studies compared to a no/minimal control.^15^, ^16^, ^20^, ^37^, ^39^, ^40^ No evidence of difference in depression and anxiety scores across intervention and control groups were found at programme‐end (SMD –0.02 [−0.18, 0.14]; I^2^ = 31%; Figure S4A), 1–6 months after (SMD 0.05 [−0.11, 0.21]; I^2^ = 0%; Figure S4B) or at 7–12 months after programme‐end (SMD –0.11 [−0.45, 0.22]; I^2^ = 77%; Figure S4C). One study was at high RoB and added considerable heterogeneity.^37^ When excluded, the direction of effect changed but 95% CIs overlapped (Table S7).

One study compared a BWMP to usual care (general guideline‐based diet and exercise advice) but was unable to be included in the statistical synthesis.^39^ At programme‐end and at 7–12 months after programme‐end, both study arms reported improvements in depression and anxiety compared to baseline (baseline median [IQR]: usual care: 13 [6–19] vs. BWMP: 11 [7–19]; programme‐end: usual care: 9 [6–16], vs. BWMP: 8 [5–16]; 7–12 months after: usual care: 9 [4–18], vs. BWMP: 9 [5–15]). This study was at high RoB.^39^

##### Direct comparisons between BWMPs

One study showed no difference in changes in depression and anxiety between BWMP and diet only comparison groups at programme‐end (MD 0.00 [−0.73, 0.73]) and 13–18 months after programme‐end (MD 0.00 [95%CI –1.21, 1.21]).^26^ No studies compared a diet and exercise intervention to an exercise only comparator.

Three studies made other direct comparisons between BWMPs^37^, ^38^, ^40^; one found reductions in depression and anxiety after a group‐based CBT lifestyle intervention versus individualised dietetic treatment at programme‐end, however this study was at high RoB.^37^ Intervention comparisons between weight loss versus weight neutral programs, and a lifestyle intervention delivered via phone versus internet, showed no differences at any other timepoints (Figure S4G–J).

#### 3.4.4 Change in depression and/or anxiety over time

In the 28 studies (*n* = 10 785, 33 intervention arms) reporting data on depression and/or anxiety, programme‐end MD (95% CI) in standardized depression and/or anxiety mental health outcome scores between intervention versus comparator groups was −0.45 (−0.81, −0.08), indicating better outcomes in intervention than comparator groups; MD in weight was −2.44 kg (SD 3.05) between intervention and comparator groups.^13^, ^14^, ^15^, ^16^, ^17^, ^18^, ^19^, ^20^, ^21^, ^22^, ^23^, ^24^, ^25^, ^26^, ^27^, ^28^, ^29^, ^30^, ^31^, ^32^, ^33^, ^34^, ^35^, ^36^, ^37^, ^38^, ^39^, ^40^

**FIGURE 2:**
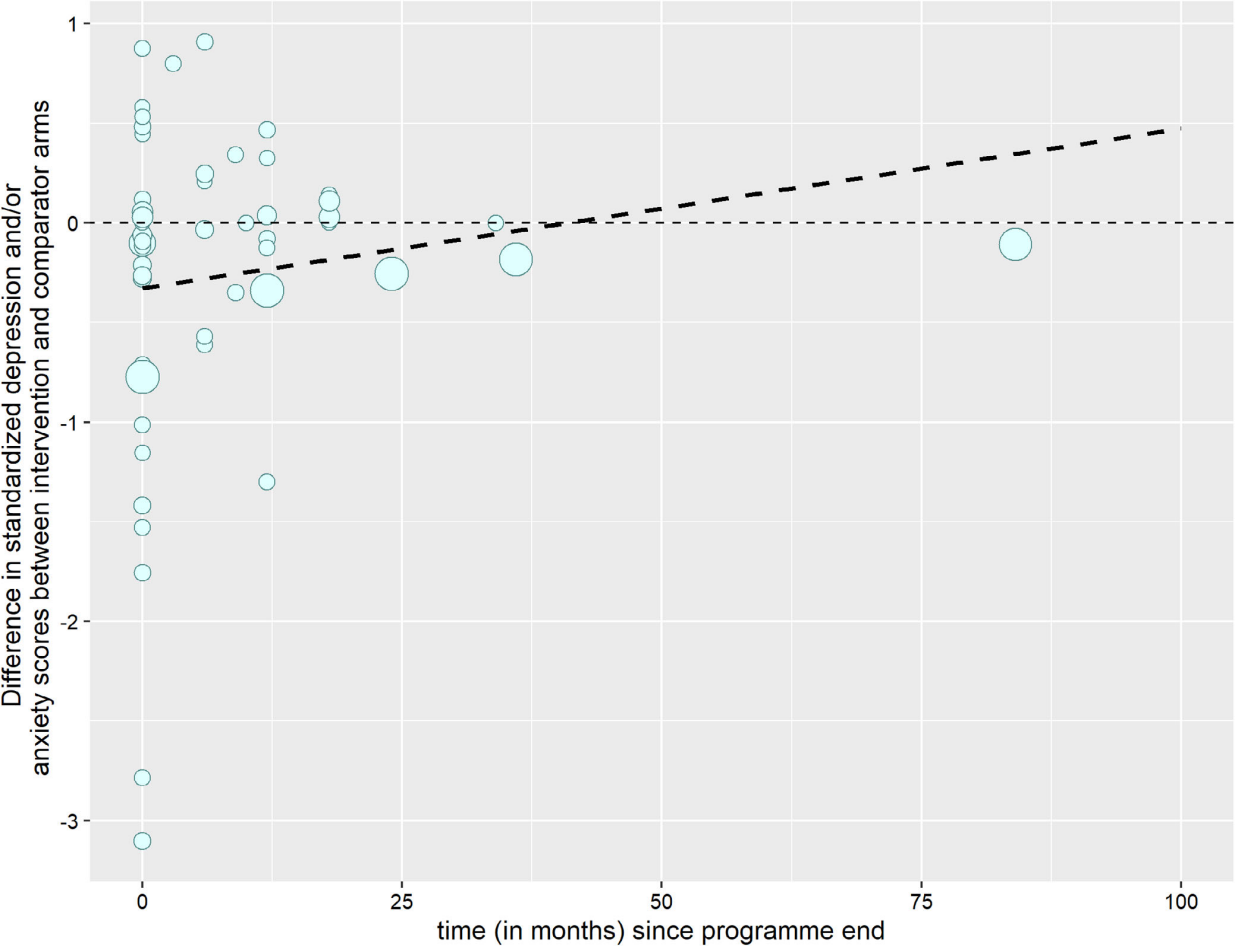
Difference in standardized depression and/or anxiety scores between intervention and comparator arms by time since programme‐end. Dot size is proportional to number of participants in each study. Dashed line represents estimates of average trend from random effects model.

**FIGURE 3:**
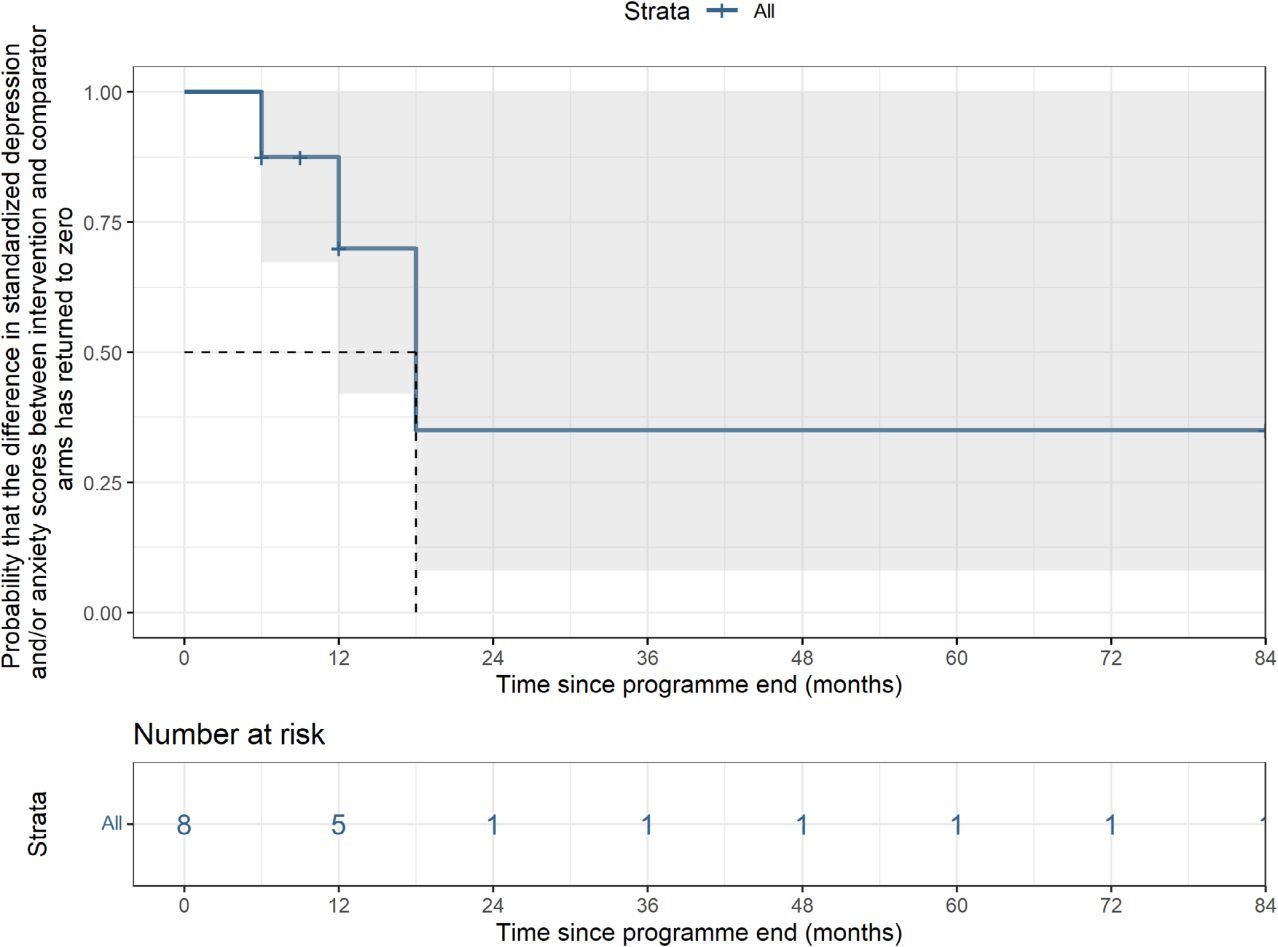
Kaplan Meier plot showing time for intervention group standardised mean depression and/or anxiety scores to reach that of the comparator group.

The mixed model estimated an average increase in standardised depression and/or anxiety relative to control after programme‐end of 0.008 (−0.0007 to 0.0169) per month (Figure 2). In meta‐regression, average change (relative to control) after programme‐end was similar, at 0.010 (−0.010 to 0.031) per month. Higher scores indicate greater depression and anxiety, however in both cases CIs included the possibility of no difference. The time‐to‐event model (Figure 3) showed that the median time to reach no difference in standardised depression and/or anxiety scores between intervention and comparator was 18‐months after programme‐end. Removing studies at high RoB slightly increased the estimate of average trend in change of anxiety and/or depression over time for the random effects (from 0.008 to 0.0146; 95% –0.0047 to 0.0352) and meta‐regression (from 0.010 to 0.027; 95% CI –0.014 to 0.068) models. After removing studies at high RoB from the time‐to‐event model, the median time could not be estimated as anxiety and/or depression outcomes did not return to no difference in at least half these studies.

Every 1 kg of weight regain in the intervention relative to comparator was associated with a 0.024 increase in standardised depression and/or anxiety score units relative to control, but CIs were wide and included no difference between groups (−0.097 to 0.145).

#### 3.4.5 Self‐esteem

Six studies measured self‐esteem using the Rosenberg Self‐Esteem Scale.^18^, ^28^, ^30^, ^38^, ^41^, ^42^ For this analysis, a higher score indicates higher self‐esteem.

Two compared to a no/minimal control group.^18^, ^41^ At programme‐end, greater improvements in self‐esteem were seen in the intervention group and the CIs excluded no difference (SMD: 0.50 [0.29, 0.71; I^2^ = 39%]; Figure S5A). Sensitivity analysis removing one study at high RoB did not significantly alter these findings (Table S7).^41^ At 1–6 months after programme‐end, one study showed that self‐esteem remained higher after the intervention, however CIs include the possibility of no difference or favouring the control (MD 0.30 [−0.71, 1.31]; Figure S5B).^18^ By 7–12 months after programme‐end, one study continued to favour the intervention group (MD 0.20 [0.14, 0.26]; Figure S5C), however this study was at high RoB.^41^

##### Direct comparisons between BWMPs

No studies compared a diet and exercise intervention to a diet only comparator or an exercise only comparator. Four studies made direct comparisons between BWMPs; none found differences (Figure S5D–G).^28^, ^30^, ^38^, ^42^

#### 3.4.6 Mental health composite scores

Eighteen studies measured the mental health component of a general health‐related QoL questionnaire (higher score = better mental health).^13^, ^17^, ^19^, ^21^, ^24^, ^35^, ^39^, ^40^, ^41^, ^43^, ^44^, ^45^, ^46^, ^47^, ^48^, ^49^, ^50^, ^51^

##### BWMP versus control

Fourteen compared a BWMP intervention to a control group.^13^, ^17^, ^19^, ^21^, ^24^, ^35^, ^39^, ^40^, ^41^, ^44^, ^47^, ^48^, ^50^, ^51^ At programme‐end, there was evidence of improved mental health (SMD of 0.36 [0.10, 0.62]; I^2^ = 94%; Figure S6A), however there was considerable unexplained heterogeneity. Removing three studies at high RoB did not change the direction of effect of the pooled estimate, however created a significant difference between control subgroups (I^2^ = 85.9%; Table S7).^24^, ^39^, ^41^ Funnel plot asymmetry indicates the possibility of non‐reporting bias or other bias, so this finding must be considered with caution (Figure S6A1).

At 1–6 months after programme‐end, there was considerable unexplained heterogeneity (I^2^ = 90%). Of the three studies not at high RoB, two studies^40^, ^47^ showed 95% CIs including no difference while one study^21^ favoured the intervention.

Similarly, considerable, unexplained heterogeneity (I^2^ = 91%; Figure S6C) remained 7–12 months after programme‐end. Most studies during this period found no difference, except for three studies which found better mental health after BWMP intervention (SMD 4.04 [3.23, 4.85]; SMD 0.43 [0.02, 0.85]; SMD 0.46 [0.06, 0.85]),^35^, ^37^, ^39^, ^48^ and another study indicated better mental health in the control but included the possibility of no difference (SMD ‐0.06 [−0.12, −0.00]).^24^

At 13–18 months after programme‐end, the direction of effect favoured the control, suggesting adverse effects of the BWMP. However, 95% CIs could not exclude the possibility of no difference or favouring of the intervention (SMD –0.08 [−0.26, 0.10]; I^2^ = 22%; Figure S6D). This pattern remained at 19–24 months after programme‐end (SMD –0.36 [−1.20, 0.47]; I^2^ = 97%; Figure S6E), however there was considerable heterogeneity observed between subgroups (I^2^ = 96.8%). Two studies comparing BWMP to control groups 1 and 3 included the possibility of no difference while a third study favoured control group 2 (Figure S6E).^13^, ^24^, ^48^ The removal of one study at high RoB did not meaningfully change the pooled estimate.^24^

One study reported data at multiple time points between 31 and 108 months after programme‐end and found no difference in the change in mental health composite scores across most of these time points; the intervention arm was favoured at 79–84 months (MD 0.25 [0.01, 0.49]) and 91–96 months after programme‐end (MD 0.41 [0.17, 0.65]), however this effect was small (Figure S6F–L).^24^

#### 3.4.7 Direct comparisons between BWMPs

##### Diet and exercise intervention versus diet only

Two studies compared a diet and exercise intervention to a diet only comparator.^46^, ^49^ At programme‐end, a SMD of 0.31 ([0.12, 0.50]; I^2^ = 0%; Figure S6M) favoured the diet and exercise interventions and no statistical heterogeneity was observed. Removal of one study at high RoB did not change this effect.^46^ Single studies reported at 1–6 and 7–12 months after programme‐end included the possibility of no difference between study groups, however the direction of effect favoured diet only (Figure S6N–O).

##### Diet and exercise intervention versus exercise only

Two studies compared a diet and exercise intervention to an exercise only comparator.^43^, ^49^ At programme‐end, there was substantial unexplained statistical heterogeneity (Figure S6P). At 1–6 and 7–12 months after programme‐end, single studies found no evidence of a difference in mental health composite scores (Table 3).

##### Head‐to‐head intervention comparisons

Six studies made other direct comparisons between BWMPs.^40^, ^43^, ^44^, ^45^, ^49^, ^51^ One found improved mental health in an established lifestyle intervention including diet and exercise intervention plus Dietary Approaches to Stop Hypertension (DASH) dietary pattern intervention compared with the established lifestyle intervention alone, at programme‐end.^44^ At 1–6 months after programme‐end, one study found improved mental health with an intervention focused on weight loss rather than weight maintenance.^43^ No other comparisons found differences at any other timepoints measured (Figure S6S–V).

#### 3.4.8 Stress

Seven studies measured change in perceived stress or psychological distress using six different scales (higher score = greater stress/distress).^20^, ^21^, ^33^, ^42^, ^52^, ^53^, ^54^ No studies were at high RoB.

##### BWMP versus control

Two studies compared a BWMP intervention to a no/minimal control group 1.^20^, ^21^ No information was available at programme‐end. At 1–6 months after, the direction of effect favoured the intervention, however CIs were wide (SMD –0.38 [−1.27, 0.50]; I^2^ = 95%). Considerable heterogeneity was observed, although this may be explained by weight change differences (Figure S7A). At 7–12 months after programme‐end, CI for one study included the possibility of no difference in stress (MD 0.17 [−0.79, 1.13]; Figure S7B).^20^

##### Direct comparisons between BWMPs

At 1–6 months after programme‐end, one study suggested a reduction in perceived stress/destress in the diet and exercise intervention versus diet only comparator (MD –0.42 [95% CI –0.61, −0.23]; Figure S7C).^21^ No studies compared a diet and exercise intervention to an exercise only comparator.

Five studies made other direct comparisons between BWMPs.^33^, ^42^, ^52^, ^53^, ^54^ At programme‐end, one study showed reduced stress after a mindfulness intervention versus an active control intervention,^53^ while another study showed reduced psychological distress after a brief strategic therapy over a CBT intervention up to 12 months after programme‐end.^54^ No other head‐to‐head intervention comparisons found differences (Figure S7D–G).

#### 3.4.9 Psychological wellbeing

Six studies using six different scales measured change in psychological wellbeing measured through mood, psychological wellbeing (QoL, ability to cope, ease of decision making, personal value and happiness), satisfaction with life (higher score = greater psychological wellbeing).^31^, ^34^, ^37^, ^41^, ^55^, ^56^

##### BWMP versus control

Two studies compared a BWMP intervention to a no/minimal control group 1; one of these studies reported two scales of psychological wellbeing and are reported separately in Table 3.^37^, ^41^ At programme‐end, an increase in wellbeing scores favoured the intervention group, however one pooled estimate is limited by moderate heterogeneity and both studies are at high RoB. Substantial unexplained heterogeneity (I^2^ = 92%; 90%; Figure S8B1–B2) was observed at 7–12 months after programme‐end; no pooled estimate has been presented.

##### Direct comparisons between BWMPs

No studies compared a diet and exercise intervention to a diet only comparator or an exercise only comparator.

Five studies directly compared BWMPs.^31^, ^37^, ^41^, ^55^, ^56^ One showed improvements in psychological wellbeing scores in an exercise‐support protocol with group nutrition session intervention compared with a print manual plus telephone follow‐up intervention at programme‐end, and at 1–6 months and 13–18 months after programme‐end.^56^ Another study also showed improvements in a social cognitive theory‐based weight‐management treatment delivered via group sessions over a written manual and phone support at the same timepoints.^55^

A further study found improved psychological wellbeing scores after a CBT versus behavioural weight loss treatment intervention at programme‐end and at 7–12 months after programme‐end.^34^ No other comparisons found differences at any other timepoints (Figure S8C–F).

#### 3.4.10 Diet and weight‐related mental health outcome measures

Findings on diet and weight‐related mental health outcome including measures of body image, eating disorders and impact of weight on quality of life are reported in the supplementary material. No pooled estimates showed clear evidence of a between‐group difference at any time point.

## 4 DISCUSSION

Pooled estimates in this review found no evidence of mental health harm after weight loss in a BWMP, irrespective of weight regain after programme end, although much of the evidence was uncertain. There was no evidence that weight regain was associated with change in anxiety/depression scores relative to comparator groups, and modelled changes in depression and/or anxiety scores over time were inconclusive.

### 4.1 BWMP (diet and/or exercise) intervention versus control

When comparing BWMPs (diet and/or exercise) to control groups, some analyses showed improvements in mental health outcomes at or after programme‐end. Pooled estimates for psychological wellbeing, self‐esteem and mental health composite scores at programme‐end, anxiety at 1–6 months, and depression at 7–12 months after programme‐end suggested possible improvements, with 95% CIs excluding no difference. No pooled estimates suggested mental health harm and had 95% CIs excluding no difference. For all other measures at all other timepoints, 95% CIs included the possibility of no difference or could not be meta‐analysed (included only one study).

A previous systematic review found evidence of improvements in depression at programme‐end and up to 12‐months from baseline.^2^ Our findings suggest that improvements in depression continue at 7–12 months after the end of a BWMP (follow‐up from baseline ranging from 12 to 24 months), suggesting that improvements in depression are not solely due to therapeutic effects during the BMWP. Jones et al. also found no evidence of difference in anxiety at programme end,^2^ whereas our findings suggest reductions in anxiety at 1–6 months after a BWMP compared to control. We also found improvements in self‐esteem scores at the end of a BWMP. No evidence of difference was reported by Jones et al., although the direction of effect favoured the BWMP.^2^

A previous review reported improvements in general mental health after intentional weight loss^2^ and an individual patient data meta‐analysis showed that decreases in BMI were associated with higher health‐related QoL for people with a BMI >25 kg/m^2^.^57^ Here, we also show improvements in general mental health, in addition to psychological wellbeing at programme end.

### 4.2 Isolating the impacts of diet and activity

There were few differences in mental health outcomes when comparing BWMPs including diet and exercise to diet only comparators. However, a pooled estimate for mental health composite scores at programme‐end favoured the combined diet and exercise intervention over diet alone comparators, with 95% CIs excluding no difference. Meta‐analyses were not possible for longer follow‐up timepoints.

A previous review found no benefit of exercise on mental health QoL or depression over control.^5^ More recently, Carraca et al.^6^ investigated the effect of exercise training on psychological outcomes in adults with overweight/obesity with no difference in overall mental health QoL, however sub‐scores of mental health QoL favoured the exercise intervention.^6^ Findings from our review support the latter, with a pooled estimate of two studies suggesting improvements in mental health QoL at programme‐end for combined diet and exercise interventions compared to diet only. Carraca et al.^6^ also found no effects of exercise on depression pre‐ versus post‐intervention, which align with the pattern of evidence on depression found between 13 and 36 months after programme‐end in this review.

### 4.3 Strengths and limitations

To our knowledge this is the first review to present a comprehensive and systematic summary and synthesis of the available evidence on the longer‐term impact (≥12 months) of BWMPs on mental health outcomes after programme‐end, as well as modelling its change with relation to weight regain.

This review has some limitations. Our comprehensive search strategy which included hand‐searching and contacting authors meant our latest search was run in December 2019 and studies conducted since are not included. However, our efforts to contact authors of unpublished studies meant some of this data was available to us prior to publication. As our research question focussed in part on longer‐term outcomes, studies had to follow participants for ≥12 months from baseline and after programme‐end for inclusion. This means that results at programme‐end may not be informed by all the available evidence. Differences in weight change and subgroup comparisons determined by the nature of the control group are observational in nature, and at risk of the biases inherent to cohort studies.

Some studies were deemed at high RoB, however their impact on our results were explored through sensitivity analyses (Table S7). A funnel plot showed some asymmetry for mental health composite scores at programme end, suggesting possible publication or other RoB. There were insufficient studies to create funnel plots for other analyses. Selective reporting may have occurred given 40 studies reported collecting mental health outcomes that were not reported and could not be obtained from the authors, diminishing certainty in the pooled estimates. On average, studies that reported data were larger than those that did not (397 vs. 271 participants).

Other limitations of this review are common to research in this field. The subjective approach used to categorise psychological outcome scales for a particular mental health condition, limits the ability to compare research findings.

Comparison of meta‐analyses of dynamic mental health outcomes is hampered because different studies contributed data at different follow‐up points. Our meta‐regression partially addresses this by examining within‐study time trends, but agreed reporting standards regarding methods and time points for mental health outcomes in this field would allow greater clarity.

## 5 CONCLUSIONS

These pooled analyses provide reassurance that, on average, BWMPs do not cause mental health harm, either at or after programme‐end. There is a suggestion that BWMPs may improve some dimensions of mental health after programme‐end, but further research is needed. Further evidence syntheses may benefit from using individual patient data to explore the extent to which weight loss and personal characteristics may impact the relationship between BWMPs and mental health outcomes.

## AUTHOR CONTRIBUTIONS

Jamie Hartmann‐Boyce, Paul Aveyard, Susan A. Jebb and Anastasios Bastounis conceived and designed the review with contributions from Annika Theodoulou and Ailsa R. Butler. Annika Theodoulou and Jamie Hartmann‐Boyce drafted the manuscript. Annika Theodoulou and Jordan Gorenberg conducted screening and data extraction. Annika Theodoulou conducted the main statistical analyses and Jason L. Oke conducted post hoc analyses. Annika Theodoulou and Jamie Hartmann‐Boyce prepared the first draft of the review, with further input from Paul Aveyard and Susan A. Jebb and all authors contributed to the interpretation and final write up.

## FUNDING INFORMATION

This research is a secondary analysis of a primary project funded by the British Heart Foundation and the National Institute for Health Research Oxford Biomedical Research Centre. Paul Aveyard and Susan A. Jebb are an NIHR senior investigators and are funded by NIHR Oxford Biomedical Research Centre and NIHR Oxford and Thames Valley Applied Research Collaboration.

## CONFLICT OF INTEREST

Annika Theodoulou, Jamie Hartmann‐Boyce, Jordan Gorenberg, Jason L. Oke, Ailsa R. Butler and Anastasios Bastounis declare no conflicts of interest. Paul Aveyard and Susan A. Jebb were investigators on an unrelated trial in which Nestle donated products to NHS patients.

## Supporting information


**Data S1.** Supporting information.
