# Supplementary material for: Weight regain and mental health outcomes following behavioural weight management programmes: A systematic review with meta‐analyses
Source: Clin Obes. 2023 Jan 9;13(3):e12575. doi: 10.1111/cob.12575 (PMC10909518; doi:10.1111/cob.12575)
Supplement: Supplementary file 1 — Data S1. Supporting information. [file COB-13-e12575-s001.pdf]

SUPPORTING INFORMATION

Weight regain and mental health outcomes following behavioural weight management programmes: A systematic review with meta-analyses

Annika Theodoulou, MClinSc
Nuffield Department of Primary Care Health Sciences, University of Oxford, Oxford, UK
Jamie Hartmann-Boyce, DPhil
Nuffield Department of Primary Care Health Sciences, University of Oxford, Oxford, UK
Jordan Gorenberg
Nuffield Department of Primary Care Health Sciences, University of Oxford, Oxford, UK
Jason L. Oke, DPhil
NIHR Oxford Biomedical Research Centre, Oxford University Hospitals NHS Foundation Trust
Ailsa R Butler, DPhil
Nuffield Department of Primary Care Health Sciences, University of Oxford, Oxford, UK
Anastasios Bastounis, PhD
Nuffield Department of Primary Care Health Sciences, University of Oxford, Oxford, UK.
Division of Epidemiology and Public Health, School of Medicine, University of Nottingham, Nottingham, UK
Susan A Jebb*, PhD, FRCP (Hon), FMedSci
Nuffield Department of Primary Care Health Sciences, University of Oxford, Oxford, UK
Paul Aveyard*, PhD FRCP FRCGP FFPH
Nuffield Department of Primary Care Health Sciences, University of Oxford, Oxford, UK
*Joint senior

Corresponding author:

Annika Theodoulou
Nuffield Department of Primary Care Health Sciences, University of Oxford
Radcliffe Observatory Quarter, Woodstock Road, Oxford, OX2 6GG
annika.theodoulou@phc.ox.ac.uk

Contents

SUPPLEMENTARY TABLES 6

TABLE S1. INCLUDED STUDIES REFERENCE LIST 6
TABLE S2. RISK OF BIAS OF INCLUDED STUDIES 10
TABLE S3. CHARACTERISTICS OF INCLUDED STUDIES 29
TABLE S4. BASELINE DEMOGRAPHICS 34
TABLE S5. INTERVENTION CHARACTERISTICS 38
TABLE S6. MENTAL HEALTH OUTCOME SCALE CATEGORIES 58
TABLE S7. SENSITIVITY ANALYSES 59

SUPPLEMENTARY FIGURES 61

FIGURE S1. PRISMA DIAGRAM OF STUDY FLOW 61
DEPRESSION 62
Outcome measures: 62
Comparison 1 – BWMP (diet and/or exercise) versus Control group 1-4 62
Figure S2a. Depression at programme end 62
Figure S2b. Depression at 1-6 months after programme end 63
Figure S2c. Depression at 7-12 months after programme end 63
Figure S2d. Depression at 19-24 months after programme end 63
Figure S2e. Depression at 31-36 months after programme end 64
Figure S2f. Depression at 79-84 months after programme end 64
Comparison 2 – BWMP (diet and exercise) versus diet only 64
Figure S2g. Depression at programme end 64
Figure S2h. Depression at 1-6 months after programme end 64
Figure S2i. Depression at 7-12 months after programme end 64
Figure S2j. Depression at 13-18 months after programme end 65
Figure S2k. Depression at 31-36 months after programme end 65
Comparison 3 – BWMP (diet and exercise) versus exercise only 65
No studies 65
Comparison 4 – Intervention Vs Comparator intervention 65
Depression head-to-head intervention comparisons 65
Figure S2l. Depression at programme end 66
Figure S2m. Depression at 1-6 months after programme end 66
Figure S2n. Depression at 7-12 months after programme end 66
Figure S2o. Depression at 13 -18 months after programme end 66
Figure S2p. Depression at 31 -36 months after programme end 67
Figure S2q. Depression at 37 -42 months after programme end 67
ANXIETY 68
Outcome measures: 68
Comparison 1 – BWMP (diet and/or exercise) versus Control group 1-4 68
Figure S3a. Anxiety at programme end 68
Figure S3b. Anxiety at 1-6 months after programme end 68
Figure S3c. Anxiety at 7-12 months after programme end 68
Comparison 2 – BWMP (diet and exercise) versus diet only 69
Figure S3d. Anxiety at programme end 69
Figure S3e. Anxiety at 1-6 months after programme end 69
Figure S3f. Anxiety at 13-18 months after programme end 69
Comparison 3 – BWMP (diet and exercise) versus exercise only 69
No studies 69
Comparison 4 – Intervention Vs Comparator intervention 69
Anxiety head-to-head intervention comparisons 69
Figure S3g. Anxiety at programme end 70
Figure S3h. Anxiety at 1-6 months after programme end 70
Figure S3i. Anxiety at 7-12 months after programme end 70

DEPRESSION AND ANXIETY **71**
Outcome measures: *71*
Comparison 1 – BWMP (diet and/or exercise) versus Control group 1-4 .... *71*
Figure S4a. Depression and Anxiety at programme end *71*
Figure S4b. Depression and Anxiety at 1-6 months after programme end *71*
Figure S4c. Depression and Anxiety at 7-12 months after programme end *72*
Figure S4d. Depression and Anxiety at 13-18months after programme end *72*
Comparison 2 – BWMP (diet and exercise) versus diet only *72*
Figure S4e. Depression and Anxiety at programme end *72*
Figure S4f. Depression and Anxiety at 13-18 months after programme end *72*
Comparison 3 – BWMP (diet and exercise) versus exercise only *72*
No studies *72*
Comparison 4 – Intervention Vs Comparator intervention *72*
Depression and Anxiety head-to-head intervention comparisons *72*
Figure S4g. Depression and Anxiety at programme end *73*
Figure S4h. Depression and Anxiety at 1-6 months after programme end *73*
Figure S4i. Depression and Anxiety at 7-12 months after programme end *73*
Figure S4j. Depression and Anxiety at 13-18 months after programme end *73*
SELF-ESTEEM **74**
Outcome measures: *74*
Comparison 1 – BWMP (diet and/or exercise) versus Control group 1-4 .... *74*
Figure S5a. Self-esteem at programme end *74*
Figure S5b. Self-esteem at 1-6 months after programme end *74*
Figure S5c. Self-esteem at 7-12 months after programme end *74*
Comparison 2 – BWMP (diet and exercise) versus diet only *75*
No studies *75*
Comparison 3 – BWMP (diet and exercise) versus exercise only *75*
No studies *75*
Comparison 4 – Intervention Vs Comparator intervention *75*
Self-esteem head-to-head intervention comparisons *75*
Figure S5d. Self-esteem at programme end *75*
Figure S5e. Self-esteem at 1-6 months after programme end *75*
Figure S5f. Self-esteem at 7 - 12 months after programme end *75*
Figure S5g. Self-esteem at 13 - 18 months after programme end *76*
MENTAL HEALTH COMPOSITE SCALES **77**
Outcome measures: *77*
Comparison 1 – BWMP (diet and/or exercise) versus Control group 1-4 .... *77*
Figure S6a. Component Summaries at programme end *77*
Figure S6b. Component Summaries at 1-6 months after programme end *78*
Figure S6c. Component Summaries at 7-12 months after programme end *79*
Figure S6d. Component Summaries at 13-18 months after programme end *79*
Figure S6e. Component Summaries at 19-24 months after programme end *80*
Figure S6f. Component Summaries at 31-36 months after programme end *80*
Figure S6g. Component Summaries at 49-54 months after programme end *80*
Figure S6h. Component Summaries at 55-60 months after programme end *80*
Figure S6i. Component Summaries at 67-72 months after programme end *80*
Figure S6j. Component Summaries at 79-84 months after programme end *81*
Figure S6k. Component Summaries at 91--96 months after programme end *81*
Figure S6l. Component Summaries at 103--108 months after programme end *81*
Comparison 2 – BWMP (diet and exercise) versus diet only *81*
Figure S6m. Component Summaries at programme end *81*
Figure S6n. Component Summaries at 1-6 months after programme end *81*
Figure S6o. Component Summaries at 7-12 months programme end *81*
Comparison 3 – BWMP (diet and exercise) versus exercise only *82*
Figure S6p. Component Summaries at programme end *82*
Figure S6q. Component Summaries at 1-6 months after programme end *82*
Figure S6r. Component Summaries at 7-12 months programme end *82*

Comparison 4 – Intervention Vs Comparator intervention 82
Mental health composite scales head-to-head intervention comparisons 82
Figure S6s. Component Summaries at programme end 83
Figure S6t. Component Summaries at 1-6 months after programme end 83
Figure S6u. Component Summaries at 7-12 months after programme end 83
Figure S6v. Component Summaries at 13-18 months after programme end 83
STRESS 84
Outcome measures: 84
Comparison 1 – BWMP (diet and/or exercise) versus Control group 1-4 84
Figure S7a. Stress at 1-6 months after programme end 84
Figure S7b. Stress at 7-12 months after programme end 84
Comparison 2 – BWMP (diet and exercise) versus diet only 84
Figure S7c. Stress at 1-6 months after programme end 84
Comparison 3 – BWMPs (diet and exercise) versus exercise only (Control group 5) 85
No studies 85
Comparison 4 – Intervention Vs Comparator intervention 85
Stress head-to-head intervention comparisons 85
Figure S7d. Stress at programme end 85
Figure S7f. Stress at 1 to 6 months after programme end 85
Figure S7g. Stress at 7 to 12 months after programme end 86
PSYCHOLOGICAL WELLBEING 87
Outcome measures: 87
Comparison 1 – BWMP (diet and/or exercise) versus Control group 1-4 87
Figure S8a1. Psychological wellbeing at programme end 87
Figure S8a2. Psychological wellbeing at programme end 87
Figure S8b1. Psychological wellbeing at 7-12 months after programme end 87
Figure S8b2. Psychological wellbeing at 7-12 months after programme end 88
Comparison 2 – BWMP (diet and exercise) versus diet only 88
No studies 88
Comparison 3 – BWMP (diet and exercise) versus exercise only 88
No studies 88
Comparison 4 – Intervention Vs Comparator intervention 88
Psychological wellbeing head-to-head intervention comparisons 88
Figure S8c. Wellbeing at programme end 89
Figure S8d. Wellbeing at 1-6 months after programme end 89
Figure S8e. Wellbeing at 7-12 months after programme end 89
Figure S8f. Wellbeing at 13-18 months after programme end 89
IMPACT OF WEIGHT ON QUALITY OF LIFE (QOL) 90
Outcome measures: 90
Findings 90
Comparison 1 – BWMP (diet and/or exercise) versus Control group 1-4 90
Figure S9a. Impact of weight on QoL at programme end 90
Figure S9b. Impact of weight on QoL after 1-6 months after programme end 91
Comparison 2 – BWMP (diet and exercise) versus diet only 91
No studies 91
Comparison 3 – BWMP (diet and exercise) versus exercise only 91
Figure S9c. Impact of weight on QoL at programme end 91
Figure S9d. Impact of weight on QoL after 1-6 months after programme end 91
Comparison 4 – Intervention Vs Comparator intervention 91
Impact of weight on QoL head-to-head intervention comparisons 91
Figure S9e. Impact of weight on QoL at programme end 91
Figure S9f. Impact of weight on QoL at 1-6 months after programme end 92
BODY IMAGE 93
Outcome measures: 93
Findings 93
Comparison 1 – BWMP (diet and/or exercise) versus Control group 1-4 93

No studies able to be pooled in meta-analysis ... 93
Comparison 2 – BWMP (diet and exercise) versus diet only ... 94
No studies ... 94
Comparison 3 – BWMP (diet and exercise) versus exercise only ... 94
No studies ... 94
Comparison 4 – Intervention Vs Comparator intervention ... 94
Body image head-to-head intervention comparisons ... 94
Figure S10a. Body image at programme end ... 94
Figure S10b. Body image at 1-6 months after programme end ... 94
Figure S10c. Body image at 7-12 months after programme end ... 95
EATING DISORDERS ... 96
Outcome measures: ... 96
Findings ... 96
Comparison 1 – BWMP (diet and/or exercise) versus Control group 1-4 ... 97
Figure S11a. Eating Disorders at programme end ... 97
Figure S11b. Eating Disorders at 1-6 months after programme end ... 97
Figure S11c. Eating Disorders at 7-12 months after programme end ... 97
Comparison 2 – BWMP (diet and exercise) versus diet only ... 97
Figure S11d. Eating Disorders at programme end ... 97
Figure S11e. Eating disorders at 1 to 6 months after ... 97
Figure S11f. Eating disorders at 13 to 18 months after ... 98
Comparison 3 – BWMP (diet and exercise) versus exercise only ... 98
No studies ... 98
Comparison 4 – Intervention Vs Comparator intervention ... 98
Eating disorders head-to-head intervention comparisons ... 98
Figure S11g. Eating Disorders at programme end ... 98
Figure S11h. Eating Disorders at 1-6 months after programme end ... 99
Figure S11i. Eating Disorders at 7-12 months after programme end ... 99
Figure S11j. Eating Disorders at 13-18 months after programme end ... 99
DEVIATIONS FROM PROTOCOL ... 99
REFERENCES ... 100

Abbreviations:

BWMP: Behavioural weight management programme

SUPPLEMENTARY TABLES

Table S1. Included studies reference list

Study ID	Primary references
Ames 2005	Ames GE, Perri MG, Fox LD, et al. Changing weight-loss expectations: a randomized pilot study. *Eat behav* 2005; **6**(3): 259-69.
Andersen 1999	Andersen RE, Wadden TA, Bartlett SJ, Zemel B, Verde TJ, Franckowiak SC. Effects of lifestyle activity vs structured aerobic exercise in obese women: a randomized trial. *JAMA* 1999; **281**(4): 335-40.
Annesi 2016	Annesi JJ, Johnson PH, Tennant GA, Porter KJ, McEwen KL. Weight Loss and the Prevention of Weight Regain: Evaluation of a Treatment Model of Exercise Self-Regulation Generalizing to Controlled Eating. *Perm J* 2016; **20**(3): 15-146.
Annesi 2017	Annesi JJ. Mediation of the relationship of behavioural treatment type and changes in psychological predictors of healthy eating by body satisfaction changes in women with obesity. *Obes Res Clin Pract* 2017; **11**(1): 97-107.
Appel 2011	Appel LJ, Clark JM, Yeh HC, et al. Comparative effectiveness of weight-loss interventions in clinical practice. *N Engl J Med* 2011; **365**(21): 1959-68.<br>Rubin RR, Peyrot M, Wang NY, et al. Patient-reported outcomes in the practice-based opportunities for weight reduction (POWER) trial. *Qual Life Res*. 2013;**22**(9):2389-2398.
Ard 2004	Ard JD, Grambow SC, Liu D, Slentz CA, Kraus WE, Svetkey LP. The effect of the PREMIER interventions on insulin sensitivity. *Diabetes Care* 2004; **27**(2): 340-7.<br>Young DR, Coughlin J, Jerome GJ, Myers V, Chae SE, Brantley PJ. Effects of the PREMIER interventions on health-related quality of life. *Ann Behav Med*. 2010;**40**(3):302-312.
Ard 2018	Ard JD, Gower B, Hunter G, et al. Effects of Calorie Restriction in Obese Older Adults: The CROSSROADS Randomized Controlled Trial. *J Gerontol A Biol Sci Med Sci* 2017; **73**(1): 73-80.
Ash 2006	Ash S, Reeves M, Bauer J, et al. A randomised control trial comparing lifestyle groups, individual counselling and written information in the management of weight and health outcomes over 12 months. *Int J Obes (Lond)* 2006; **30**(10): 1557-64.
Bacon 2002	Bacon L, Keim NL, Van Loan MD, et al. Evaluating a 'non-diet' wellness intervention for improvement of metabolic fitness, psychological well-being and eating and activity behaviors. *Int J Obes Relat Metab Disord* 2002; **26**(6): 854-65.
Barnes 2017	Barnes RD, Ivezaj V, Martino S, Pittman BP, Grilo CM. Back to Basics? No Weight Loss from Motivational Interviewing Compared to Nutrition Psychoeducation at One-Year Follow-Up. *Obesity (Silver Spring)* 2017; **25**(12): 2074-8.
Bennett 2012	Bennett GG, Warner ET, Glasgow RE, et al. Obesity treatment for socioeconomically disadvantaged patients in primary care practice. *Arch Intern Med* 2012; **172**(7): 565-74.
Bennett 2013	Bennett GG, Foley P, Levine E, et al. Behavioral treatment for weight gain prevention among black women in primary care practice: a randomized clinical trial. *JAMA Intern Med* 2013; **173**(19): 1770-7.<br>Foley P, Levine E, Askew S, et al. Weight gain prevention among black women in the rural community health center setting: the Shape Program. *BMC Public Health*. 2012;**12:**305.
Beutel 2006	Beutel ME, Dippel A, Szczepanski M, Thiede R, Wiltink J. Mid-term effectiveness of behavioral and psychodynamic inpatient treatments of severe obesity based on a randomized study. *Psychother Psychosom* 2006; **75**(6): 337-45.
Burke 2015	Burke LE, Ewing LJ, Ye L, et al. The SELF trial: A self-efficacy-based behavioral intervention trial for weight loss maintenance. *Obesity (Silver Spring)* 2015; **23**(11): 2175-82.
Cleo 2018	Cleo G, Glasziou P, Beller E, Isenring E, Thomas R. Habit-based interventions for weight loss maintenance in adults with overweight and obesity: a randomized controlled trial. *Int J Obes (Lond)* 2019; **43**(2): 374-83.

Conroy 2015	Conroy MB, Sward KL, Spadaro KC, et al. Effectiveness of a physical activity and weight loss intervention for middle-aged women: healthy bodies, healthy hearts randomized trial. *J Gen Intern Med* 2015; **30**(2): 207-13.
Damschroder 2014	Damschroder LJ, Lutes LD, Kirsh S, et al. Small-changes obesity treatment among veterans: 12-month outcomes. *Am J Prev Med* 2014; **47**(5): 541-53.
Daubenmier 2016	Daubenmier J, Moran PJ, Kristeller J, et al. Effects of a mindfulness-based weight loss intervention in adults with obesity: A randomized clinical trial. *Obesity (Silver Spring)* 2016; **24**(4): 794-804.<br>Mason AE, Epel ES, Aschbacher K, et al. Reduced reward-driven eating accounts for the impact of a mindfulness-based diet and exercise intervention on weight loss: Data from the SHINE randomized controlled trial. *Appetite*. 2016;**100**:86-93.
de Zwaan 2017	de Zwaan M, Herpertz S, Zipfel S, et al. Effect of Internet-Based Guided Self-help vs Individual Face-to-Face Treatment on Full or Subsyndromal Binge Eating Disorder in Overweight or Obese Patients: The INTERBED Randomized Clinical Trial. *JAMA psychiatry* 2017; **74**(10): 987-95.
deRoon 2017	de Roon M, van Gemert WA, Peeters PH, Schuit AJ, Monninkhof EM. Long-term effects of a weight loss intervention with or without exercise component in postmenopausal women: A randomized trial. *Prev Med Rep* 2017; **5**: 118-23.<br>van Gemert WA, van der Palen J, Monninkhof EM, et al. Quality of Life after Diet or Exercise-Induced Weight Loss in Overweight to Obese Postmenopausal Women: The SHAPE-2 Randomised Controlled Trial. PLoS One. 2015;**10**(6):e0127520.
Diabetes Prevention Program R G 2009	Knowler WC, Fowler SE, Hamman RF, et al. 10-year follow-up of diabetes incidence and weight loss in the Diabetes Prevention Program Outcomes Study. *Lancet* 2009; **374**(9702): 1677-86.<br>Ackermann RT, Edelstein SL, Narayan KM, et al. Changes in health state utilities with changes in body mass in the Diabetes Prevention Program. Obesity (Silver Spring). 2009;**17**(12):2176-2181.<br>Florez H, Pan Q, Ackermann RT, et al. Impact of lifestyle intervention and metformin on health-related quality of life: the diabetes prevention program randomized trial. J Gen Intern Med. 2012;**27**(12):1594-1601.
Fernandez-Ruiz 2018	Fernández-Ruiz VE, Armero-Barranco D, Paniagua-Urbano JA, Sole-Agusti M, Ruiz-Sánchez A, Gómez-Marín J. Short-medium-long-term efficacy of interdisciplinary intervention against overweight and obesity: Randomized controlled clinical trial. *Int J Nurs Prac* 2018; **24**(6): e12690.<br>Fernández-Ruiz VE, Paniagua-Urbano JA, Solé-Agustí M, Ruiz-Sánchez A, Gómez-Marín J, Armero-Barranco D. Impact of the I^2AO^2 interdisciplinary program led by nursing on psychological comorbidity and quality of life: Randomized controlled clinical trial. *Arch Psychiatr Nurs*. 2018;**32**(2):268-277.
Foley 2016	Foley P, Steinberg D, Levine E, et al. Track: A randomized controlled trial of a digital health obesity treatment intervention for medically vulnerable primary care patients. *Contemp Clin Trials* 2016; **48**: 12-20.
Forman 2016	Forman EM, Butryn ML, Manasse SM, et al. Acceptance-based versus standard behavioral treatment for obesity: Results from the mind your health randomized controlled trial. *Obesity (Silver Spring)* 2016; **24**(10): 2050-6.
Foster-Schubert 2012	Foster-Schubert KE, Alfano CM, Duggan CR, et al. Effect of diet and exercise, alone or combined, on weight and body composition in overweight-to-obese postmenopausal women. *Obesity (Silver Spring)* 2012; **20**(8): 1628-38.<br>Imayama I, Alfano CM, Kong A, et al. Dietary weight loss and exercise interventions effects on quality of life in overweight/obese postmenopausal women: a randomized controlled trial. *Int J Behav Nutr Phys Act*. 2011;**8**:118.
Freitas 2017	Freitas PD, Ferreira PG, Silva AG, et al. The Role of Exercise in a Weight-Loss Program on Clinical Control in Obese Adults with Asthma. A Randomized Controlled Trial. *Am J Respir Crit Care Med* 2017; **195**(1): 32-42.
Goodwin 2014	Goodwin PJ, Segal RJ, Vallis M, et al. Randomized trial of a telephone-based weight loss intervention in postmenopausal women with breast cancer receiving letrozole: the LISA trial. *J Clin Oncol* 2014; **32**(21): 2231-9.
Grilo 2011	Grilo CM, Masheb RM, Wilson GT, Gueorguieva R, White MA. Cognitive-behavioral therapy, behavioral weight loss, and sequential treatment for obese patients with binge-eating disorder: a randomized controlled trial. *J Consult Clin Psychol* 2011; **79**(5): 675-85.
Grilo 2014	Grilo CM, Masheb RM, White MA, et al. Treatment of binge eating disorder in racially and ethnically diverse obese patients in primary care: randomized placebo-controlled clinical trial of self-help and medication. *Behav Res Ther* 2014; **58**: 1-9.

Hunt 2014	Hunt K, Wyke S, Gray CM, et al. A gender-sensitised weight loss and healthy living programme for overweight and obese men delivered by Scottish Premier League football clubs (FFIT): a pragmatic randomised controlled trial. *Lancet* 2014; **383**(9924): 1211-21.
Huseinovic 2016	Huseinovic E, Bertz F, Leu Agelii M, Hellebö Johansson E, Winkvist A, Brekke HK. Effectiveness of a weight loss intervention in postpartum women: results from a randomized controlled trial in primary health care. *Am J Clin Nutr* 2016; **104**(2): 362-70.
Jackson 2018	Jackson JB, Pietrabissa G, Rossi A, Manzoni GM, Castelnuovo G. Brief strategic therapy and cognitive behavioral therapy for women with binge eating disorder and comorbid obesity: A randomized clinical trial one-year follow-up. *J Consult Clin Psychol* 2018; **86**(8): 688-701.
Katzer 2008	Katzer L, Bradshaw AJ, Horwath CC, Gray AR, O'Brien S, Joyce J. Evaluation of a "nondieting" stress reduction program for overweight women: a randomized trial. *Am J Health Promot* 2008; **22**(4): 264-74.
Mensinger 2016	Mensinger JL, Calogero RM, Stranges S, Tylka TL. A weight-neutral versus weight-loss approach for health promotion in women with high BMI: A randomized-controlled trial. *Appetite* 2016; **105**: 364-74.<br>Mensinger JL, Calogero RM, Tylka TL. Internalized weight stigma moderates eating behavior outcomes in women with high BMI participating in a healthy living program. Appetite. 2016;**102**:32-43.
Messier 2013	Messier SP, Mihalko SL, Legault C, et al. Effects of intensive diet and exercise on knee joint loads, inflammation, and clinical outcomes among overweight and obese adults with knee osteoarthritis: the IDEA randomized clinical trial. *JAMA* 2013; **310**(12): 1263-73.
Munsch 2007	Munsch S, Biedert E, Meyer A, et al. A randomized comparison of cognitive behavioral therapy and behavioral weight loss treatment for overweight individuals with binge eating disorder. *The Int J Eat Disord* 2007; **40**(2): 102-13.
Ng 2015	Ng SSS, Chan RSM, Woo J, et al. A Randomized Controlled Study to Examine the Effect of a Lifestyle Modification Program in OSA. *Chest* 2015; **148**(5): 1193-203.
Ramirez 2001	Ramirez EM, Rosen JC. A comparison of weight control and weight control plus body image therapy for obese men and women. *J Consult Clin Psychol* 2001; **69**(3): 440-6.
Schubel 2016	Schübel R, Graf ME, Nattenmüller J, et al. The effects of intermittent calorie restriction on metabolic health: Rationale and study design of the HELENA Trial. *Contemp Clin Trials* 2016; **51**: 28-33.
Silva 2010	Silva MN, Vieira PN, Coutinho SR, et al. Using self-determination theory to promote physical activity and weight control: a randomized controlled trial in women. *J Behav Med* 2010; **33**(2): 110-22.<br>Teixeira PJ, Silva MN, Coutinho SR, et al. Mediators of weight loss and weight loss maintenance in middle-aged women. *Obesity (Silver Spring).* 2010;**18**(4):725-735.
Snel 2012	Snel M, Sleddering MA, Vd Peijl ID, et al. Quality of life in type 2 diabetes mellitus after a very low calorie diet and exercise. *Eur J Intern Med* 2012; **23**(2): 143-9.
Tapsell 2017	Tapsell LC, Lonergan M, Batterham MJ, et al. Effect of interdisciplinary care on weight loss: a randomised controlled trial. *BMJ open* 2017; **7**(7): e014533.
The Look AHEAD Research Group 2010	Wing RR. Long-term effects of a lifestyle intervention on weight and cardiovascular risk factors in individuals with type 2 diabetes mellitus: four-year results of the Look AHEAD trial. *Arch Intern Med* 2010; **170**(17): 1566-75.
vanWier 2011	van Wier MF, Dekkers JC, Hendriksen IJ, et al. Effectiveness of phone and e-mail lifestyle counseling for long term weight control among overweight employees. *J Occup Environ Med* 2011; **53**(6): 680-6.
vonGruenigen 2008	von Gruenigen VE, Courneya KS, Gibbons HE, Kavanagh MB, Waggoner SE, Lerner E. Feasibility and effectiveness of a lifestyle intervention program in obese endometrial cancer patients: a randomized trial. *Gynecol Oncol* 2008; **109**(1): 19-26.<br>von Gruenigen VE, Gibbons HE, Kavanagh MB, Janata JW, Lerner E, Courneya KS. A randomized trial of a lifestyle intervention in obese endometrial cancer survivors: quality of life outcomes and mediators of behavior change. *Health Qual Life Outcomes*. 2009;**7**:17.
Wadden 1986	Wadden TA, Stunkard AJ. Controlled trial of very low calorie diet, behavior therapy, and their combination in the treatment of obesity. *J Consult Clin Psychol* 1986; **54**(4): 482-8.

Zwickert 2016	Zwickert K, Rieger E, Swinbourne J, et al. High or low intensity text-messaging combined with group treatment equally promote weight loss maintenance in obese adults. *Obes Res Clin Pract* 2016; **10**(6): 680-91.

Table S2. Risk of bias of included studies

Study ID		Random sequence generation<br>(Selection bias)	Allocation concealment<br>(Selection bias)	Blinding of outcome assessment<br>(Detection bias)	Incomplete outcome data<br>(Attrition bias)	Other bias
**1**	**Ames 2005**	UNCLEAR	UNCLEAR	UNCLEAR	HIGH	
	Assessment justification:	“Women who met the eligibility criteria were stratified based on a median split of BMI and were subsequently randomized to one of two treatment conditions.”<br><br>No further information given.	NS	NS	“The high rate of attrition during the Phase I run-in period represents a limitation of this study.”<br><br>“Following screening, 80 women met the eligibility criteria for randomization; 67 enrolled in the study and attended the first week of treatment.”<br>28 participants completed the Phase I run-in period and entered the Phase II experimental stage. 26 of the 28 participants who started Phase II of the program completed participation through Phase III.	
**2**	**Andersen 1999**	UNCLEAR	UNCLEAR	LOW	LOW	
	Assessment justification:	“Participants were randomly assigned to 1 of the 2 conditions described above.”<br>No further information given.	NS	Weight objectively measured.<br><br>Where a subjective component potentially existed (e.g. measurement of aerobic fitness), tester was blinded.	40 randomized<br>38 completed 16-week follow-up;<br>33 completed 68-week follow-up.	

**3**	**Annesi 2016**	UNCLEAR	UNCLEAR	LOW	LOW	
	Assessment justification:	NS	NS	Weight objectively measured.	"Attrition from initial study acceptance to actual treatment participation was minimal at 7% and also did not significantly differ by group."	
**4**	**Annesi 2017**	UNCLEAR	UNCLEAR	LOW	LOW	
	Assessment justification:	"To avoid cross-contamination of participants and instructors, randomisation to either the experimental (n=53) or comparison (n=54) condition was by the participating community wellness centres (3 for each condition)."<br>Unclear if cluster randomised.<br>No further information given.	To minimise expectation and cross-contamination effects, wellness leaders were trained in only 1 of the protocols by study staff and blinded to study goals.<br>No further information given.	Weight objectively measured.	"Because the requirement of data being missing at random (no systematic bias) was present, the expectation-maximisation algorithm was used for the 12% of cases necessitating imputation within the present intention-to-treat format."<br>Indicates 12% drop out, so 94/107 completed	
**5**	**Appel 2011**	LOW	LOW	LOW	LOW	
	Assessment justification:	"Randomization was stratified according to sex and was generated in blocks of 3 and 6 with the use of a Web-based program."	Web-based program.	"Participants were asked to make in-person follow-up visits 6, 12, and 24 months after randomization. At each of these visits, weight was measured on a high-quality, calibrated digital scale, with the participant wearing light, indoor clothes and no shoes."	6-month follow-up: Control: 113/138*100= 81.9%;<br>Remote: 129/139*100 = 92.8%;<br>In-person: 124/138*100 = 89.9%<br><br>12-month follow-up: Control: 108/138*100= 78.3%;<br>Remote: 124/139*100 = 89.2%;<br>In-person: 123/138*100 = 89.1%<br><br>24-month follow-up : Control: 129/138*100= 93.5%;<br>Remote: 132/139*100 = 95%;<br>In-person: 133/138*100 = 96.4%	
**6**	**Ard 2004**	LOW	LOW	LOW	LOW	

	Assessment justification:	Randomization assignments were made centrally by a computer program. Assignments were stratified by clinic and hypertension status; the randomization block size was 24.	Randomization assignments were made centrally by a computer program.	Weight was measured using a calibrated scale.	Less than 25% attrition at 6-month and 18-months follow-up.	
**7**	**Ard 2018**	LOW	LOW	LOW	LOW	
	Assessment justification:	The statistician generated blocked random assignments using a computer-based algorithm, stratified by age category (65–74, 75+), sex, and race.	Allocations were concealed in sealed envelopes that were opened by a research assistant at the time of randomization.	Body weight was measured in light clothing on calibrated electronic scales to the nearest 0.1 pound and converted to kilograms.	Less than 50% attrition.	
**8**	**Ash 2006**	LOW	UNCLEAR	LOW	HIGH	
	Assessment justification:	Randomised by the project manager, using a random number table, into one of three intervention groups at one of two hospital sites. The allocation ratio for the two hospital sites (public and private) was 2:1 due to available resources for implementing the intervention.	NS	Weight objectively measured.	BO = 20/54 complete data 37%<br>IDT = 44/65 complete data 66.7%<br>FBI = 26/57 complete data 45.6%<br>Significant between group difference in drop out and people who dropped out had significantly higher baseline BMI. At 12 months 24 BO, 49 IDT and 29 FBI had weight measurements.	
**9**	**Bacon 2002**	UNCLEAR	UNCLEAR	LOW	LOW	
	Assessment justification:	“To ensure balance in the treatment groups, the enrolled subjects (n = 78) were divided into BMI quartiles, and high/ low sets for dietary restraint, 34 degrees of flexible and	NS	Weight objectively measured.<br>Blood pressure was assessed in duplicate using the oscillometric	52-weeks:<br>Diet group: 23/39 completed testing;	

		rigid control of eating, 35 age, and self-reported activity level. The subjects in these subgroups were then randomly assigned to one of two treatment groups.”		technique. Fasting blood samples were analyzed for blood lipids (total cholesterol, low-density lipoprotein [LDL] cholesterol, and high-density lipoprotein [HDL] cholesterol).	HAES group: 34 attended (29 completed testing)/36	
**10**	**Barnes 2017**	UNCLEAR	UNCLEAR	LOW	LOW	
	Assessment justification:	Participants were randomly assigned, stratified by BED<br>diagnosis, to one of three conditions.<br><br>No further information given.	NS	Weight objectively measured.	Less than 50% attrition at 12-month follow-up.	
**11**	**Bennett 2012**	LOW	UNCLEAR	LOW	LOW	
	Assessment justification:	Participants were randomized to treatment arm using computer-generated allocations, blocked by clinic and sex.	NS	Weight objectively measured.	24-months:<br>Usual care: 166/185<br>Intervention: 148/180	
**12**	**Bennett 2013**	LOW	UNCLEAR	LOW	LOW	
	Assessment justification:	A computer-generated randomization algorithm to allocate participants equally (1:1) across the 2 treatment arms (intervention and usual care); those in the intervention arm were further randomized to 1 of 2 interventionists.	NS	Weight objectively measured.<br>Secondary measures included waist circumference, blood pressure, and fasting glucose, triglyceride, and cholesterol level.	Usual care: 90/97<br>Intervention: 86/97	
**13**	**Beutel 2006**	UNCLEAR	UNCLEAR	LOW	LOW	
	Assessment justification:	“These 396 patients were externally randomized (random digits) either to BT or to PD. A minority of patients who were directly referred to a specific setting (usually behavioral) were excluded from randomization.”	NS	Upon intake, BMI was checked based on the current weight and height.<br>Follow-up GPs assessed blood pressure, weight and laboratory data.	Behaviour treatment: 154/175 at approximately 7-weeks;<br>At 12-months:<br>97/175<br>Psychdynamic treatment: 168/179; 97/179 at 1 year.	
**14**	**Burke 2015**	LOW	UNCLEAR	LOW	LOW	
	Assessment justification:	Randomization used the minimization method. Treatment assignments were determined considering gender and ethnicity (White vs. non-White) to ensure balance across the treatment groups.	NS	"Data were collected at the research center by trained staff using<br>standardized procedures and questionnaires. Equipment was	79.2% in SBT arm and 81% in SBT+SE arm completed 18-month assessment.	

				standardized and routinely calibrated"		
**15**	**Cleo 2018**	LOW	UNCLEAR	LOW	LOW	
	Assessment justification:	"Computer-generated randomization occurred after baseline assessment to allocate participants to either: TTT, DSD, or WL control (allocation ratio 1:1:1). We used minimization stratified on BMI categories (overweight, obese class I, II, III); age (18–32, 33–47, 48–62, 63–75 years); and gender."	NS	Weight objectively measured.	At 12-months:<br>21/25 (84%)<br>22/25 (88%)	
**16**	**Conroy 2015**	UNCLEAR	LOW	UNCLEAR	LOW	
	Assessment justification:	"…randomization occurred in a 1:1 allocation. Each woman was allowed to draw a sealed envelope that contained a designation assignment, either interventionist-led (IL) or self-guided (SG)."	Sealed envelopes.	Weight was measured by a trained staff member in clinic using a standard balance beam scale (SECA Medichoice) and following a written protocol.<br>" For the 12-month followup, 62 (74 %) of 84 participating women had an in-person assessment (with study-measured weight), with the remainder of the outcomes assessed by phone." Breakdown by group not clear	"Follow-up was better in the IL group (90 % at 3 months and 96 % at 12 months) than in the SG group (63 % at 3 months and 76 % at 12 months), but otherwise did not differ by other participant characteristics."	
**17**	**Damschroder 2014**	LOW	UNCLEAR	LOW	LOW	
	Assessment justification:	"…a biostatistician provided block randomized assignments (by medical center and two BMI categories [o35 or Z35] to ensure balance between groups) using random permutated blocks constructed by Stata's *ralloc* command; block sizes ranged from 3 to 9…"	"Investigators were blind to assignments until baseline assessments were complete."	Anthropometric measures (height, weight, and waist circumference); blood pressure; and self-reported measures including a Food Frequency Questionnaire; EuroQoL-5D utility assessment (with level of painsubscale); Satisfaction with Life Scale; demographic characteristics; laboratory testing for cholesterol and glucose metabolism; and a 6-	Move:<br>3mth: 115/159;<br>12mth: 119/159<br>Aspire phone:<br>3mth: 131/162;<br>12mth: 120/162<br>Aspire group:<br>3mth: 127/160;<br>12mth: 122/160;<br>Follow up 332/481 consented to long term follow up.	

				minute walk test were collected in baseline, 3-month, and 12-month assessments.	Move:<br>18mth: 92/112;<br>24mth: 90/112<br>Aspire phone:<br>18mth: 95/105;<br>24mth: 92/105<br>Aspire group:<br>18mth: 102/115;<br>24mth: 104/115	
**18**	**Daubenmier 2016**	LOW	LOW	LOW	LOW	
	Assessment justification:	"A computer-generated random allocation sequence using random block sizes of four to eight was programmed by a database manager not involved in enrollment."	"No other staff had access to the randomization sequence. The project director (PM) accessed the allocation sequence using a programmed database that could not be altered once randomized condition was revealed."	"Weight was measured to the nearest 0.1 kg on a calibrated digital scale (Wheelchair Scale 6002, Scale-Tronix, Carol Stream IL), with participants wearing a hospital gown. The same scale was used for measurements throughout the study."	At 18-months follow-up 81% of participants from the mindfulness group and 71% from the control group were followed up.	
**19**	**de Zwaan 2017**	UNCLEAR	LOW	LOW	LOW	
	Assessment justification:	“Individuals who met the respective inclusion criteria and who gave their written informed consent to participate were randomized. There were no stratification criteria.”	“To ensure the concealment of allocation, randomization was performed centrally by fax by the Coordination Center for Clinical Trials (KKS) in Marburg. Eligibility assessment, obtaining informed consents, and enrolling the participants in the study were done at the respective study centers.”	Treatment and assessment were separated. Therapists and coaches are not involved in assessing treatment outcome, and assessors are not allowed to hold treatment sessions or write e-mails. The statistician who will conduct the statistical analyses was not involved in randomization. Treatment allocation is not disclosed to the statistician until all data checks are completed.	Treatment attrition and study dropout during treatment were low.<br><br>At intervention end: GSH-I: 77/89*100 = 86.5%;<br>CBT: 85/89*100 =95.5%<br><br>6-month follow-up after intervention end: GSH-I: 70/89*100 = 79%;<br>CBT: 80/89*100 =89.9%<br><br>12-month follow-up: GSH-I: 58/89*100 = 65%;<br>CBT: 58/89*100 =65%	
**20**	**deRoon 2017**	LOW	UNCLEAR	HIGH	LOW	
	Assessment justification:	“After baseline measurements, women were stratified for municipality randomized by computer.”	NS	“At baseline and end of study body weight was measured using an identical balance scale, but at follow-up, body weight	Anthropometrics:<br>At 16-weeks:<br>control 45/48;<br>diet 94/97;	

				was self-reported by the participants.”	exercise 93/98<br><br>At 12-months:<br>control: NS;<br>diet: 78/97;<br>exercise: 77/98.	
**21**	**Diabetes Prevention Program R G 2009**	LOW	LOW	LOW	LOW	HIGH
	Assessment justification:	“The randomization was done centrally by computer…”<br>Random treatment assignments were stratified according to clinical center and were generated by the coordinating center through computer linkup to the field center at<br>time of randomization. Therefore, assignment was unknown until randomization. Assignments to metformin and placebo were double-blinded.	“…assignments to the lifestyle group were blinded until randomization, while assignments to the medication groups were blinded until the end of the study.”	Lifestyle intervention participants were weighed privately at the start of every individual session and were encouraged to weigh themselves at home daily or a minimum of once per week.	Placebo<br>yr. 1 - 1027/1082;<br>yr. 2 - 1015/1082;<br>yr. 3 - 975/1082.<br>Bridge period DPPOS - 1085 eligible, 935 enrolled. DPPOS<br>yr. 1 882/935;<br>yr. 2 874/935;<br>yr. 3 844/935;<br>yr. 4 - 827/935;<br>yr. 5 - 846/935;<br>yr. 6 808/935;<br>yr. 7 - 789/935;<br>yr. 8 766/935;<br>yr 9. 760/935;<br>yr. 10 - 763/935;<br>yr. 11- 769/935.<br>Lifestyle<br>yr. 1 - 1026/1079;<br>yr. 2 - 1001/1079;<br>yr. 3 - 972/1079.<br>Bridge period DPPOS - 1068 eligible, 914 enrolled. DPPOS<br>yr. 1 855/914;<br>yr. 2 827/914;<br>yr. 3 816/914;<br>yr. 4 - 810/914;<br>yr. 5 - 824/914;<br>yr. 6 783/914;<br>yr. 7 - 763/914;<br>yr. 8 757/914;<br>yr. 9 738/914;<br>yr. 10 - 725/914;<br>yr. 11.- 738/914.	0-3 years LOW<br>From year 4 HIGH<br>DPP was a 3-year randomized clinical trial followed by open-label modified intervention follow-up.

**22**	**Fernandez-Ruiz 2018**	UNCLEAR	UNCLEAR	LOW	LOW	
	Assessment justification:	Randomisation was performed using a simple table of numbers: 37 patients in the control group and 37 in the experimental group.<br>“A random allocation sequence was generated by a member of the scientific staff through extraction of successive numbered balls from an opaque container, alternating between the experimental and the control group'	Refer to ‘Random sequence generation (selection bias)’.	“The efficacy of the intervention was evaluated through anthropometric (body mass index, weight, different parameters, and skinfolds, as stated in Section 2) and cardiovascular measures taken before, during, and after intervention.'<br>'Anthropometric and cardiovascular measures were taken at the pretest stage, every 6 months during the programme, and 1 year after it finished.”	No loss to follow up reported.	
**23**	**Foley 2016**	LOW	UNCLEAR	LOW	LOW	
	Assessment justification:	“Randomization occurred at the baseline visit, using a computer-based algorithm. The randomization algorithm allocated participants equally (1:1) across treatment arms, after accounting for CHC, gender and ethnicity (Hispanic vs. non-Hispanic) in order to ensure the equal representation of these characteristics across arms.”	NS	Weight measured objectively.	Less than 50% attrition at 12-month follow-up.	
**24**	**Forman 2016**	UNCLEAR	UNCLEAR	LOW	LOW	

	Assessment justification:	“Once enrolled, participants were randomly assigned to SBT (n 5 90) or ABT (n 5 100). Randomization was stratified by gender and ethnicity.”	NS	Weight measured objectively.	End of treatment: SBT 70/90; ABT 79/100<br>“Treatment attendance (with inclusion of makeup sessions) was in excess of 84% of expected sessions, and there were no differences between the two treatments in terms of the average number of sessions attended (MABT = 21.26 +/- 5.85, MSBT = 20.88 +/- 5.46; t(189) =-0.46, P = 0.65). Overall, 84.2% of the ABT participants and 85.6% of SBT participants attended the majority (i.e., 18 or more) of the 25 scheduled groups (X2 5 0.07, df = 1, P = 0.79). A total of 142 participants (74%) completed the mid-treatment assessment and 149 participants (78%) completed the post-treatment assessment.”<br><br>Retention rate:<br>24-month follow-up: SBT: 65/90*100 = 72.2%; ABT: 78/100*100 = 78%<br>36-month follow-up: SBT: 61/90*100 = 67.7%; ABT: 74/100*100 = 74%	
**25**	**Foster-Schubert 2012**	LOW	LOW	LOW	LOW	UNCLEAR

	Assessment justification:	"The random assignment was generated by a computerized program, stratified according to BMI (<30 kg/m2 or ≥30 kg/m2) and participants' self-reported race/ethnicity (non-Hispanic white, black, or other). In addition, to achieve a proportionally smaller number of women assigned to the control group, a permuted blocks randomization with blocks of four was used, wherein the control assignment was randomly eliminated from each block with a probability of ~1 in 4." Blocked-randomisation. (Permuted-block randomization (ratio 0.75 : 1 : 1 : 1) to assign a proportionally smaller number of women to the control group.)	Central computerised allocation.	Weight measured objectively.	91% followed up at 12m overall: 92% D+E, 89% D only, 91% E only, 92% usual care. 2 unavoidable losses (<1%); 8% missing; 1% medical reason.	Control group received intervention at 12m, unclear if they knew in advance.
**26**	**Freitas 2017**	LOW	LOW	HIGH	UNCLEAR	
	Assessment justification:	"Randomization schedule was computer-generated and implemented by an investigator blinded to the recruitment, evaluation and treatment of the participants."	" Each patient's allocation was concealed using sequential numbering and then sealed and placed in opaque envelopes..."	"The nutritionist and psychologist, as well as the outcome assessors, were blinded throughout the duration of the study."<br><br>"The long-term effect (6 and 12 mo after randomization) was evaluated by obtaining body weight from patients' medical records."	No information given on n followed up at 6- and 12-months.	
**27**	**Goodwin 2014**	LOW	UNCLEAR	LOW	LOW	
	Assessment justification:	"Random assignment was performed centrally by the Ontario Clinical Oncology Group, and a computer-generated block randomization scheme with blocks of various size was used."	NS	Weight objectively measured.	"Six patients in the mail-based intervention and seven in the LI arm did not complete the 24-month intervention period because of a primary outcome event (new disease, metastases, death); of the remaining patients, 14 (8.7%) and 16 (9.9%), respectively, withdrew (including patients who transferred care, those who were lost to follow-up, or those with noncompliance). Month-24 weight measurements were	

					available from 264 (90.1%) of 293 participants still on the study."	
**28**	**Grilo 2011**	LOW	UNCLEAR	LOW	LOW	
	Assessment justification:	"Randomization to treatment was performed without any restriction or stratification, using a computer-generated sequence."	"Randomization was determined after formal acceptance into the study and completion of all assessments. Randomization assignment was kept from participants until the start of treatment."	"Weight and height were measured at baseline and again immediately prior to beginning treatment using a trade-legal medical balance-beam scale. Weight was measured bi-weekly throughout treatment, at post-treatment, and at 6- and 12-month follow-ups."	Retention rate:<br>Post-intervention:<br>CBT: 75.6%;<br>BWL: 68.9%;<br>CBT+BWL: 60.0%<br><br>6-Months:<br>CBT: 82.2%;<br>BWL: 86.7%;<br>CBT+BWL: 85.7%<br><br>12-Months:<br>CBT: 82.2%;<br>BWL: 82.2%;<br>CBT+BWL: 71.4%<br><br>"Completion rates, which did not differ statistically, were: 76% (N=34) for CBT, 69% (N=31) for BWL, and 60% (N=21) for CBT+BWL. Follow-up (6-and 12-month) assessments were obtained for over 80% of patients (Figure 1)."	
**29**	**Grilo 2014**	LOW	LOW	LOW	LOW	
	Assessment justification:	"Randomization to treatment assignment occurred in the exact order following completion of all assessments and medical approval and was performed independently from the investigators by a research-pharmacist at a separate Yale facility using a computer-generated schedule generated by a biostatistician. Participants were randomly assigned with stratification by BED status."	"Randomization to treatment assignment occurred in the exact order following completion of all assessments and medical approval and was performed independently from the investigators by a research-pharmacist at a separate Yale facility using a computer-generated schedule generated by a biostatistician. Participants were randomly assigned with stratification by BED status."	"The assessments were performed independently by doctoral research evaluators at our research clinic who were blinded to both the medication status and to whether participants received the shCBT."	"Post-treatment assessments were obtained for 84% of patients and follow-up assessments were obtained for 83% of patients at the 6-month follow-up and for 86% of patients at the 12-month follow-up."<br><br>Retention rates:<br>Post-treatment: Placebo: 20/27*100=74.1%;<br>Placebo/CBTsh: 22/25*100 =	

					88%<br><br>6-Months: Placebo: 22/27*100=81.4%; Placebo/CBTsh: 23/25*100 = 92%<br><br>12-Months: Placebo: 19/27*100=70.4%; Placebo/CBTsh: 21/25*100 = 84%	
**30**	**Hunt 2014**	LOW	LOW	LOW	LOW	HIGH
	Assessment justification:	“After baseline measurement, the randomisation sequence was generated by the Tayside Clinical Trials Unit (TCTU) statistician (with no day to day role in the study at this point) with SAS (version 9.2), blocked (block size between two and nine dependent on how many participants were recruited at a club), and stratified by club.”	“The allocation sequence was sent in a password protected file to a database manager (not part of the research team) who assigned individuals to each group.”	Weight measured objectively.	Comparison (control): 347/374 at 12-weeks; 355/374 at 12-months.<br>Intervention:<br>330/374 at 12-weeks; 333/374 at 12-months	12 month wait-list control.
**31**	**Huseinovic 2016**	LOW	LOW	LOW	LOW	
	Assessment justification:	"...simple randomization procedure that used numbered and sealed envelopes generated through a random number table prepared by the project coordinator."	Refer to ‘Random sequence generation (selection bias)’.	"All study measures and administration of intervention were completed by 2 dietitians at the primary health care clinics. Blinding of the study dietitians was not possible..."	D Group:<br>Baseline n=54;<br>12-wk: n=47;<br>1-year: n=44<br>C Group:<br>Baseline n=56;<br>12-wk n=53;<br>1-year: n=45	
**32**	**Jackson 2018**	LOW	UNCLEAR	LOW	LOW	
	Assessment justification:	"The randomization scheme have been generated by using the Web site Randomization.com (www. randomization.com)."	NS	" Assessments were conducted by inpatient clinic staff and graduate psychology trainees who were blinded to participant	"No data were missing for any of the participants on any of the outcome measures at any of the measurement points."	

				treatment condition assignment."		
**33**	**Katzer 2008**	UNCLEAR	UNCLEAR	LOW	LOW	
	Assessment justification:	"Randomization was stratified by age and BMI and performed independently by a statistician."	NS	Weight objectively measured.	"By the end of the initial 10-week intervention, 53 participants had withdrawn from the study (24%), and an additional 28 participants (overall dropout rate=37%) had withdrawn by the 12-month follow-up. Dropout rates were similar in the three treatments (data not shown)."	
**34**	**Mensinger 2016**	LOW	LOW	LOW	HIGH	
	Assessment justification:	"This study was a 1:1 parallel-group randomized design comparing the effectiveness of two 6-month group-based "healthy living programs" (weight-neutral or weight-loss).<br>Folded index cards containing program assignments from a computer-generated randomization scheme were placed into sealed and sequentially numbered opaque envelopes."	"Folded index cards containing program assignments from a computer-generated randomization scheme were placed into sealed and sequentially numbered opaque envelopes. Upon completion of the baseline assessments where informed consent was obtained, participants were given an envelope containing a welcome letter with their assignment and instructions."	Weight measured objectively.<br>Waist and hip circumference was measured to the nearest quarter inch with a flexible tape measure on bare skin.<br>Venous blood samples were drawn after an overnight fast in order to obtain glucose levels and lipid panels (total cholesterol, LDL-C, HDL-C, total cholesterol-HDL ratio, and triglycerides). We followed standardized methods established by the National High Blood Pressure Education Program and averaged two blood pressure (BP) readings using a Welch Allyn cuff with an aneroid sphygmomanometer.	Weight neutral -<br>39/40 at 6mths (LOW); 19/40 at 24mths (HIGH)<br>weight loss -<br>33/40 at 6mths (LOW); 21/40 at 6mths (LOW).<br><br>Attrition rate at 6-month for the weight loss group > 25%, change from low to high.	
**35**	**Messier 2013**	LOW	UNCLEAR	LOW	LOW	
	Assessment justification:	"A stratified-block randomization method was used to assign all eligible persons to 1 of the 3 intervention groups, stratified by BMI and sex."	NS	Weight, height, and BMI were obtained at baseline, 6months, and 18months using standard techniques.	"Of the 454 participants, 399 (88%) completed the study (returned for 18- month follow-up). Retention did not differ significantly among the groups (E, 89%; D, 85%; D+E, 89%)..."	

**36**	**Munsch 2007**	LOW	UNCLEAR	LOW	LOW	
	Assessment justification:	"Following diagnostic interviews, patients were randomized according to a permuted block design."	NS	"Weight and height were measured on a Seca electronic balance scale (Seca, Vogel þ Halke, Germany) and by a stadiometer."	"Twenty-two participants (27.5%) dropped out during treatment: 13 (29.5%) in CBT and 9 (25.0%) in BWLT. During follow-up 3 participants withdrew from CBT and 4 from BWLT. There were no significant differences in dropout rates between the two treatment conditions between baseline and 12- month follow-up."<br>"A final follow-up measurement took place on average 307.5 weeks (median ¼ 314.5, SD ¼ 46.9, MIN ¼ 217, MAX ¼ 373), i.e. c. 6 years, after the active treatment had ended. To this end, all patients attending at least one session of active treatment were again contacted. Of the initial 44 patients allocated to the CBT condition andthe 36 patients allocated to the BWLT condition, 26 (59%) in the CBT, and 26 (72%) in the BWLT took part in the 6-year follow-up assessments."	
**37**	**Ng 2015**	LOW	UNCLEAR	LOW	LOW	
	Assessment justification:	"Recruited patients were randomized in 1:1 ratio to participate in the LMP or usual care through the use of a computer-generated list of random numbers."	NS	"Anthropometric measurements, ESS, and laboratory tests, which included liver and renal function, fasting glucose, and lipids, were performed at baseline, 4 months, and 12 months."	"Sixteen participants in the intervention group were excluded after randomization, as six had never attended dietician visits, seven attended fewer than four dietician visits, and three maintained their high-energy and -fat food intake. There were six subjects in the control group lost to follow-up."<br><br>LMP Group:	

					45/61*100 = 73.8%;<br>Control:<br>37/43*100 = 86%	
**38**	**Ramirez 2001**	UNCLEAR	UNCLEAR	UNCLEAR	LOW	
	Assessment justification:	NS	NS	A digital scale was used. "At 12m FU, there were several participants who were unavailable for weighing in the research center. In those instances, self-reported weight was taken and corrected by taking the average discrepancy between observed and self-reported weights on the three prior weightings" (unclear how many participants this applies to and which study arm)	Attrition was less than 50% for both groups at 12-month follow-up.	
**39**	**Schubel 2016**	LOW	LOW	LOW	LOW	
	Assessment justification:	"They sequentially enter the study and are randomly allocated to the three dietary programs (ICR, CCR, or HD) by RANDI2 [9], a web-based software using a block size of six. Randomization is stratified by age (<50 years/ ≥ 50 years) and sex."	Refer to 'Random sequence generation (selection bias)'.	"All outcome assessments (see Table 2) are performed by trained study personnel following standard operating procedures."	Overall, 144 participants (96.0%) completed the 12-wk intervention phase, 143 (95.3%) the 12-wk maintenance phase, and 136 (90.7%) the 26-wk follow-up phase (Figure 1). Across the entire study period of 50 wk there were 4 dropouts in the ICR (91.8%), 7 in the CCR (85.7%), and 2 (96.2%) in the Control group.	
**40**	**Silva 2010**	LOW	UNCLEAR	LOW	LOW	UNCLEAR

	Assessment justification:	"...using the random number generator function for Microsoft Excel 2007 for Windows."	NS	"Assessments included lab-measured body weight and body composition (assessed at baseline, 4 and 12 months (end of the intervention program))..." At 2 and 3 year follow-ups: "Body weight was measured twice, using an electronic scale calibrated on site and accurate to 0.1 kg (SECA, Hamburg, Germany)."	Retention rates at each follow-up (not reported for 2-year follow-up):<br><br>12-months:<br>Comparison group: 80%;<br>Intervention group: 93%;<br><br>3-year:<br>Comparison group: 80%;<br>Intervention group: 79%*<br><br>*"For the 36-month analyses reported herein, 2 women without 36-month anthropometric data were excluded, leaving a final sample of 154 women."	"A total of 258 women completed initial assessments and were randomized to intervention and comparison groups. Thirty-seven women were subsequently excluded from all analyses because they started taking medication (antidepressant, anxiolytic, and antiepileptic) susceptible to affect weight (n = 13) or because of serious chronic disease diagnosis or severe illness/injury (n = 4). Others were excluded because of pregnancy (n = 11) or because they entered menopause (n = 9). These 37 women were of similar age (P = 0.737) and BMI (P = 0.852) as the 221participants who were considered as the valid initial sample for this study."
**41**	**Snel 2012**	UNCLEAR	UNCLEAR	LOW	LOW	
	Assessment justification:	NS	NS	"...patients visited the research center after an overnight fast. Height, weight and waist circumference were measured."	"All patients completed the whole study period of 18 months, there were no dropouts from the study."	
**42**	**Tapsell 2017**	LOW	LOW	LOW	HIGH	
	Assessment justification:	"Randomisation was conducted after the second screen for eligibility and performed remotely by an investigator unrelated to the clinic using a computer generated randomisation sequence (STATA V12, StataCorp LP, College Station, TX). The randomisation was stratified according to sex and BMI (low BMI: ≤30 and high BMI: >30). Randomisation was performed in randomly allocated blocks of 3, 6 or 9. "	"The randomisation list was provided to the study team who added eligible participants sequentially for each of the strata. The randomisation and participant database was only accessible by the HealthTrack study co-ordinator and administrator for security."	"Body weight (kg) was measured in an upright position in minimal clothing and without shoes using scales with a bio-electrical impedance component to also estimate body fat (%) (Tanita TBF-662, Wedderburn Pty Ltd., Ingleburn, NSW, Australia)."	"The intensive phase was completed by 298 participants (withdrawal rate 18%) and the 12 months follow-up by n=178 participants (withdrawal rate 39%)."<br><br>Total sample withdrawal rate at 12 months = 178/377*100 = 47%<br>12-month follow-up rate per group: Control: 61/126*100 = 48%;<br>Intervention: 45/120*100 =	

					36%; Intervention plus walnut: 72/126*100 = 57%	
**43**	**The Look AHEAD Research Group 2010**	LOW	LOW	LOW	LOW	HIGH
	Assessment justification:	"Eligible participants are randomly assigned to either diabetes support and education or lifestyle intervention using a web-based data management system that verifies eligibility. Randomization is stratified by clinical center and blocked with random block sizes." (protocol)	"Eligible participants are randomly assigned to either diabetes support and education or lifestyle intervention using a web-based data management system that verifies eligibility. Randomization is stratified by clinical center and blocked with random block sizes." (protocol)	"Weight was measured in duplicate on a digital scale."	Retention rate:<br>Year 1:<br>DSE: 95.7%; ILI: 97.1%;<br>Year 2:<br>DSE: 93.5%; ILI: 94.9%;<br>Year 3:<br>DSE: 93.8%; ILI: 94.0%;<br>Year 4:<br>DSE: 93.0%; ILI: 94.1%;<br>Year 5:<br>DSE: 92.2%; ILI: 93.3%;<br>Year 6:<br>DSE: 90.6%; ILI: 92.0%;<br>Year 7:<br>DSE: 89.3%; ILI: 90.6%;<br>Year 8:<br>DSE: 88.3%; ILI: 89.9%.	Participants in the intervention arm who, during the first 6 months, failed to lose 10% of their initial weight were offered a weight loss medication (orlistat). Those who lost <5% were encouraged by their lifestyle counselor to try pharmacotherapy, whereas those who lost 5.0% to 9.9% were informed of medication and could receive it on request. Medication was not offered to individuals who lost greater than or equal to 10% of initial weight and maintain the loss. 523 out of 2570 participants in the ILI study arm took Orlistat as part of the intervention.
**44**	**vanWier 2011**	LOW	LOW	LOW	LOW	

	Assessment justification:	After baseline measurements, the employee was randomised to one of the three study groups and either to a group receiving basic weight measurements (80% of each study group) or to a group receiving additional measurements (20% of each study group). This two-step randomisation meant that there were six groups an employee could be assigned to. Randomisation to these six groups was done by block randomisation, with each block containing 15 allocations. A computerized random number generator drew up an allocation schedule.	An administrative assistant put the group allocation in opaque sealed envelopes, numbered 1 to 1,500. These envelopes were taken to the locations of the baseline measurements and opened in the given order. The researchers were blinded for the allocation schedule, but were not blinded for allocation after randomisation.<br>The participants were, in consequence of the nature of the intervention, not blinded for allocation after randomisation. Employees were not allowed to change groups after randomisation.	At baseline 'body weight and body height were assessed by the researchers.'<br>'Body weight and body height are assessed in all participants. Body weight is measured in kg, to the nearest 0.1 kg, with a digital scale (Seca 770; Seca GmbH & Co, Hamburg, Germany). Participants are wearing light clothing and no shoes. Body height is measured in m, to the nearest 0.001 m, with a portable stadiometer (Seca 214, Leicester Height Measure; Seca GmbH & Co, Hamburg, Germany). ' LOW<br>In addition, in a questionnaire self-reported body weight is assessed. Participants are asked to weigh themselves wearing light clothing and no shoes. HIGH	At 24mths:<br>Control 266/460<br>Internet 263/464<br>Phone 263/462<br>all <50%	
**45**	**vonGruenigen 2008**	LOW	UNCLEAR	LOW	LOW	
	Assessment justification:	"Participants were randomly assigned to LI or UC. Randomization was stratified according to patient BMI (25- 39.9 versus >40 kg/m2) using a stratified blocked randomization scheme in order to achieve comparability between the study groups based on BMI…"	NS	"Participants were weighed in street clothes without shoes on a Detecto hand rail scale (model #6855) and weight was recorded to the nearest 0.1 kg."	At 12-months:<br>Control: 18/22 Intervention: 17/23	
**46**	**Wadden 1986**	UNCLEAR	UNCLEAR	LOW	LOW	
	Assessment justification:	"Subjects were stratified into three blocks based on degree overweight and were randomly assigned to one of three treatment conditions…"<br><br>No further information given.	NS	"The dependent variables were weekly measures of weight (balancebeam scale), blood pressure (Banmanometer 260 sphygmomanometer)<br>and depression, as assessed by the Beck Depression Inventory (Beck, Ward, Mendelson, Mock, & Erbaugh, 1961)."	"The following analyses are all based on the 50 out of 59 subjects<br>completing treatment. Attrition (15.3%) was spread evenly across conditions and included one pregnancy, three nondietrelated<br>illnesses, and five work/transportation conflicts. A 1-	

					year follow-up was completed on 48 of the 50 subjects finishing<br>treatment (2 subjects in the combined treatment condition could not be reached)."	
**47**	**Zwickert 2016**	LOW	UNCLEAR	LOW	HIGH	
	Assessment justification:	Participants were entered into a database sequentially and a computer-generated randomisation list was used to allocate participants to the CBT + ITS or CBT + MTS conditions.	NS	Weight measured objectively.	15-month loss-to-follow-up >50% (15/31 ITS and 14/29 MTS follow-up at 15m)<br><br>Participants who dropped out of the CBT group treatment had significantly higher baseline weight and BMI than those who continued in the trial (113.8 ± 23.3 vs. 99.6 ± 18.8 kg, p = .019, and 41.1 ± 8.3 vs.36.2 ± 5.4 kg/m2, p = .010).	
**BP:** Blood pressure; **HDL-C:** High density lipoprotein cholesterol; **Mths:** Months; **NS:** Not specified; **Wk/s**: week/s; **Yr/s:** Year;/s						

Table S3. Characteristics of included studies

Study ID	Country:	MH outcome follow-up time points (months):	Population	Study groups and number of participants randomised in primary RCT	Mental health outcome measures extracted*	Overall Risk of Bias	Additional mental health information provided by study authors[#]:
Ames 2005	USA	3, 6, 12	College women who are overweight or obese	Standard behavioural treatment = NS<br>Reformulated cognitive-behavioural treatment = NS	BDI-II; MBSRQ-AS; MBSRQ-BAS; RSES	High	
Andersen 1999	USA	4, 16	Women with obesity	Diet + Lifestyle Activity = 20<br>Diet + Aerobic Group = 20	BDI-IA	Unclear	
Annesi 2016	USA	3, 6, 12, 24	Women who are obese	Comparison treatment = 55<br>Experimental treatment = 55	Profile of Mood States Short Form scale of Total Mood Disturbance	Unclear	
Annesi 2017	USA	3, 6, 12, 24	Women with class 1 or 2 obesity (BMI ≥ 30 < 40 kg/m2).	Control comparison group = 54<br>Experimental group = 53	Profile of Mood States Short Form scale of Total Mood Disturbance	Unclear	
Appel 2011	USA	6, 24	Adults who were at least 21 years of age with obesity and had one or more cardiovascular risk factors (hypertension, hypercholesterolemia, or diabetes).	Control (Self-directed) = 138<br>Remote Support Only (N/A) = 139<br>In-Person Support = 138	SF-12 MCS; PHQ-8	Low	
Ard 2004	USA	6, 18	The target population consisted of generally healthy adults with above optimal BP including individuals with stage 1 hypertension who met Joint National Committee on Detection, Evaluation, and Treatment of High Blood Pressure (JNC-VI) criteria for at least a 6-month trial of nonpharmacological therapy.	"advice only" comparison group = 273<br>"established" behavioural intervention group = 268<br>Established + DASH Intervention Group = 269	Rand 36-item Health Survey 1; Mental health composite score	Low	
Ard 2018	USA	6, 12	General population of adults aged 65 and older who were at risk for cardiometabolic disease due to obesity and associated risk factors	Exercise Only = 54<br>Exercise + Diet Quality + Weight Maintenance = 55<br>Exercise + Diet Quality + Weight Loss = 55	SF-36 MCS version 2; IWQOL-Lite	Low	
Ash 2006	Australia	3, 12	General population and hospital referrals (one public hospital and one private hospital) overweight and obese	Control Group - Booklet only = 63<br>Individualised Dietetic Treatment = 66<br>Fat Booters Incorporated = 62	GHQ-12; SWLS	High	Y

Bacon 2002	USA	3, 6, 12	Women from the general population	Health at Every Size – control = NS<br>Diet Group – intervention = NS	BDI-IA; BIAQ; RSES	Unclear	
Barnes 2017	USA	3, 6, 15	Overweight and obese with or without binge eating	Treatment as usual (N/A) = 30<br>Nutrition - ATTENTION CONTROL = 29<br>Motivational interviewing = 30	BDI-IA	Unclear	
Bennett 2012	USA	6, 12, 18, 24	Obese patients receiving hypertensive treatment	Control, Usual care = 185<br>Be Fit, Be Well = 180	PHQ-8; EQ-5D 5L (Anxiety/depression dimension)	Unclear	Y
Bennett 2013	USA	6, 12, 18	General population	Control, usual care = 97<br>Weight gain prevention intervention = 97	PHQ-8; EQ-5D 5L (Anxiety/depression dimension)	Unclear	Y
Beutel 2006	Germany	1.5, 13.5	Patients referred for inpatient psychosomatic rehabilitation by the health and pension insurance companies on the basis of obesity plus additional psychiatric morbidity and a reduced or threatened work capacity	Behavioural therapy = 130<br>Psychodynamic treatment = 137	Global Severity Index-90 (GSI-90) based on the German version of the Symptom Checklist (SCL-90R)	Unclear	
Burke 2015	USA	6, 12, 18	General population	Standard behavioural weight loss treatment = 72<br>Self-efficacy enhancement plus standard behavioural weight loss treatment = 58	SF-36 MCS	Unclear	
Cleo 2018	Australia	3, 12, 15	General population	Wait list control (N/A) = 25<br>TTT Top Ten Tips habit formation = 25<br>DSD Do Something Different online software = 25	Eight-item wellbeing questionnaire; Depression and Anxiety each measured on a four-point ordinal scale	Unclear	
Conroy 2015	USA	3, 12	General population	Self-guided = 50<br>Interventionist led = 49	BDI; SF-12 MCS	Unclear	
Damschroder 2014	USA	3, 12	Veterans	Control, MOVE - usual care = 159<br>ASPIRE group, individual telephone counselling = 162<br>ASPIRE group, group counselling = 160	SWLS	Unclear	
Daubenmier 2016	USA	6, 12, 18	Adults with obesity	Active control intervention = 94<br>Mindfulness Intervention = 100	10-item Perceived Stress Scale	Low	
de Zwaan 2017	Germany and Switzerland	4, 10	Participants had to meet the diagnostic criteria for Binge Eating Disorder or Subsyndromal Binge Eating Disorder	Internet-based guided self-help treatment = 89<br>Cognitive Behavioural Therapy = 89	BDI-II; RSES; IWQOL-Lite; EDE: Global score	Unclear	
deRoon 2017	Netherlands	4	General population	Control = 48<br>Diet = 97<br>Exercise = 98	SF-36 MCS	High	
Diabetes Prevention Program R G 2009	USA	12	People at high risk for type 2 diabetes (impaired glucose tolerance)	Placebo = 1082<br>Metformin (N/A) = 1073	SF-36 MCS; BDI; BAI	High	

				Lifestyle = 1079			
Fernandez-Ruiz 2018	Spain	12, 24	General population (Community Care Centre population (health centre patients))	Control = 37<br>Intervention (healthy eating, exercise & CBT) = 37	SF-36 MCS; State-Trait Anxiety Questionnaire - State	Unclear	
Foley 2016	USA	6, 12	Obese (BMI: 30.0-44.9 kg/m2) community health center patients with a diagnosis of hypertension, diabetes and/or hyperlipidemia	Usual care (Control) = 175<br>Weight loss intervention = 176	PHQ-8; Global Perceived Stress Scale score (Jackson Heart study); EQ-5D 5L (Anxiety/depression dimension)	Unclear	Y
Forman 2016	USA	6, 12, 24, 48	General population	Standard Behavioural Treatment = 90<br>Acceptance-Based Treatment = 100	BDI-II	Unclear	Y
Foster-Schubert 2012	USA	12	Post-menopausal women	Control- usual care = 87<br>Calorie reduced diet = 118<br>Aerobic exercise (N/A) = 117<br>Intervention - diet and exercise = 117	Brief Symptom Inventory 18 – Depression; Brief Symptom Inventory 18 – Anxiety; Perceived Stress Scale; SF-36 MCS	Unclear	
Freitas 2017	Brazil	3	30 to 60-year-old patients with moderate/severe asthma	Weight loss program + Sham = 27<br>Weight loss program + Exercise = 28	HADS – Anxiety; HADS - Depression	High	Y
Goodwin 2014	Canada; USA	6, 12, 18, 24	Postmenopausal women diagnosed with T1-3N0-3M0 breast cancer	Mailed-based intervention = 167<br>Individual lifestyle intervention = 171	SF-36 MCS	Unclear	
Grilo 2011	USA	6, 12, 24	Adults up to 60 years of age who meet full DSM–IV research criteria for BED	Cognitive Behavioral Therapy (CBT) = 45<br>Behavioral weight loss (BWL) = 45<br>CBT + BWL (N/A) = 35	BDI-IA; EDE: Global score	Unclear	
Grilo 2014	USA	4, 10, 16	Patients who were obese and met DSM-5 criteria for BED	Placebo = 27<br>Placebo/CBTsh = 25<br>Sibutramine (N/A) = 26<br>Sibutramine/CBTsh (N/A) = 26	BDI-IA; EDE: Global score	Low	
Hunt 2014	UK	3, 15	Male football fans	Control, Wait-list = 373<br>FFIT = 374	PANAS – Positive Affect; PANAS – Negative affect; RSES; SF-12 MCS	High	
Huseinovic 2016	Sweden	3, 12, 24	Women 6–15 week postpartum	Control Group = 56<br>Diet behaviour modification Group = 54	RAND SF-36 MCS	Low	Y
Jackson 2018	Italy	1, 7, 13, 19	Italian women with BED	Brief strategic therapy = 30<br>Cognitive-behavioral therapy = 30	OQ-45.2	Unclear	
Katzer 2008	New Zealand	2.3, 6.3, 14.3	Women with at least one other cardiovascular risk factor.	Mail-delivered 'non-dieting' program (P3) = 101<br>Group 'non-dieting' program (P2) = 62<br>Group 'non-dieting' program plus Relaxation (P1) = 62	90-item GSI scale depression dimension; Anxiety dimension	Unclear	
Mensinger 2016	USA	6, 24	General population	Control, Weight Neutral Program = 40<br>Weight Loss Program = 40	DASS-21; EDE Global Score; RSES;	High	

Messier 2013	USA	6, 18	Ambulatory, community-dwelling persons age 55 years or older with mild or moderate knee osteoarthritis	Exercise only = 150<br>Diet-induced weight loss only = 152<br>Diet-induced weight loss plus exercise = 152	SF-36 MCS	Unclear	
Munsch 2007	Switzerland	4, 16	Adults with BED	Group BWLT = 36<br>Group CBT = 44	BDI; BAI; German versions; Fragebogen zur Lebenszufriedenheit (FLZ); EDE Dietary Restraint; EDE Shape Concern; EDE Weight Concern; EDE Eating Concern;	Unclear	
Ng 2015	UK	4, 12	Chinese patients with moderate to severe obstructive sleep apnoea (OSA) diagnosed on portable home sleep monitoring.	Control group = 43<br>Lifestyle modification program = 61	SF-36 MCS	Unclear	
Ramirez 2001	USA	4, 7, 16	General adult overweight and obese population	Weight control only = 40<br>Weight control plus body image therapy = 48	BDDE-SA; Brief Symptom Inventory; BSQ; EDE Concern; EDE Restraint; RSES	Unclear	
Schubel 2016	Germany	3, 11.5	Adults between 35-65 years, non-smokers and who are overweight or obese.	Control group = 52<br>Continuous Calorie Restriction = 49<br>Intermittent Calorie Restriction = 49	SF-12 MCS	Low	Y
Silva 2010	Portugal	4, 12	Women with overweight or obesity, aged 25 to 50 (and pre-menopausal)	Comparison group = 116<br>Intervention = 123	BSQ; Body Image Assessment questionnaire; Physical Self-Perception Profile: (1) Global physical self-worth subscale; (2) Body attractiveness subscale	Unclear	
Snel 2012	The Netherlands	4, 22	Adults with insulin-dependent Type 2 diabetes mellitus and obesity	VLCD only = 14<br>VLCD + exercise = 13	HADS; HADS – Depression; HADS – Anxiety.	Unclear	
Tapsell 2017	Australia	3, 12	Adult residents, 25-54 years, BMI 25-40kg/m2	Usual care (Control) = 126<br>Intervention Group = 125<br>Intervention plus food supplement group (N/A) = 126	DASS-21; SF-12 MCS	High	Y
The Look AHEAD Research Group 2010	USA	12, 24, 36, 48, 60, 72, 84, 96, 108, 120	Adults with Type 2 Diabetes Mellitus	Diabetes support and education = 2575<br>Intensive lifestyle intervention = 2570	BDI-AI; SF-26 MCS	High	
vanWier 2011	Netherlands	6, 12, 18, 24	General population	Control – Brochure = 460<br>Internet Group = 464<br>Phone Group = 462	EQ-5D 3L Anxiety/depression dimension; RAND SF-36 – emotional wellbeing	Low	Y
vonGruenigen 2008	USA	12	Women with endometrial cancer	Control, Usual care = 22<br>Lifestyle intervention = 23	BDI-II	Unclear	
Wadden 1986	USA	1, 4-6, 16-18, 40-42	General population	VLCD = 18<br>Behaviour = 18<br>Combined = 23	BDI	Unclear	
Zwickert 2016	Australia	3, 6, 9, 15	General population	CBT + Minimal = 29<br>CBT + Intensive = 31	Binge Eating Scale	High	

BAI: Beck Anxiety Inventory**; BDDE-SA:** Body Dysmorphic Disorder Examination - Self-Administration; **BDI:** Beck Depression Inventory; **BDI-IA:** Beck Depression Inventory (1978); **BDI-II:** Beck Depression Inventory-II; **BED:** Binge Eating Disorder**; BIAQ:** Body Image Avoidance Questionnaire; **BMI:** Body Mass Index (kg/m^2); **BSQ:** Body Shape Questionnaire; **CBT:** Cognitive Behaviour Therapy; **DASS-21:** Depression Anxiety Stress Scale; **DPP:** Diabetes Prevention Program; **EDE:** Eating Disorder Examination Interview; **EQ-5D:** EuroQol-Five Dimension; **GHQ-12:** General Health Questionnaire-12; **GSI:** 90-item global severity index scale; **HADS:** Hospital Anxiety and Depression Scale**; IWQOL-Lite:** Impact of Weight on Quality of Life-Lite; **N:** No; **MBSRQ-AS:** Multidimensional Body-Self Relations Questionnaire Appearance Scale; **MBSRQ-BAS:** Multidimensional Body-Self Relations Questionnaire Body Areas Satisfaction Scale; **MH**: Mental Health; **MI**: Motivational interviewing; **N/A:** Not applicable; **NS:** Not specified; **PANAS**: Short Form of the positive and negative affect scale; **PHQ-8:** Patient Health Questionnaire 8; **RSES:** Rosenberg Self-Esteem Scale**; SF-12 MCS:** 12-Item Short Form Survey, Mental Component Summary; **SWLS:** Satisfaction with Life Scale; **Y:** Yes.

*Not all outcome measures collected at all follow-up time points

Table S4. Baseline demographics

Study ID	Groups:	Randomised	Number of participants reported at baseline for primary study	Gender (%F)	Age		BMI		Comorbidities at baseline (%) *		
					Mean	*SD*	*Mean*	*SD*	*CV morbidity*	*Type II DM*	*Hypertension*
Ames 2005	Standard behavioural treatment	NR	13	100	21.5	2.2	NR	NR	NR	NR	NR
	Reformulated cognitive-behavioural treatment	NR	13						NR	NR	NR
Andersen 1999	Diet + Lifestyle Activity	20	20	100	42.9	7.9	32.4	4.5	NR	NR	NR
	Diet + Aerobic Group	20	20	100	43.2	9.1	31.4	3.7	NR	NR	NR
Annesi 2016	Comparison treatment	55	110	100	48.2	7.8	35.3	3.2	NR	NR	NR
	Experimental treatment	55							NR	NR	NR
Annesi 2017	Control comparison group	54	107	100	48.6	7.1	35.4	3.3	NR	NR	NR
	Experimental group	53							NR	NR	NR
Appel 2011	Control (Self-directed)	138	138	63.8	52.9	10.1	36.8	5.1	NR	23.8	76.8
	Remote Support Only (N/A)	139	N/A	N/A	N/A	N/A	N/A	N/A	N/A	N/A	N/A
	In-Person Support	138	138	63.8	53.3	10.5	36.8	5.2	NR	23.9	71.0
Ard 2004	"advice only" comparison group	273	273	63	49.5	8.8	32.9	5.6	NR	NR	14.0
	"established" behavioural intervention group	268	268	64.9	50.2	8.6	33.0	5.5	NR	NR	13.7
	Established + DASH Intervention Group	269	269	57.2	50.2	9.2	33.3	6.3	NR	NR	13.8
Ard 2018	Exercise Only	54	54	68.5	69.9	4.5	33.9	0.4	NR	NR	NR
	Exercise + Diet Quality + Weight Maintenance	55	55	60	70.5	4.8	33.8	0.4	NR	NR	NR
	Exercise + Diet Quality + Weight Loss	55	55	58.2	70.3	4.8	33.3	0.4	NR	NR	NR
Ash 2006	Control Group - Booklet only	63	54	77.8	47	14	35.8	6.2	NR	NR	NR
	Individualised Dietetic Treatment	66	65	75.4	48	13	34.2	5.9	NR	NR	NR
	Fat Booters Incorporated	62	57	66.7	49	13	33.7	4.6	NR	NR	NR
Bacon 2002	Health at Every Size - control	NR	29	100	39.3	4.5	35.9	4.1	NR	NR	NR
	Diet Group - intervention	NR	23				36.6	4.1	NR	NR	NR
Barnes 2017	Treatment as usual (N/A)	30	30	N/A	N/A	N/A	N/A	N/A	N/A	N/A	N/A
	Nutrition - ATTENTION CONTROL	29	29	69	48.9	11.6	35.1	7.5	NR	NR	NR
	Motivational interviewing	30	30	80	47.1	10.0	34.7	7.1	NR	NR	NR
Bennett 2012	Control, Usual care	185	185	65.9	54.7	11.0	36.99	5.2	NR	NR	NR
	Be Fit, Be Well	180	180	71.1	54.6	10.8	37.03	5.0	NR	NR	NR
Bennett 2013	Control, usual care	97	94	100	35.2	5.5	30.2	2.4	NR	5.3	36.2
	Weight gain prevention intervention	97	91	100	35.6	5.5	30.1	2.7	NR	5.8	36.3
Beutel 2006	Behavioural therapy	130	130	85	42.3	20-60[c]	43.9	35.2, 73.3[c]	NR	NR	NR
	Psychodynamic treatment	137	137	86	40.3	20-64[c]	44.6	35.1, 73.5[c]	NR	NR	NR
Burke 2015	Standard behavioural weight loss treatment	72	72	83.1	53	9.6	33.2	4.11	NR	NR	NR
	Self-efficacy enhancement plus standard behavioural weight loss treatment	58	58								
Cleo 2018	Wait list control (N/A)	25	N/A	N/A	N/A	N/A	N/A	N/A	N/A	N/A	N/A
	TTT Top Ten Tips habit formation	25	25	80	48.2	11.3	34.6	5.2	NR	NR	NR

Study ID	Groups:	Randomised	Number of participants reported at baseline for primary study	Gender (%F)	Age		BMI		Comorbidities at baseline (%) *		
					Mean	*SD*	*Mean*	*SD*	*CV morbidity*	*Type II DM*	*Hypertension*
	DSD Do Something Different online software	25	25	76	51.3	10.0	35.2	7.4	NR	NR	NR
Conroy 2015	Self-guided	50	49	100	54	5.6	33.4	5.4	NR	23.5	56.1
	Interventionist led	49	49	100	53.8	5.3	36.1	5.4	NR		
Damschroder 2014	Control, MOVE - usual care	159	159	12.6	54.6	10.5	36.8	6.4	NR	37.7	65.4
	ASPIRE group, individual telephone counselling	162	162	16	55.4	10.0	36.3	6.2	NR	32.7	67.9
	ASPIRE group, group counselling	160	160	16.2	54.9	9.5	36.2	6.1	NR	40.0	65.6
Daubenmier 2016	Active control intervention	94	94	86	47.8	12.4	35.6	3.8	NR	NR	22.3
	Mindfulness Intervention	100	100	79	47.2	13.0	35.4	3.5	NR	NR	16
de Zwaan 2017	Internet-based guided self-help treatment	89	83	89.2	43.7	12.7	33.4	3.9	NR	8.4[#]	19.3
	Cognitive Behavioural Therapy	89	86	86	42.7	12.0	34.4	3.9	NR	3.5[#]	26.7
deRoon 2017	Control	48	48	100	60	4.9	29.5	2.6	NR	NR	NR
	Diet	97	97	100	61	4.6	29.5	2.6	NR	NR	NR
	Exercise	98	98	100	59	4.9	29	2.9	NR	NR	NR
Diabetes Prevention Program R G 2009	Placebo	1082	1082	69	50.3	10.4	32.2	6.7	NR	All at high risk of T2DM	30.0
	Metformin (N/A)	1073	1073	N/A	N/A	N/A	N/A	N/A	N/A		
	Lifestyle	1079	1079	68	50.6	11.3	33.9	6.8	NR		
Fernandez-Ruiz 2018	Control	37	37	51.4	62.8	8.9	34.3	4.5	NR	62.2	86.5
	Intervention (healthy eating, exercise & CBT)	37	37	48.6	59.4	9.1	32.4	3.8	NR	43.2	78.4
Foley 2016	Usual care (Control)	175	175	68	50.5	8.7	35.9	3.7	NR	3.4	29.1
	Weight loss intervention	176	176	68	50.9	9.1	35.9	4.1	NR	3.4	29.5
Forman 2016	Standard Behavioural Treatment	90	90	82.1	51.6	10.2	37.4	6.2	NR	NR	NR
	Acceptance-Based Treatment	100	100		51.6	10.0	36.5	5.4	NR	NR	NR
Foster-Schubert 2012	Control- usual care	87	87	100	57.4	4.4	30.7	3.9	NR	NR	NR
	Calorie reduced diet	118	118	100	58.1	5.9	31	3.9	NR	NR	NR
	Aerobic exercise (N/A)	117	117	N/A	N/A	N/A	N/A	N/A	N/A	N/A	N/A
	Intervention - diet and exercise	117	117	100	58.0	4.5	31	4.3	NR	NR	NR
Freitas 2017	Weight loss program + Sham	27	25	100	48.5	9.6	37.2	2.1	NR	NR	48.0
	Weight loss program + Exercise	28	26	96	45.9	7.7	38.1	2.8	NR	NR	38.5
Goodwin 2014	Mailed-based intervention	167	167	100	60.4	7.8	31.1	5.3	NR	0.0	NR
	Individual lifestyle intervention	171	171	100	61.6	6.7	31.4	5.0	NR	0.0	NR
Grilo 2011	Cognitive Behavioral Therapy (CBT)	45	45	64.4	45.2	8.5	39.3	6.1	NR	NR	NR
	Behavioral weight loss (BWL)	45	45	62.2	44.6	10.5	38	5.3	NR	NR	NR
	CBT + BWL (N/A)	35	NR						N/A	N/A	N/A
Grilo 2014	Placebo	27	27	66.7	43.2	12.4	39.3	5.5	NR	NR	NR
	Placebo/CBTsh	25	25	80	45.7	12.4	36.5	5.3	NR	NR	NR
	Sibutramine (N/A)	26	NR	N/A	N/A	N/A	N/A	N/A	N/A	N/A	N/A
	Sibutramine/CBTsh (N/A)	26	NR	N/A	N/A	N/A	N/A	N/A	N/A	N/A	N/A
Hunt 2014	Control, Wait-list	373	373	0	47.2	7.89	35.1	4.8	NR	NR	NR
	FFIT	374	374	0	47	8.07	35.5	5.1	NR	NR	NR

Study ID	Groups:	Randomised	Number of participants reported at baseline for primary study	Gender (%F)	Age		BMI		Comorbidities at baseline (%) *		
					Mean	*SD*	*Mean*	*SD*	*CV morbidity*	*Type II DM*	*Hypertension*
Huseinovic 2016	Control Group	56	56	100	32.6	4.7	31[a]	26.3–48.7[c]	NR	NR	NR
	Diet behaviour modification Group	54	54	100	31.8	4.5			NR	NR	NR
Jackson 2018	Brief strategic therapy	30	30	100	45.9	10.8	39.6	2.6	NR	NR	NR
	Cognitive-behavioral therapy	30	30	100	46.2	10.5			NR	NR	NR
Katzer 2008	Mail-delivered 'non-dieting' program (P3)	101	225	100	46.1	8.9	35.4	5.7	NR	NR	NR
	Group 'non-dieting' program (P2)	62							NR	NR	NR
	Group 'non-dieting' program plus Relaxation (P1)	62							NR	NR	NR
Mensinger 2016	Control, Weight Neutral Program	40	40	100	39.8	4.34	37.4	0.6	NR	NR	NR
	Weight Loss Program	40	40	100	39.4	3.91	38.6	0.7	NR	NR	NR
Messier 2013	Exercise only	150	150	72	66	6	33.5	3.7	8.0	12.0[#]	59.3
	Diet-induced weight loss only	152	152	71	66	6	33.7	3.8	12.5	11.8[#]	61.2
	Diet-induced weight loss plus exercise	152	152	72	65	6	33.6	3.7	7.2	15.1[#]	59.9
Munsch 2007	Group BWLT	36	36	86.1	47.8	11.8	34.4	3.7	NR	NR	NR
	Group CBT	44	44	90.9	44.4	11.5	33.7	4.3	NR	NR	NR
Ng 2015	Control group	43	43	30.7	52	9.3	30.5	4.2	NR	25.6[#]	20.9
	Lifestyle modification program	61	61	21.3	51.4	9.1	30.2	3.9	NR	23.0[#]	26.2
Ramirez 2001	Weight control only	40	65	NR	44	9.7	33.8	5.1	NR	NR	NR
	Weight control plus body image therapy	48		NR					NR	NR	NR
Schubel 2016	Control group	52	52	52	50.7	7.1	31.1	3.6	NR	0	NR
	Continuous Calorie Restriction	49	49	49	50.5	8.0	31.2	4.0	NR	0	NR
	Intermittent Calorie Restriction	49	49	49	49.4	9.0	32	3.8	NR	0	NR
Silva 2010	Comparison group	116	116	100	37.1	6.99	31.3	4.00	NR	NR	NR
	Intervention	123	123	100	38.1	7.04	31.7	4.24	NR	NR	NR
Snel 2012	VLCD only	14	14	38.5	56	2	37.9	1.4	NR	100	NR
	VLCD + exercise	13	13	57.1	53	3	36.4	1.1	NR	100	NR
Tapsell 2017	Usual care (Control)	126	126	73	43.8	7.46	32.49	4.12	NR	NR	11.1
	Intervention Group	125	124	73	43.79	7.97	32.59	4.25	NR	NR	16.1
	Intervention plus food supplement group (N/A)	126	N/A	N/A	N/A	N/A	N/A	N/A	N/A	N/A	N/A
The Look AHEAD Research Group 2010	Diabetes support and education	2575	2575	59.6	58.9	6.9	36	5.8	NR	100.0	84.0
	Intensive lifestyle intervention	2570	2570	59.3	58.6	6.8	35.9	6.0	NR	100.0	84.5
vanWier 2011	Control – Brochure	460	460	33.5	43	8.7	29.6	3.7	2.0	2.0[#]	10.0
	Internet Group	464	464	34.9	43	8.4	29.6	3.4			
	Phone Group	462	462	30.5	43	8.8	29.5	3.5			
von Gruenigen 2008	Control, Usual care	22	22	100	55.4	7.5	41.1	10.3	NR	NR	NR
	Lifestyle intervention	23	23	100	54.0	9.6	43.5	10.1	NR	NR	NR
Wadden 1986	VLCD	18	15	84.7	44.3	8.7	NR	NR	NR	NR	NR
	Behaviour	18	16		44.3	8.6	NR	NR	NR	NR	NR

Study ID	Groups:	Randomised	Number of participants reported at baseline for primary study	Gender (%F)	Age		BMI		Comorbidities at baseline (%) *		
					Mean	*SD*	*Mean*	*SD*	*CV morbidity*	*Type II DM*	*Hypertension*
	Combined	23	19		43.6	7.8	NR	NR	NR	NR	NR
Zwickert 2016	CBT + Minimal	29	29	71.6	44.3	19-64[c]	37.5	30.4—54.8[c]	NR	NR	NR
	CBT + Intensive	31	31						NR	NR	NR

CBT: Cognitive Behaviour Therapy; **CV** = Cardiovascular; **DM:** Diabetes Mellitus; **DPP:** Diabetes Prevention Program**; N/A** = Not applicable; **NR** = Not reported; **VLCD:** Very low calorie diet
* Comorbidity definitions varied for each study; [#]Unclear whether DM percentage listed includes Type II and Type I
[a] Median (IQR); [b] Standard error; [c] Range; [d] 95% Confidence intervals

Table S5. Intervention characteristics

Study ID	Groups:	Intervention type	Intervention faded in intensity	Features (meal replacements, nutrition education, financial incentives, intermittent fasting, content designed to help participants following programme end)	Provider [a]	Provider training received	Delivery		Intervention setting	Intervention timing (months)		Sessions			Intervention personalised, titrated or adapted
							Mode	Format		Last contact	End (step change in intensity)	N[c]	Frequency	Length per session with description for varying lengths. (minutes [b])	
Ames 2005	Standard behavioural treatment	Diet and exercise	No	Nutrition Edu.	Psychologist/ Counsellor	No	Group	Face to Face		12	6	20	Approx. weekly (20 sessions in 6m)		No
	Reformulated cognitive-behavioural treatment	Diet and exercise	No	Nutrition Edu.; Help following programme end	Psychologist/ Counsellor	No	Group	Face to Face		12	6	20	Approx. weekly (20 sessions in 6m)		No
Andersen 1999	Diet + Lifestyle Activity	Diet and exercise	No	Nutrition Edu.; Help following programme end	Psychologist/ Counsellor	No	Group	Face to Face; Other	Community	17	4	16	Weekly	Cognitive behavioural sessions = 60 mins. Advised 30 mins moderate physical activity on most days per week.	No
	Diet + Aerobic Group	Diet and exercise	No	Nutrition Edu.; Help following programme end	Other	No	Group	Face to Face	Community	17	4	64	Weekly cognitive behavioural session + aerobic session 3 x per week	Cognitive behavioural sessions = 60 mins. Aerobics classes: 5-10 min warm up, 15-45 mins aerobic phase (increased by 4 mins per week), 5 min cool-down.	Yes
Annesi 2016	Comparison treatment	Diet and exercise	No	Nutrition Edu.		Yes	Individual	Telephone; Print	Community	24	6	12	Every 2 weeks	15	Yes
	Experimental treatment	Diet and exercise	No	Nutrition Edu.; Help following programme end	Psychologist/ Counsellor	Yes	Individual and Group	Face to Face; Internet	Community	24	6.5	32	Exercise support sessions: over 6.5 months; Nutrition	Six 45-minute individual exercise meetings; 10	Yes

Study ID	Groups:	Intervention type	Intervention faded in intensity	Features (meal replacements, nutrition education, financial incentives, intermittent fasting, content designed to help participants following programme end)	Provider [a]	Provider training received	Delivery		Intervention setting	Intervention timing (months)		Sessions			Intervention personalised, titrated or adapted
							Mode	Format		Last contact	End (step change in intensity)	N[c]	Frequency	Length per session with description for varying lengths. (minutes [b])	
													sessions: every 2 weeks	nutrition sessions of 60 minutes	
Annesi 2017	Control comparison group	Diet and exercise	No	Nutrition Edu.; Inter. Fasting		Yes	Individual	Telephone; Print	Community	6	6	17	Every second week (24 weeks in total)	Lesson followed by 15 min phone conversation	Yes
	Experimental group	Diet and exercise	Yes	Nutrition Edu.; Help following programme end	Health Trainer	Yes	Individual and Group	Face to Face	Community	12	6	35	Monthly 1:1 for 6m. Biweekly from weeks 10-52	45 mins for 1:1 sessions; Group length not stated	Yes
Appel 2011	Control (Self-directed)	Control	No	Nutrition Edu.	Health Trainer	Unclear	Individual	Face to Face; Print	Health Care			1	0, 24m		
	Remote Support Only (N/A)	N/A	N/A	N/A	N/A	N/A	N/A	N/A	N/A	N/A	N/A	N/A	N/A	N/A	N/A
	In-Person Support	Diet and exercise	Yes	Nutrition Edu.	Other AHPs; Health Trainer	Yes	Individual and Group	Face to Face; Telephone; Internet; Other	Health Care	24	6	57	Weekly (0-3m); Three monthly contacts over the next 3 months; Two monthly contacts for the remainder of the study.	Individual sessions approx. 20 mins; In-person group sessions: 90 mins	Yes
Ard 2004	"advice only" comparison group	Diet and exercise	No	Nutrition Edu.	Dietitian	No	Individual	Face to Face; Print;	Health Care	6	6	2		30	Yes
	"established" behavioural intervention group	Diet and exercise	Yes	Nutrition Edu.; Help following programme end			Individual and Group	Face to Face	Health Care	18	6	23	Weekly (3m), Biweekly (3m), Monthly (12m)		Yes
	Established + DASH Intervention Group	Diet and exercise	Yes	Nutrition Edu.; Help following programme end			Individual and Group	Face to Face	Health Care	18	6	23	Weekly (3m), Biweekly (3m), Monthly (12m)		Yes
Ard 2018	Exercise Only	Exercise only	No			Unclear	Group	Face to Face; Print	Community	12	6	38	Weekly 0-24 weeks and then biweekly until 12m	1 hour	No

Study ID	Groups:	Intervention type	Intervention faded in intensity	Features (meal replacements, nutrition education, financial incentives, intermittent fasting, content designed to help participants following programme end)	Provider [a]	Provider training received	Delivery		Intervention setting	Intervention timing (months)		Sessions			Intervention personalised, titrated or adapted
							Mode	Format		Last contact	End (step change in intensity)	N[c]	Frequency	Length per session with description for varying lengths. (minutes [b])	
	Exercise + Diet Quality + Weight Maintenance	Diet and exercise	No	Nutrition Edu.		Unclear	Group	Face to Face; Print;	Community	12	6	38	Weekly 0-24 weeks and then biweekly until 12m	1 hour	No
	Exercise + Diet Quality + Weight Loss	Diet and exercise	No	Nutrition Edu.		Unclear	Group	Face to Face; Print	Community	12	6	38	Weekly 0-24 weeks and then biweekly until 12 m	1 hour	No
Ash 2006	Control Group - Booklet only	Control	No	Nutrition Edu.		Yes	Other – booklet only	Print	Community			0			No
	Individualised Dietetic Treatment	Diet and exercise	Yes	Nutrition Edu.	Dietitian	Yes	Individual	Face to Face; Telephone; Print	Health Care; Community	6	2	12	Weekly for 8 weeks, monthly from week 8 until 6m.	1 hour initial individual consultation, followed by seven 20-min weekly review sessions and four monthly follow-up sessions	Yes
	Fat Booters Incorporated	Diet and exercise	Yes	Nutrition Edu.; Help following programme end	Dietitian; Nutritionist	Yes	Group	Face to Face; Telephone; Print	Health Care; Community	6	2	11	Weekly for 6 weeks with monthly follow up until 6m.	1.5 hrs for 6 sessions. Not stated for 5 follow up visits.	No
Bacon 2002	Health at Every Size - control	Diet and exercise	No	Nutrition Edu.	Psychologist/ Counsellor		Group	Face to Face	Community	12	6	30	Weekly for first 6m, then monthly	1.5 hour	No
	Diet Group - intervention	Diet and exercise	No	Nutrition Edu.	Dietitian		Group	Face to Face	Community	12	6	30	Weekly for first 6m, then monthly	1.5 hour	No
Barnes 2017	Treatment as usual (N/A)	Control	N/A	N/A	N/A	N/A	N/A	N/A	N/A	N/A	N/A	N/A	N/A	N/A	N/A
	Nutrition - ATTENTION CONTROL	Diet only	No	Nutrition Edu.	Other AHPs		Individual	Face to Face Internet; Print	Community	3	3	5	Every 3 weeks	60 mins the first session and 20 mins the rest (4 sessions)	
	Motivational interviewing	Diet and exercise	No	Nutrition Edu.			Individual	Face to Face;	Community	3	3	5	Every 3 weeks	60 mins the first session and 20	

Study ID	Groups:	Intervention type	Intervention faded in intensity	Features (meal replacements, nutrition education, financial incentives, intermittent fasting, content designed to help participants following programme end)	Provider [a]	Provider training received	Delivery		Intervention setting	Intervention timing (months)		Sessions			Intervention personalised, titrated or adapted
							Mode	Format		Last contact	End (step change in intensity)	N[c]	Frequency	Length per session with description for varying lengths. (minutes [b])	
								Internet; Print						mins the rest (4 sessions)	
Bennett 2012	Control, Usual care	Diet only	No			No	Other – Print only	Print							No
	Be Fit, Be Well	Diet and exercise	Yes	Nutrition Edu.	Health care professional (not specified)	Yes	Individual and Group	Face to Face; Telephone; Internet	Community; Home	24	12	30	Monthly for the first year and bimonthly for the second year. Additional 12 optional monthly group sessions	15-20 mins; Telephone counselling sessions were held monthly for the first year and bimonthly for the second year. There were an additional 12 optional monthly group sessions	Yes
Bennett 2013	Control, usual care	Control	No			No				12					No
	Weight gain prevention intervention	Diet and exercise	No	Nutrition Edu.		Yes	Individual	Telephone;	Community; Home	12	12	64	Weekly (52) and monthly (12)	10 mins (52 weekly IVR (interactive voice response calls)) 12 monthly 20 min calls	Yes
Beutel 2006	Behavioural therapy	Diet and exercise	No	Help following programme end	Physician; Psychologist/ Counsellor	Yes	Group	Face to Face	Inpatient	1.5	1.5			40	Yes
	Psychodynamic treatment	Diet and exercise	No		Physician; Psychologist/ Counsellor	Yes	Individual and Group	Face to Face	Inpatient	1.5	1.5			40	Yes
Bliddal 2011	Control, low-energy diet	Diet only	Yes	Nutrition Edu.	Dietitian	Unclear	Group	Face to Face	Community			5	Weeks: 0, 8, 32, 36, 52	2 hours	No
	Intensive low-energy diet	Diet only	Yes	MR-F; Nutrition Edu.	Dietitian	Unclear	Group	Face to Face	Community; Home	12	8.3	44	Baseline, weekly for 32 weeks then every 2 weeks	1.5 hours	No

Study ID	Groups:	Intervention type	Intervention faded in intensity	Features (meal replacements, nutrition education, financial incentives, intermittent fasting, content designed to help participants following programme end)	Provider [a]	Provider training received	Delivery		Intervention setting	Intervention timing (months)		Sessions			Intervention personalised, titrated or adapted
							Mode	Format		Last contact	End (step change in intensity)	N[c]	Frequency	Length per session with description for varying lengths. (minutes [b])	
Burke 2015	Standard behavioural weight loss treatment	Diet and exercise	Yes	Nutrition Edu.	Health care professional (not specified)	No	Group	Face to Face	Community; Home	18	12	20	Weekly the first month, biweekly the second month, monthly for next 10 months, and every 6 weeks for 13-18m.	1 hour group sessions	
	Self-efficacy enhancement plus standard behavioural weight loss treatment	Diet and exercise	Yes	Nutrition Edu.	Health care professional (not specified)	No	Individual and Group	Face to Face; Telephone	Community; Home	18	12	50	SE - 1:1 every 2 weeks for first 12m. Then at least monthly. SBT: weekly the first month, biweekly the second month, monthly for next 10 months, and every 6 weeks for months 13-18.	1 hour group session. 1:1 sessions: 23 mins.	
Cleo 2018	TTT Top Ten Tips habit formation	Diet and exercise	No			No	Group	Face to Face; Print	Community; Home	3	3	13	Weekly phone calls	2 hrs group induction; Call length not stated.	No
	DSD Do Something Different online software	Diet and exercise	No			No	Individual and Group	Face to Face; Telephone; Internet	Community; Home	3	3	13	Weekly tasks and phone calls	2 hrs group induction. Call length not stated. tasks, length not stated	Yes
	Wait list control (N/A)	N/A	N/A	N/A	N/A	N/A	N/A	N/A	N/A	N/A	N/A	N/A	N/A	N/A	N/A
Conroy 2015	Self-guided	Diet and exercise	No	Nutrition Edu.	Other	No	Other - Self-guided manual	Print	Home	3	3			12-week self-guided manual	No
	Interventionist led	Diet and exercise	No	Nutrition Edu.	Physician; Other	No	Group	Face to Face	Health Care	3	3	12	Weekly	60 mins	No
Damschroder 2014	Control, MOVE - usual care	Diet and exercise	Yes	Nutrition Edu.	Nurse (General); Psychologist/ Counsellor;	Unclear	Group	Face to Face	Health Care	24	3	58	Weekly for 3m, then either quarterly or twice monthly	11-12 weekly open-group sessions of 90 mins each over 3	Yes

Study ID	Groups:	Intervention type	Intervention faded in intensity	Features (meal replacements, nutrition education, financial incentives, intermittent fasting, content designed to help participants following programme end)	Provider [a]	Provider training received	Delivery		Intervention setting	Intervention timing (months)		Sessions			Intervention personalised, titrated or adapted
							Mode	Format		Last contact	End (step change in intensity)	N[c]	Frequency	Length per session with description for varying lengths. (minutes [b])	
					Dietitian; Physiotherapist									months. During months 4-12, one group met quarterly for 90 minutes and the other groups met twice a month for 60 minutes. Some participants had the option of re-enrolling in the initial series of weekly sessions. Total hours over the year ranged from 22 to 35 hours. 12-24 mths as above for mths 4-12.	
	ASPIRE group, individual telephone counselling	Diet and exercise	Yes	Nutrition Edu.	Health Trainer	Yes	Individual	Telephone	Home	24	3	34	Same as ASPIRE-Group but duration of sessions varied	Up to 30 mins for the first 3 mths and 20 mins for the remaining 9 mths, totalling 11 hours across the year. 12-24 mths coaching every other mth, 6 sessions.	Yes
	ASPIRE group, group counselling	Diet and exercise	Yes	Nutrition Edu. Help following programme end	Health Trainer	Yes	Group	Face to Face	Health Care	24	3	34	Both ASPIRE small-changes treatment arms consisted of weekly sessions for 3m, followed by 6m of sessions every other week, and	Up to 90 mins for the first 3 mths and 60 mins for the remaining 9 mths, totalling 33 hours across the year. 12-24 mths	Yes

Study ID	Groups:	Intervention type	Intervention faded in intensity	Features (meal replacements, nutrition education, financial incentives, intermittent fasting, content designed to help participants following programme end)	Provider [a]	Provider training received	Delivery		Intervention setting	Intervention timing (months)		Sessions			Intervention personalised, titrated or adapted
							Mode	Format		Last contact	End (step change in intensity)	N[c]	Frequency	Length per session with description for varying lengths. (minutes [b])	
													then 3 monthly sessions over 12 months, for a total of 28 sessions.	coaching every other mth, 6 sessions.	
Daubenmier 2016	Active control intervention	Diet and exercise	Yes	Nutrition Edu.	Dietitian	Unclear	Group	Face to Face; Print	Community	5.5	5.5	16	12 weekly then biweekly for 3 sessions, and then one session one month later plus a single all-day weekend session	16 sessions: 2 hours; 5-hour all-day session	Yes
	Mindfulness Intervention	Diet and exercise	Yes	Nutrition Edu.; Help following programme end	Dietitian	Yes	Group	Face to Face; Print	Community	5.5	5.5	16	12 weekly then biweekly for 3 sessions, and then one session one month later plus a single all-day weekend session	16 sessions: 2.5 hours; 6.5 hour all day session	Yes
de Zwaan 2017	Internet-based guided self-help treatment		No	Help following programme end		No	Individual	Internet	Home	4	4	17-18	Participants email coach once per week. Coach emails back once per week.	No indication of time taken to complete each of the 11 modules is given.	No
	Cognitive Behavioural Therapy	Diet and exercise	No	Help following programme end		No	Individual	Face to Face	Health Care	4	4	20	Twice weekly for first month, then once weekly during remaining 3 months.	50 mins; Participants also given additional homework: expected time to complete this is not given.	Yes
deRoon 2017	Control	Control	No			No		Telephone	Home	4					No
	Diet	Diet only	No	Nutrition Edu.	Dietitian	No	Individual and Group	Face to Face; Telephone;	Community; Home	12	4		Weekly		No

Study ID	Groups:	Intervention type	Intervention faded in intensity	Features (meal replacements, nutrition education, financial incentives, intermittent fasting, content designed to help participants following programme end)	Provider [a]	Provider training received	Delivery		Intervention setting	Intervention timing (months)		Sessions			Intervention personalised, titrated or adapted
							Mode	Format		Last contact	End (step change in intensity)	N[c]	Frequency	Length per session with description for varying lengths. (minutes [b])	
	Exercise	Diet and exercise	No		Dietitian; Physiotherapist	No	Group	Face to Face; Telephone	Community; Home	12	4	32	2 x week groups sessions;<br>2 x Nordic walking	4 hours per week exercise program; two x 1 hour group sessions;<br>2 x 1 hour sessions Nordic walking per week - individual home-based exercise with 'Supervised lessons of Nordic walking by instructors are organised to increase motivation and compliance." (4 hours per week for 16 weeks);<br>Unclear if all Nordic walking sessions are organised - did not contribute to total N of sessions	No
Diabetes Prevention Program R G 2009	Placebo	Diet and exercise	No	Nutrition Edu.	Other		Individual	Face to Face; Print		36	36	4	Annually	20 – 30	
	Metformin (N/A)	N/A	N/A	N/A	N/A	N/A	N/A	N/A	N/A	N/A	N/A	N/A	N/A	N/A	N/A
	Lifestyle	Diet and exercise	Yes	MR-P; Nutrition Edu.; Help following programme end	Psychologist/ Counsellor; Dietitian; Exercise physiologist; Health Trainer	Yes	Individual and Group	Face to Face; Telephone; Print	Community	36	6	358	16 sessions in first 24 weeks then monthly. [At least 2 exercise classes per week]	45 mins;<br>Core curriculum sessions 30-60 mins;<br>Sessions: 16+6+12+12=46. [Physical activity	Yes

Study ID	Groups:	Intervention type	Intervention faded in intensity	Features (meal replacements, nutrition education, financial incentives, intermittent fasting, content designed to help participants following programme end)	Provider [a]	Provider training received	Delivery		Intervention setting	Intervention timing (months)		Sessions			Intervention personalised, titrated or adapted
							Mode	Format		Last contact	End (step change in intensity)	N[c]	Frequency	Length per session with description for varying lengths. (minutes [b])	
														2x52 x 3 years = 312]	
Fernandez-Ruiz 2018	Control	Control	No			Unclear									No
	Intervention (healthy eating, exercise & CBT)	Diet and exercise	No	Nutrition Edu.	Nurse (General); Physician; Psychologist/ Counsellor; Nutritionist; Exercise physiologist	Unclear	Individual and Group	Face to Face	Health Care	12	12	232	4 x per week physical activity; monthly CBT & health ed.	208 exercise sessions: 4 x per week, 40 mins. CBT 12 sessions, 1 per month, 60 mins. Health education (nurse) 12 sessions, 1 per month, 60 mins.	Yes
Foley 2016	Usual care (Control)	Control	No			No	Other	Print	Health Care						No
	Weight loss intervention	Diet and exercise	Yes	Nutrition Edu.	Psychologist/ Counsellor; Health care professional (not specified); Other	No	Individual	Face to Face; Telephone; Internet; App; Print; SMS	Health Care; Home	12	3	18	Calls 1-4: weekly; Calls 5-10: biweekly Calls 11-18: monthly		No
Forman 2016	Standard Behavioural Treatment	Diet and exercise	Yes	Nutrition Edu.	Physician		Individual and Group	Face to Face	Community	12	6	25	Weekly for 16 sessions, biweekly for 5 sessions, monthly for 2 sessions, and bimonthly for 2 sessions	75	
	Acceptance-Based Treatment	Diet and exercise	Yes	Nutrition Edu.; Help following programme end	Physician		Individual and Group	Face to Face	Community	12	6	25	Weekly for 16 sessions, biweekly for 5 sessions, monthly for 2 sessions, and bimonthly for 2 sessions	75	

Study ID	Groups:	Intervention type	Intervention faded in intensity	Features (meal replacements, nutrition education, financial incentives, intermittent fasting, content designed to help participants following programme end)	Provider [a]	Provider training received	Delivery		Intervention setting	Intervention timing (months)		Sessions			Intervention personalised, titrated or adapted
							Mode	Format		Last contact	End (step change in intensity)	N[c]	Frequency	Length per session with description for varying lengths. (minutes [b])	
Foster-Schubert 2012	Control- usual care	Control	No			No	Other – no contact								
	Calorie reduced diet	Diet only	Yes	Nutrition Edu.	Dietitian	No	Individual and Group	Face to Face; Telephone; Internet	Community; Home	12	6	38	2 + 24 weekly 0-24. Then 2 per month (12) during weeks 24 – 52		Yes
	Aerobic exercise (N/A)	N/A	N/A	N/A	N/A	N/A	N/A	N/A	N/A	N/A	N/A	N/A	N/A	N/A	N/A
	Intervention - diet and exercise	Diet and exercise	Yes	Nutrition Edu.	Dietitian; Exercise physiologist;	No	Individual and Group	Face to Face; Telephone; Internet	Community; Home	12	6	194	3 per week exercise. Plus 38 diet sessions.	45	Yes
Freitas 2017	Weight loss program + Sham	Diet only	No	Nutrition Edu.	Psychologist/ Counsellor; Nutritionist; Physiotherapist	Unclear	Individual and Group	Face to Face	Community	3	3	36	Weekly therapy sessions; two weekly exercises sessions	60	Yes
	Weight loss program + Exercise	Diet and exercise	No	Nutrition Edu.	Psychologist/ Counsellor; Nutritionist; Physiotherapist	Unclear	Individual and Group	Face to Face	Community	3	3	36	Weekly therapy sessions; two weekly exercises sessions	60	Yes
Goodwin 2014	Mailed-based intervention	Diet and exercise	No	Nutrition Edu.		No	Individual	Print	Community	12	12	2	0, 12m		No
	Individual lifestyle intervention	Diet and exercise	Yes	Nutrition Edu.; Help following programme end	Health Trainer	Unclear	Individual	Telephone; Print	Community	12	6	19	Weekly (0-1m); Biweekly (2-3m); Monthly (4-6m); every 2 months (7-12m); every 3 months (13-24m)	30 – 60	Yes
Grilo 2011	Cognitive Behavioral Therapy (CBT)	Diet only	No	Nutrition Edu.	Psychologist/ Counsellor	Yes	Group	Face to Face	Health Care	5.5	5.5	16	16 sessions over 24 weeks	60	No

Study ID	Groups:	Intervention type	Intervention faded in intensity	Features (meal replacements, nutrition education, financial incentives, intermittent fasting, content designed to help participants following programme end)	Provider [a]	Provider training received	Delivery		Intervention setting	Intervention timing (months)		Sessions			Intervention personalised, titrated or adapted
							Mode	Format		Last contact	End (step change in intensity)	N[c]	Frequency	Length per session with description for varying lengths. (minutes [b])	
	Behavioral weight loss (BWL)	Diet and exercise	No	Nutrition Edu.	Psychologist/ Counsellor	Yes	Group	Face to Face	Health Care	5.5	5.5	16	16 sessions over 24 weeks	60	No
	CBT + BWL (N/A)	N/A	N/A	N/A	N/A	N/A	N/A	N/A	N/A	N/A	N/A	N/A	N/A	N/A	N/A
Grilo 2014	Placebo	Control	No		Physician	Unclear				4	4	0			No
	Placebo/CBTsh	Diet only	No	Nutrition Edu.; Help following programme end	Physician	Yes	Individual	Face to Face; Print	Health Care	4	4	1			No
	Sibutramine (N/A)	N/A	N/A	N/A	N/A	N/A	N/A	N/A	N/A	N/A	N/A	N/A	N/A	N/A	N/A
	Sibutramine/ CBTsh (N/A)	N/A	N/A	N/A	N/A	N/A	N/A	N/A	N/A	N/A	N/A	N/A	N/A	N/A	N/A
Hunt 2014	Control, Wait-list	Control	No	Nutrition Edu.				Print							
	FFIT	Diet and exercise	Yes	Nutrition Edu.; Help following programme end	Personal Trainer	Yes	Group	Face to Face; Print	Community	12	3	19	Weekly for 12 weeks. 6 emails over 9 months. 1 reunion at 6 months.	1.5 hours for 12 weekly sessions. Then 6 emails over 9m and 1 x reunion at 6m.	Yes
Huseinovic 2016	Control Group	Diet only	No	Nutrition Edu.		No	Other - leaflet at baseline	Print;	Health Care	0	0				No
	Diet behaviour modification Group	Diet and exercise	No	Nutrition Edu.; Help following programme end	Dietitian	Unclear	Individual	Face to Face; Telephone; Internet; Print; SMS	Health Care	12	3	4	Single face-to-face; biweekly SMS (3) followed by biweekly calls (3); monthly emails (9)	1.5 hours single face-to-face session; Call length not stated.	Yes
Jackson 2018	Brief strategic therapy	Diet and exercise	Yes	Nutrition Edu.	Psychologist/ Counsellor; Dietitian; Exercise physiologist	Unclear	Individual and Group	Face to Face; Telephone	Inpatient; Home	7	7	44	1m inpatient phase (2 weekly nutrition education sessions, 5 weekly physical activity classes, 2 weekly psychotherapy sessions);	45 min nutrition education; 45 min psychotherapy sessions; Telephone call length not stated.	Yes

Study ID	Groups:	Intervention type	Intervention faded in intensity	Features (meal replacements, nutrition education, financial incentives, intermittent fasting, content designed to help participants following programme end)	Provider [a]	Provider training received	Delivery		Intervention setting	Intervention timing (months)		Sessions			Intervention personalised, titrated or adapted
							Mode	Format		Last contact	End (step change in intensity)	N[c]	Frequency	Length per session with description for varying lengths. (minutes [b])	
													6m Outpatient phase: 8 telephone psychotherapy sessions (2 sessions per month the first two mths after discharge and one session/mth for 4 months.		
	Cognitive-behavioral therapy	Diet and exercise	Yes	Nutrition Edu.	Psychologist/ Counsellor; Dietitian; Exercise physiologist	Unclear	Individual and Group	Face to Face; Telephone	Inpatient; Home	7	7	44	Inpatient phase (2 weekly nutrition education sessions, 5 weekly physical activity classes, 2 weekly psychotherapy sessions); Outpatient phase: 8 telephone psychotherapy sessions (2 sessions per month the first two mths after discharge and one session/mth for 4 months.	45 min nutrition education; 45 min psychotherapy sessions; Telephone call length not stated.	Yes
Katzer 2008	Mail-delivered 'non-dieting' program (P3)	Diet and exercise	Yes	Nutrition Edu.; Help following programme end		No	Other – print only	Print	Home	2.3	10.3	0			No
	Group 'non-dieting' program (P2)	Diet and exercise	Yes	Nutrition Edu.; Help following programme end	Psychologist/ Counsellor; Dietitian; Health Trainer	Unclear	Group	Face to Face	Community	2.3	10.3	22	Weekly for 10 weeks, fortnightly then monthly	2 hours	No

Study ID	Groups:	Intervention type	Intervention faded in intensity	Features (meal replacements, nutrition education, financial incentives, intermittent fasting, content designed to help participants following programme end)	Provider [a]	Provider training received	Delivery		Intervention setting	Intervention timing (months)		Sessions			Intervention personalised, titrated or adapted
							Mode	Format		Last contact	End (step change in intensity)	N[c]	Frequency	Length per session with description for varying lengths. (minutes [b])	
	Group 'non-dieting' program plus Relaxation (P1)	Diet and exercise	Yes	Nutrition Edu.; Help following programme end	Psychologist/ Counsellor; Nutritionist	Unclear	Group	Face to Face; Other	Community; Home	2.3	10.3	22	Weekly for 10 weeks, fortnightly then monthly	2 hours	No
Mensinger 2016	Control, Weight Neutral Program	Diet and exercise	No	Nutrition Edu.; Help following programme end	Psychologist/ Counsellor; Personal Trainer		Group	Face to Face; Print; Other	Community	6	6	26	Weekly.	90	
	Weight Loss Program	Diet and exercise	No	Nutrition Edu.; Help following programme end			Group	Face to Face; Print; Other	Community	6	6	26	Weekly	90	
Messier 2013	Exercise only	Exercise only	Yes	Help following programme end	Personal Trainer	Yes	Unclear	Face to Face; Telephone	Community	18	6	78	3 days per week	60	Yes
	Diet-induced weight loss only	Diet only	Yes	MR-P; Nutrition Edu.; Help following programme end	Nutritionist	Yes	Individual and Group	Face to Face; Print	Community	18	6	30	1-6 Months: individual session and 3 group sessions per month; 7-18 Months: biweekly group sessions and an individual session every 2 months		Yes
	Diet-induced weight loss plus exercise	Diet and exercise	Yes	MR-P; Nutrition Edu.; Help following programme end	Nutritionist	Yes	Individual and Group	Face to Face; Telephone; Print	Community	18	6	108	1-6 Months: individual session and 3 group sessions per month; 7-18 Months: biweekly group sessions and an individual session every 2 months plus 3 days/week (exercise)	60 mins exercise; diet group sessions not reported	Yes

Study ID	Groups:	Intervention type	Intervention faded in intensity	Features (meal replacements, nutrition education, financial incentives, intermittent fasting, content designed to help participants following programme end)	Provider [a]	Provider training received	Delivery		Intervention setting	Intervention timing (months)		Sessions			Intervention personalised, titrated or adapted
							Mode	Format		Last contact	End (step change in intensity)	N[c]	Frequency	Length per session with description for varying lengths. (minutes [b])	
Munsch 2007	Group BWLT	Diet and exercise	Yes	Nutrition Edu.; Help following programme end	Psychologist/ Counsellor	Yes	Group	Face to Face	Community	16	4	22	16 weekly sessions; 6 monthly sessions - The last session took place 12 months after the end of active treatment.	90	Yes
	Group CBT	Diet only	Yes	Nutrition Edu.; Help following programme end	Psychologist/ Counsellor	Yes	Group	Face to Face	Community	16	4	22	16 weekly sessions; 6 monthly sessions - The last session took place 12 months after the end of active treatment.	90	Yes
Ng 2015	Control group	Control	No	Nutrition Edu.	Physician	No	Individual	Face to Face	Health Care	6		2	Single sessions at baseline and at 6 months		
	Lifestyle modification program	Diet and exercise	Yes	Nutrition Edu.; Help following programme end	Dietitian	No	Individual	Face to Face	Health Care	12	4	24	Weekly (Months 1-4); Monthly (Months 5-12)	Encouraged to see an exercise instructor at least once during the program and perform 30 min of aerobic exercise two to three times a week.	
Ramirez 2001	Weight control only	Diet and exercise	No	Nutrition Edu.		No	Group	Face to Face	Community	4	4	16	Weekly	1 hour	No
	Weight control plus body image therapy	Diet and exercise	No	Nutrition Edu.	Psychologist/ Counsellor; Dietitian	No	Group	Face to Face; Other	Community; Home	4	4	16	Weekly	2 hours with the psychologist or predoctoral psychology graduate student, and 1 hour with the dietitian; 12 weeks with 2 hour	No

Study ID	Groups:	Intervention type	Intervention faded in intensity	Features (meal replacements, nutrition education, financial incentives, intermittent fasting, content designed to help participants following programme end)	Provider [a]	Provider training received	Delivery		Intervention setting	Intervention timing (months)		Sessions			Intervention personalised, titrated or adapted
							Mode	Format		Last contact	End (step change in intensity)	N[c]	Frequency	Length per session with description for varying lengths. (minutes [b])	
														sessions and 4 weeks with 1 hour sessions.	
Schubel 2016	Control group	Control	Yes	Nutrition Edu.	Dietitian; Nutritionist	Yes	Individual	Face to Face; Telephone	Community	11.5	3	8	Biweekly phone calls (Week 1-12); Two single sessions at the beginning and end of the Intervention phase	"The number of personal contacts and counseling sessions was the same for all study participants overall, but individuals in the ICR and CCR arms received longer and more comprehensive counseling sessions with personalized dietary plans, specific for the ICR or CCR regimens."	No
	Continuous Calorie Restriction	Diet only	Yes	Nutrition Edu.; Help following programme end	Dietitian; Nutritionist	Yes	Individual	Face to Face; Telephone; Print	Community	11.5	3	8	Biweekly phone calls (Week 1-12); Two single sessions at the beginning and end of the Intervention phase	"The number of personal contacts and counseling sessions was the same for all study participants overall, but individuals in the ICR and CCR arms received longer and more comprehensive counseling sessions with	Yes

Study ID	Groups:	Intervention type	Intervention faded in intensity	Features (meal replacements, nutrition education, financial incentives, intermittent fasting, content designed to help participants following programme end)	Provider [a]	Provider training received	Delivery		Intervention setting	Intervention timing (months)		Sessions			Intervention personalised, titrated or adapted
							Mode	Format		Last contact	End (step change in intensity)	N[c]	Frequency	Length per session with description for varying lengths. (minutes [b])	
														personalized dietary plans, specific for the ICR or CCR regimens."	
	Intermittent Calorie Restriction	Diet only	Yes	Nutrition Edu.; Inter. Fasting; Help following programme end	Dietitian; Nutritionist	Yes	Individual	Face to Face; Telephone; Print	Community	11.5	3	8	Biweekly phone calls (Week 1-12); Two single sessions at the beginning and end of the Intervention phase	"The number of personal contacts and counseling sessions was the same for all study participants overall, but individuals in the ICR and CCR arms received longer and more comprehensive counseling sessions with personalized dietary plans, specific for the ICR or CCR regimens."	Yes
Silva 2010	Comparison group		No	Nutrition Edu.	Psychologist/ Counsellor; Dietitian; Nutritionist; Exercise physiologist	Unclear	Group	Face to Face	Community	12	12	29			
	Intervention	Diet and exercise	Yes	Nutrition Edu.; Help following programme end	Psychologist/ Counsellor; Dietitian; Nutritionist; Exercise physiologist	Unclear	Group	Face to Face; Print	Community	12	12	30	Weekly or twice a month	120	

Study ID	Groups:	Intervention type	Intervention faded in intensity	Features (meal replacements, nutrition education, financial incentives, intermittent fasting, content designed to help participants following programme end)	Provider [a]	Provider training received	Delivery		Intervention setting	Intervention timing (months)		Sessions			Intervention personalised, titrated or adapted
							Mode	Format		Last contact	End (step change in intensity)	N[c]	Frequency	Length per session with description for varying lengths. (minutes [b])	
Snel 2012	VLCD only	Diet only	No	MR-F; Nutrition Edu.		Unclear	Unclear	Face to Face	Health Care	4	4				No
	VLCD + exercise	Diet and exercise	No	MR-F; Nutrition Edu.	Physiotherapist	Unclear	Unclear	Face to Face	Health Care; Home	4	4	16	Weekly at minimum	One-hour supervised exercise sessions plus at least 4 home training sessions	No
Tapsell 2017	Usual care (Control)	Diet and exercise	Yes	Nutrition Edu.	Nurse (General)	Unclear	Individual	Face to Face; Telephone; Print	Health Care	12	3	11	Months 1-3: Monthly; Months 1 – 12: Quarterly; Phone calls: Quarterly	30 mins clinics; 15 min phone calls	Yes
	Intervention Group	Diet and exercise	Yes	Nutrition Edu.	Dietitian; Health Trainer	Yes	Individual	Face to Face; Telephone; Print	Health Care	12	3	11	Months 1-3: Monthly; Months 1 – 12: Quarterly; Phone calls: Quarterly	1 hour clinics; 15 min phone calls	Yes
	Intervention plus food supplement group (N/A)	N/A	N/A	N/A	N/A	N/A	N/A	N/A	N/A	N/A	N/A	N/A	N/A	N/A	N/A
The Look AHEAD Research Group 2010	Diabetes support and education	Diet and exercise	No	Nutrition Edu.; Help following programme end	Nurse (General); Dietitian; Health Trainer; Personal Trainer	Yes	Group	Face to Face; Telephone; Print; Other	Community		48	22	3 sessions annually for the first 4 years of follow-up; thereafter, one session was provided annually	60 – 90	No
	Intensive lifestyle intervention	Diet and exercise	Yes	MR-P; Nutrition Edu.; Help following programme end	Nurse (General); Physician; Psychologist/ Counsellor;	Yes	Individual and Group	Face to Face; Telephone; Internet; Print; Other	Community	115	12	134	Months: 1-6: weekly; Months 7-12: 3/month;	Months 1-6: Group sessions: 60 to 75 minutes; Individual	Yes

Study ID	Groups:	Intervention type	Intervention faded in intensity	Features (meal replacements, nutrition education, financial incentives, intermittent fasting, content designed to help participants following programme end)	Provider [a]	Provider training received	Delivery		Intervention setting	Intervention timing (months)		Sessions			Intervention personalised, titrated or adapted
							Mode	Format		Last contact	End (step change in intensity)	N[c]	Frequency	Length per session with description for varying lengths. (minutes [b])	
					Dietitian; Personal Trainer								Years 2-4: Minimum of 1/month;<br>Year 5+: Monthly recommended.	sessions: 20 to 30 minutes.	
vanWier 2011	Control – Brochure	Control	No	Nutrition Edu.			Other – information booklet								No
	Internet Group	Diet and exercise	No	Nutrition Edu.	Psychologist/ Counsellor; Health Trainer	Yes	Individual	Internet	Workplace; Home	6	6	10	Every 2 weeks	Work on module on internet. Email contact after completion of each module.	No
	Phone Group	Diet and exercise	No	Nutrition Edu.	Psychologist/ Counsellor; Health Trainer	Yes	Individual	Telephone	Workplace; Home	6	6	10	Every 2 weeks	Call every 2 weeks. Work on modules individually in between calls.	No
von Gruenigen 2008	Control, Usual care	Control	No	Nutrition Edu.			Unclear	Print	Health Care			0			No
	Lifestyle intervention	Diet and exercise	Yes	Nutrition Edu.; Help following programme end	Psychologist/ Counsellor; Dietitian; Other		Individual and Group	Face to Face; Telephone; Print	Health Care	6	6	24	Weekly for 6 weeks, bi-weekly for I month, and monthly for 3 months.		Yes
Wadden 1986	VLCD	Diet only	No	Nutrition Edu.	Psychologist/ Counsellor	No	Group	Face to Face		16	4	20	Weekly	90	No
	Behaviour	Diet and exercise	Yes	Nutrition Edu.; Help following programme end	Psychologist/ Counsellor	No	Group	Face to Face		24	6	35	Weekly	90	No
	Combined	Diet and exercise	No	Nutrition Edu.; Help following programme end	Psychologist/ Counsellor	No	Group	Face to Face		24	6	35	Weekly	90	No
Zwickert 2016	CBT + Minimal	Diet and exercise	Yes	Nutrition Edu.; Help following programme end	Psychologist/ Counsellor; Dietitian	No	Individual and Group	Face to Face; Telephone; SMS	Community	6	3			All sessions were 90 min in duration, with the exception of the	No

Study ID	Groups:	Intervention type	Intervention faded in intensity	Features (meal replacements, nutrition education, financial incentives, intermittent fasting, content designed to help participants following programme end)	Provider [a]	Provider training received	Delivery		Intervention setting	Intervention timing (months)		Sessions			Intervention personalised, titrated or adapted
							Mode	Format		Last contact	End (step change in intensity)	N[c]	Frequency	Length per session with description for varying lengths. (minutes [b])	
														first session which was 120 min.	
	CBT + Intensive	Diet and exercise	Yes	Nutrition Edu.; Help following programme end	Psychologist/ Counsellor; Dietitian	No	Individual and Group	Face to Face; Telephone; Internet; SMS	Community	9	3			All sessions were 90 min in duration, with the exception of the first session which was 120 min. 3-6m daily text messages, 6-9m weekly text messages	No

Approx.: Approximately; **Appt.**: Appointment/s; **Fin. Incentives;** Financial Incentives**; GP:** General Practitioner **Inter. Fasting:** Intermittent Fasting; **Min/s:** Minute/s **M/Mths:** month/s**; MR – F =** Meal replacement (Full); **MR – P =** Meal replacement (Partial); **N:** Number; **N/A:** Not applicable; **NR:** Nor reported; **Nutrition Edu.** = Nutrition Education; **PA:** Physical Activity**; SMS**: Short Message Service; **VLCD:** Very low-calorie diet

[a] See table below for Provider category descriptions [b] Unless otherwise stated; [c] Exercise sessions were assumed to be unsupervised and did not contribute to the number of sessions unless otherwise stated.

Provider	Provider descriptions as reported in included studies of parent review
Nurse (Specialist)	
Nurse (General)	Nurse educator;<br>RNS;
GP	General internists
Physician (Any doctor not a GP)	Medical doctors; Specialists in endocrinology, and internal medicine; Clinicians; Endocrinologists; Graduates in medicine; Research cardiologist; Doctoral-level clinicians (with an average of 4.8 years of experience delivering behavioral weight loss treatment); Occupational doctor.
Psychologist/ Counsellor	Therapist; Masters-level counseling psychology students; MA in behavioural psychology; Lifestyle counsellor; Graduates In psychology; Psychology graduate students; Advanced degree in behavioral psychology; Mental health counsellor; Wellness counsellors; Professional Counsellor; Psychotherapist; Psychotherapists and masters students graduate students in clinical psychology; Clinical psychology graduate students; Lifestyle counsellor; Clinical psychology graduate students; Experienced behavioural weight control counsellors; Behavior therapist; Counsellor with a degree in nutrition or physical activity
Dietitian	Dietitian; Masters of Dietetics Students
Nutritionist	Provider described by authors as nutritionist; Nutrition technician; Graduates in nutrition; Advanced degree in nutrition; Nutritional interventionist; Nutritionist (MSc in nutrition); Nutrition/Diet interventionists; Two qualified or student clinical nutritionists
Physiotherapist	Physical therapist; Physical/recreational therapists
Exercise physiologist	Exercise consultants; MA in exercise physiology; Graduates in physical activity and sport science (SPAS); Advanced degree in exercise physiology; Exercise counsellors
Other Allied Health Professionals	Occupational therapist; Pharmacist; Nurses/physician assistants; Hospital staff; Social worker with special competence in CT; Medical-assistant

Study ID	Groups:	Intervention type	Intervention faded in intensity	Features (meal replacements, nutrition education, financial incentives, intermittent fasting, content designed to help participants following programme end)	Provider [a]	Provider training received	Delivery		Intervention setting	Intervention timing (months)		Sessions			Intervention personalised, titrated or adapted
							Mode	Format		Last contact	End (step change in intensity)	N[c]	Frequency	Length per session with description for varying lengths. (minutes [b])	

Health trainer	Lifestyle coaches; Mindfulness meditation instructors; Community health educator; MA in health education; Behavioural consultant; Health educator; Telephone counsellors; Trained lifestyle coaches; Health Promotion coaches; Weight loss coaches; Wellness leader; Weight Watchers leader; Trained interventionists with expertise in both content area (i.e., physical activity and nutrition) and behavioral therapy;<br>Food advisors recruited from local community; Community Health Workers; Lifestyle activity consultant; Trained lifestyle coaches; Lifestyle Coach/ medical assistant; Masters-level staff with extensive training in behavioral weight loss; Nutrition health educator; IHM health staff graduates; 6 trained CAMWEL advisors recruited from various occupational backgrounds including healthcare, in line with the NHS health trainers initiative; Weight loss group leaders supervised by an exercise physiologist; Study coordinator (with health/nutrition background) together with a peer leader/study coordinator (experienced in adult training and self-management programs); Health educator; Degree in health sciences; Trainers (for meal replacement group); Health coach and health practitioner backgrounds and trained by the senior psychologists; Diabetes educators; EuroFIT coaches; Program providers who were trained in nutrition, education, and behavioral interventions; Masters degree–level health educators delivered health education sessions; Behaviorist;<br>Trained lay health educators (LHEs) (community volunteers or existing senior center staff); Peer health coach; Educators held an undergraduate degree in a relevant discipline (dietician, sports scientist)
Personal Trainer	Certified exercise trainer; Trained fitness instructor; Physical activity specialist; Football coaching staff; Physical Activity Counselor; Trained interventionist and exercise coaches who were skilled in exercise science; Exercise programme supervised by a professional trainer; Fitness professional; Exercise interventionists; Exercised in a supervised setting; Trained certified technicians assessed each participant;<br>Sports therapist; Exercise specialists;
Health care professional (not specified)	Church health advisors (CHAs) were members of their respective church's health ministry (e.g., nurses, pharmacists, physicians) and were trained by a co-investigator certified to perform GLB training;<br>Standard clinical care provider; Hospital based care; Primary care providers; Master's trained health professionals; health professional
Other	Research staff; Behavioral specialist; PhD-level interventionists; Doctoral level graduate students; Research assistant; Case manager; Coaches; YMCA staff; Peer leader; Teacher; Interventionist; Successful group members selected through interview; Varied, may be successful slimmers; Well-trained investigators; Research assistant; PhD holders or PhD candidates in at least their third year of study; BE WELL intervention staff; Physical activity, psychological support male researcher; The tutors; Study investigator; Ergonomist; Study coordinator; Interventionist; Cooperative Extension Service Family and Consumer Sciences Agents or individuals with bachelors or masters degrees in nutrition, exercise science, or psychology; Study partner; Trained interventionists; Group facilitator; External people representing diverse areas of expertise; Two experienced coleaders; Administrative study staff (not intervention staff); Trained graduate or undergraduate students; Had backgrounds in dietetics, psychology and/or exercise physiology; Primary investigator; Study staff
If it was an OR between providers, both were listed	

Table S6. Mental health outcome scale categories

Mental health condition	Outcome scale
Depression	1. Patient Health Questionnaire 8 (PHQ-8)<br>2. Beck Depression Inventory (BDI)<br>3. Beck Depression Inventory (BDI-IA)<br>4. Beck Depression Inventory II (BDI-II)<br>5. Hospital Anxiety and Depression Scale (HADS) - Depression<br>6. Brief Symptom Inventory 18 – Depression<br>7. Depression measured on a four-point ordinal scale<br>8. 90-item global severity index (GSI) scale - Depression dimension
Anxiety	1. Anxiety measured on a four-point ordinal scale<br>2. Beck Anxiety Inventory (English version, German version)<br>3. State-Trait Anxiety Questionnaire – State<br>4. Brief symptom inventory – Anxiety<br>5. Hospital Anxiety and Depression Scale (HADS) – Anxiety subscale<br>6. 90-item global severity index (GSI) scale – Anxiety dimension
Depression and Anxiety	1. EQ-5D 5L Anxiety/depression dimension only<br>2. EQ-5D 3L Anxiety/depression dimension only<br>3. Hospital Anxiety and Depression Scale (HADS) – Total<br>4. Depression Anxiety Stress Scale (DASS-21)<br>5. General Health Questionnaire – 12
Self-esteem	1. Rosenberg Self-Esteem Scale
Mental health composite score	1. Short Form-12 (MCS)<br>2. Rand 36-item Health Survey mental health composite score<br>3. SF-36v2 Mental Health Component<br>4. RAND 36-Item Health Survey - emotional wellbeing
Impact of weight on Quality of Life (QoL)	1. Impact of weight on QoL – Lite
Stress	1. Global Severity Index-90 (GSI-90) based on the German version of the Symptom Checklist (SCL-90R)<br>2. The 10-item Perceived Stress Scale<br>3. Global Perceived Stress Scale (STS) score (Jackson Heart study)<br>4. Perceived Stress Scale<br>5. OQ-45.2 (Italian language version)<br>6. Brief symptom inventory
Psychological wellbeing	1. Profile of Mood States Short Form scale of Total Mood Disturbance<br>2. Eight-item wellbeing questionnaire<br>3. Short Form of the positive and negative affect scale (PANAS) – Positive Affect<br>4. Short Form of the positive and negative affect scale (PANAS) – Negative Affect<br>5. Fragebogen zur Lebenszufriedenheit (FLZ)<br>6. Satisfaction with Life Scale (SWLS)
Body image	1. Multidimensional Body-Self Relations Questionnaire Appearance Scale (MBSRQ-AS)<br>2. Multidimensional Body-Self Relations Questionnaire Body Areas Satisfaction Scale (MBSRQ-BAS)<br>3. Body Image Avoidance Questionnaire (BIAQ)<br>4. Eating Disorder Examination Interview (EDE): Shape Concern<br>5. Body Shape Questionnaire<br>6. Body Dysmorphic Disorder Examination - Self-Administration<br>7. Body Image Assessment questionnaire<br>8. Physical Self-Perception Profile questionnaire - global physical self-worth scale<br>9. Physical Self-Perception Profile - body attractiveness scale
Eating disorders	1. EDE: Global score<br>2. EDE: Dietary Restraint<br>3. EDE: Weight Concern<br>4. EDE: Eating Concern<br>5. EDE: Concern subscale<br>6. Binge Eating Scale

Table S7. Sensitivity analyses

Follow-up timepoint at or after programme end	Total Studies (n)	Studies removed due to high RoB (n)	Total participants (n)	Hetero-geneity (I^2, %)	Effect estimate	P-value	Change in effect
Depression (Intervention Vs Control)							
At end	6		5518	92%	-0.34 [−0.71, 0.03]	0.07	
At end*	5	1	701	95%	-0.23 [-0.56, 0.09]	0.16	No
1-6m	8		3180	59%	-0.04 [-0.19, 0.11]	0.57	
1-6m*	7	1	1199	63%	-0.02 [-0.22, 0.19]	0.88	No
7-12m	5		5395	0%	**-0.18 [-0.23, -0.13]**	**<0.0001**	
7-12m*	4	1	713	0%	-0.07 [-0.21, 0.08]	0.37	No
19-24m	2		4901	99%	1.04 [-1.30, 3.39]	0.38	
19-24m	1	1	236	N/A	**2.24 [1.92, 2.57]**	**<0.00001**	Yes
31-36m	1		4598	N/A	-0.24 [-0.37, -0.11]	N/A	Study at high RoB
79-84m	1		4344	N/A	-0.14 [-0.27, -0.01]	N/A	Study at high RoB
Anxiety (Intervention Vs Control)							
1-6m	2		2179	27%	**-0.17 [-0.31, -0.03]**	**0.02**	
1-6m*	1	1	188	N/A	**-0.32 [-0.61, -0.02]**	**N/A**	No
Depression and Anxiety (Intervention Vs Control)							
At end	4		1017	31%	-0.02 [-0.18, 0.14]	0.81	
At end*	3	1	951	0%	0.03 [-0.10, 0.16]	0.64	Yes: change in direction of effect; 95% CI overlap; No heterogeneity
7-12m	4		887	77%	-0.11 [-0.45, 0.22]	0.50	
7-12m*	3	1	844	0%	0.09 [-0.06, 0.23]	0.24	Yes: change in direction of effect; 95% CI overlap; No heterogeneity
Self-esteem (Intervention Vs Control)							
At end	2		836	39%	**0.50 [0.29, 0.71]**	**<0.0001**	
At end*	2	1	162	N/A	**0.34 [0.03, 0.66]**	**N/A**	No
7-12m	1		667	N/A	0.20 [0.14, 0.26]	N/A	
7-12m*	1	1	Not estimable				N/A
Mental health composite score (Intervention Vs Control)							
At end	11		7352	94%	**0.36 [0.10, 0.62]**	**0.007**	
At end*	8	3	1717	96%	**0.52 [0.02, 1.02]**	**0.04**	No
1-6m	4		2909	90%	0.16 [-0.13, 0.46]	0.28	
1-6m*	3	1	874	93%	0.22 [-0.32, 0.75]	0.43	No
7-12m	11		6951	91%	**0.24 [0.01, 0.47]**	**0.04**	

7-12m*	8	3	1482	93%	0.41 [-0.02, 0.85]	0.09	Substantial unaccountable heterogeneity observed in pooled estimates.
19-24m	3		4946	97%	-0.36 [-1.10, 0.47]	0.39	
19-24m*	2	1	277	97%	-0.53 [-2.06, 1.01]	0.50	No
31-36m	1		4594	N/A	-0.11 [-0.27, 0.05]	N/A	Study at high RoB
48-54m	1		4503	N/A	0.04 [-0.18, 0.26]	N/A	Study at high RoB
55-60m	1		4464	N/A	0.20 [-0.03, 0.43]	N/A	Study at high RoB
67-72m	1		4410	N/A	0.04 [-0.11, 0.19]	N/A	Study at high RoB
79-84m	1		4364	N/A	0.25 [0.01, 0.49]	N/A	Study at high RoB
91-96m	1		3565	N/A	0.41 [0.17, 0.65]	N/A	Study at high RoB
103-108m	1		1917	N/A	0.06 [-0.25, 0.37]	N/A	Study at high RoB
Mental health composite score (Diet and exercise intervention Vs Diet only comparator)							
At end	2		437	0%	**0.31 [0.12, 0.50]**	**0.001**	
At end*	1	1	261	N/A	**0.26 [0.02, 0.51]**	**0.03**	No
Psychological wellbeing (Intervention Vs Control)							
At end	2		738	0%	**0.22 [0.07, 0.36]**	**0.003**	Both studies at high risk of bias
At end*	2		373	52%	**0.52 [0.18, 0.87]**	**0.003**	Both studies at high risk of bias
7-12m*	2		710	92%	0.79 [-0.42, 1.99]	0.20	Both studies at high risk of bias
7-12m	2		710	90%	0.83 [-0.27, 1.94]	0.14	Both studies at high risk of bias
Two larger studies at high risk of bias were removed for sensitivity analyses resulting in a notable drop in total participants for many meta-analyses reported above. Full justification for high risk of bias ratings for these two studies can be found in Table S2; both were judged to be at high risk of other bias. In one case, this was because of a modified open-label intervention after initial study end, for the other, this was because intervention participants who did not lose 10% of their initial weight during the first 6 months were offered an alternative intervention.							

SUPPLEMENTARY FIGURES

Figure S1. PRISMA diagram of study flow

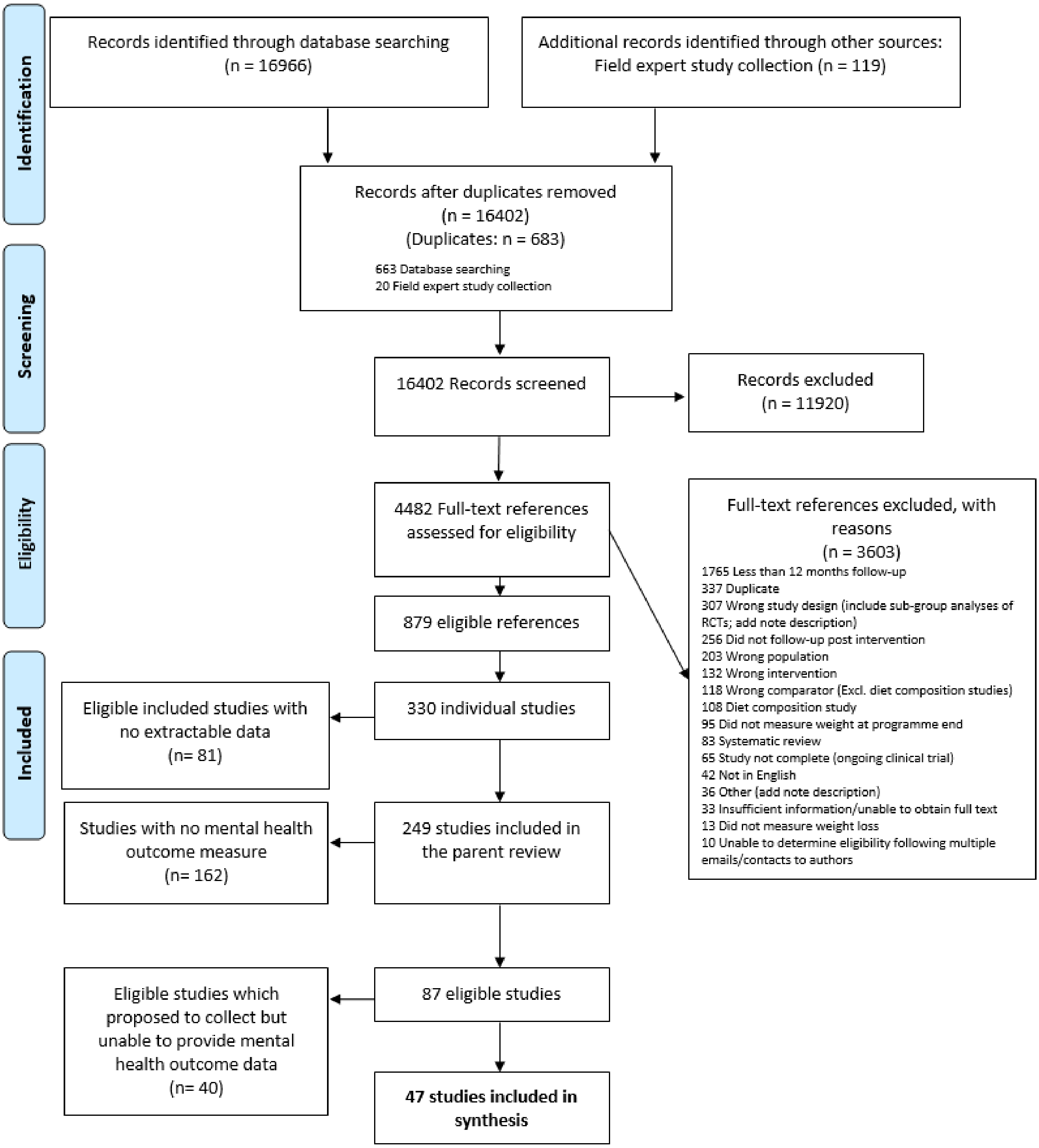

Depression

Outcome measures:

1. Beck Depression Inventory (BDI)
2. Beck Depression Inventory (BDI-IA)
3. Beck Depression Inventory II (BDI-II)
4. Brief Symptom Inventory 18 – Depression
5. Depression measured on a four-point ordinal scale
6. Hospital Anxiety and Depression Scale (HADS) - Depression
7. Patient Health Questionnaire 8 (PHQ-8)
8. 90-item global severity index (GSI) scale - Depression dimension

Direction of mental health scale: higher score = more severe depressive symptoms

Comparison 1 – BWMP (diet and/or exercise) versus Control group 1-4

Figure S2a. Depression at programme end

Study or Subgroup	Intervention Mean	Intervention SD	Intervention Total	Control Mean	Control SD	Control Total	Weight	Std. Mean Difference IV, Random, 95% CI
**1.1.1 Control group 1**								
-2.5 Grilo 2014	-7.2	3.55	23	-4	2.76	22	12.7%	-0.99 [-1.61, -0.36]
-1.0 Bennett 2013	-2.33	5.23	93	-0.36	4.06	90	17.7%	-0.42 [-0.71, -0.13]
+0.8 Bennett 2012	-1.47	4.29	114	-1.19	4.42	139	18.2%	-0.06 [-0.31, 0.18]
**Subtotal (95% CI)**			**230**			**251**	**48.6%**	**-0.41 [-0.83, 0.01]**
Heterogeneity: Tau² = 0.10; Chi² = 8.72, df = 2 (P = 0.01); I² = 77%								
Test for overall effect: Z = 1.89 (P = 0.06)								
**1.1.2 Control group 3**								
-7.9 The Look AHEAD Research Group 2010 (1)	-1.41	1.39	2435	-0.41	1.2	2382	19.7%	-0.77 [-0.83, -0.71]
+0.8 deZwaan 2017	-4.1	3.32	85	-3.2	3.15	77	17.4%	-0.28 [-0.59, 0.03]
+0.7 Barnes 2017	-1.73	2.32	29	-2.8	2.11	29	14.2%	0.48 [-0.05, 1.00]
**Subtotal (95% CI)**			**2549**			**2488**	**51.4%**	**-0.24 [-0.86, 0.38]**
Heterogeneity: Tau² = 0.27; Chi² = 30.37, df = 2 (P < 0.00001); I² = 93%								
Test for overall effect: Z = 0.75 (P = 0.45)								
**Total (95% CI)**			**2779**			**2739**	**100.0%**	**-0.34 [-0.71, 0.03]**
Heterogeneity: Tau² = 0.18; Chi² = 61.70, df = 5 (P < 0.00001); I² = 92%								
Test for overall effect: Z = 1.81 (P = 0.07)								
Test for subgroup differences: Chi² = 0.20, df = 1 (P = 0.66), I² = 0%								

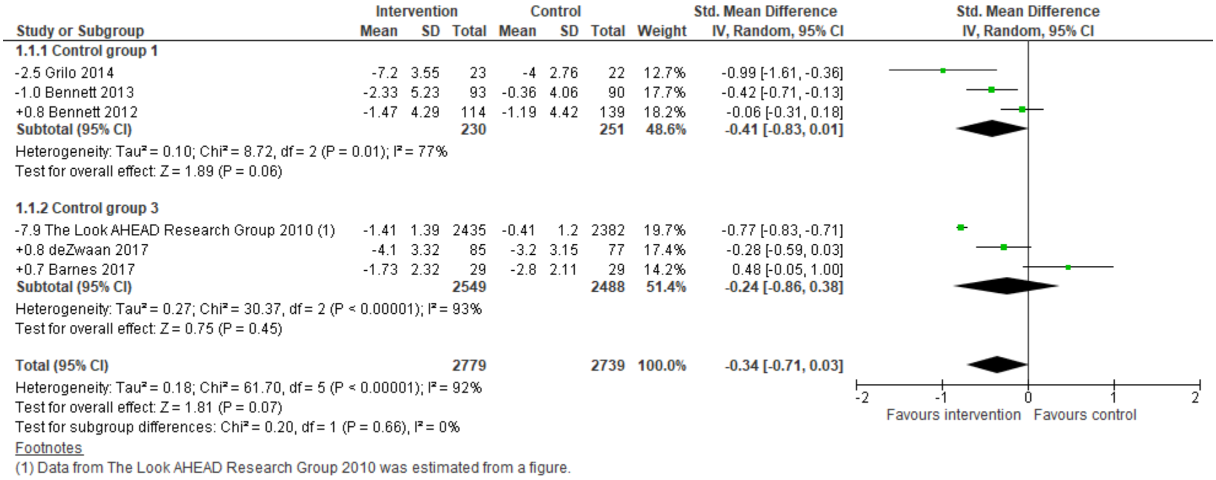

Footnotes
(1) Data from The Look AHEAD Research Group 2010 was estimated from a figure.

Figure S2b. Depression at 1-6 months after programme end

Study or Subgroup	Intervention Mean	Intervention SD	Intervention Total	Control Mean	Control SD	Control Total	Weight	Std. Mean Difference IV, Random, 95% CI
**1.2.1 Control group 1**								
-8.2 Foster-Schubert 2012	-2.1	8.2	108	0.4	9.6	80	12.2%	-0.28 [-0.57, 0.01]
-4.4 Foley 2016	-0.32	4.48	153	-0.42	4.82	155	15.1%	0.02 [-0.20, 0.24]
-1.7 Bennett 2013	-2.18	4.79	85	-0.35	4.73	88	11.8%	-0.38 [-0.68, -0.08]
-1.0 Bennett 2012	-1.26	4.19	112	-1.21	4.31	132	13.8%	-0.01 [-0.26, 0.24]
-0.7 Grilo 2014	-6.7	5.52	23	-4.9	4.15	22	5.0%	-0.36 [-0.95, 0.23]
**Subtotal (95% CI)**			**481**			**477**	**58.0%**	**-0.16 [-0.34, 0.02]**
Heterogeneity: Tau² = 0.02; Chi² = 6.96, df = 4 (P = 0.14); I² = 43%								
Test for overall effect: Z = 1.78 (P = 0.07)								
**1.2.2 Control group 3**								
-4.4 vonGruenigen 2008	-1.1	2.02	17	-2.3	2.49	18	4.0%	0.52 [-0.16, 1.19]
-0.5 deZwaan 2017	-4.1	4.75	80	-4.9	4.37	70	11.1%	0.17 [-0.15, 0.50]
+1.4 Barnes 2017	-0.73	3.49	27	-2.5	2.16	29	5.7%	0.61 [0.07, 1.14]
**Subtotal (95% CI)**			**124**			**117**	**20.8%**	**0.33 [0.06, 0.61]**
Heterogeneity: Tau² = 0.01; Chi² = 2.21, df = 2 (P = 0.33); I² = 9%								
Test for overall effect: Z = 2.36 (P = 0.02)								
**1.2.3 Control group 4**								
-6.4 Diabetes Prevention Program R G 2009	-1.02	4.12	988	-0.58	4.52	993	21.2%	-0.10 [-0.19, -0.01]
**Subtotal (95% CI)**			**988**			**993**	**21.2%**	**-0.10 [-0.19, -0.01]**
Heterogeneity: Not applicable								
Test for overall effect: Z = 2.26 (P = 0.02)								
**Total (95% CI)**			**1593**			**1587**	**100.0%**	**-0.04 [-0.19, 0.11]**
Heterogeneity: Tau² = 0.03; Chi² = 19.60, df = 8 (P = 0.01); I² = 59%								
Test for overall effect: Z = 0.57 (P = 0.57)								
Test for subgroup differences: Chi² = 9.60, df = 2 (P = 0.008), I² = 79.2%								

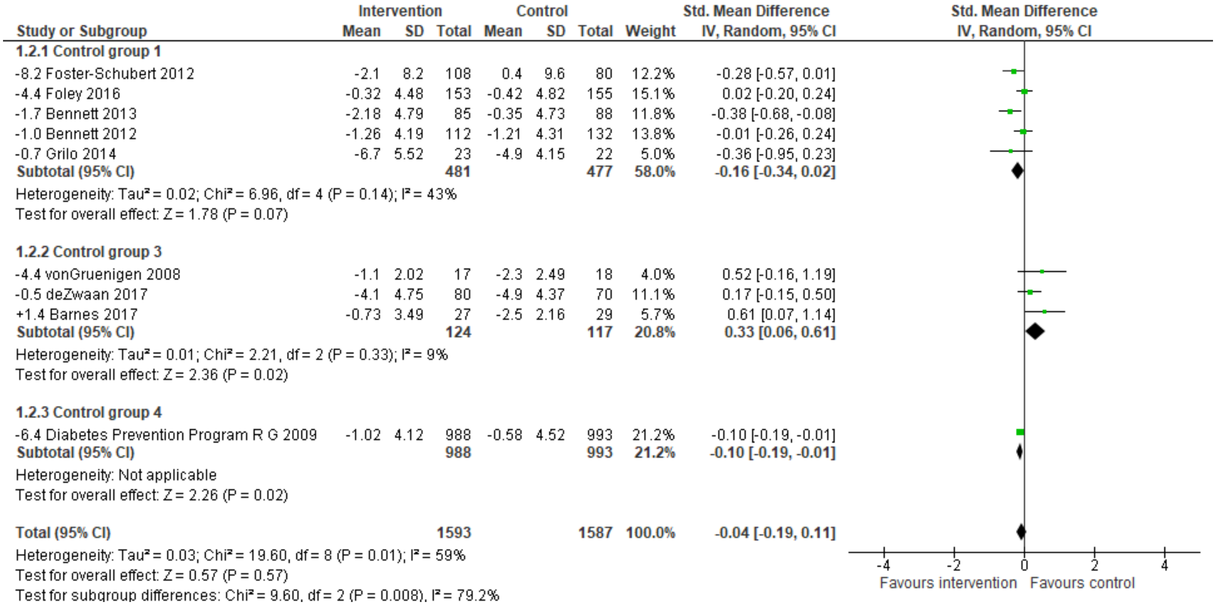

Figure S2c. Depression at 7-12 months after programme end

Study or Subgroup	Intervention Mean	Intervention SD	Intervention Total	Control Mean	Control SD	Control Total	Weight	Std. Mean Difference IV, Random, 95% CI
**1.3.1 Control group 1**								
-4.8 Grilo 2014	-6.5	5.62	21	-6.1	5.41	23	0.8%	-0.07 [-0.66, 0.52]
-3.9 Foley 2016	-1.22	4.09	155	-1.21	5.31	159	5.8%	-0.00 [-0.22, 0.22]
-1.0 Bennett 2012	-1.68	5.45	142	-0.93	4.85	164	5.7%	-0.15 [-0.37, 0.08]
**Subtotal (95% CI)**			**318**			**346**	**12.3%**	**-0.07 [-0.22, 0.08]**
Heterogeneity: Tau² = 0.00; Chi² = 0.80, df = 2 (P = 0.67); I² = 0%								
Test for overall effect: Z = 0.93 (P = 0.35)								
**1.3.2 Control group 3**								
-5.4 The Look AHEAD Research Group 2010 (1)	-0.78	2.5	2365	-0.34	1.9	2317	86.8%	-0.20 [-0.26, -0.14]
+2.9 Barnes 2017	-1.13	4.69	23	-1.19	5.71	26	0.9%	0.01 [-0.55, 0.57]
**Subtotal (95% CI)**			**2388**			**2343**	**87.7%**	**-0.20 [-0.25, -0.14]**
Heterogeneity: Tau² = 0.00; Chi² = 0.53, df = 1 (P = 0.47); I² = 0%								
Test for overall effect: Z = 6.71 (P < 0.00001)								
**Total (95% CI)**			**2706**			**2689**	**100.0%**	**-0.18 [-0.23, -0.13]**
Heterogeneity: Tau² = 0.00; Chi² = 3.52, df = 4 (P = 0.47); I² = 0%								
Test for overall effect: Z = 6.61 (P < 0.00001)								
Test for subgroup differences: Chi² = 2.20, df = 1 (P = 0.14), I² = 54.5%								

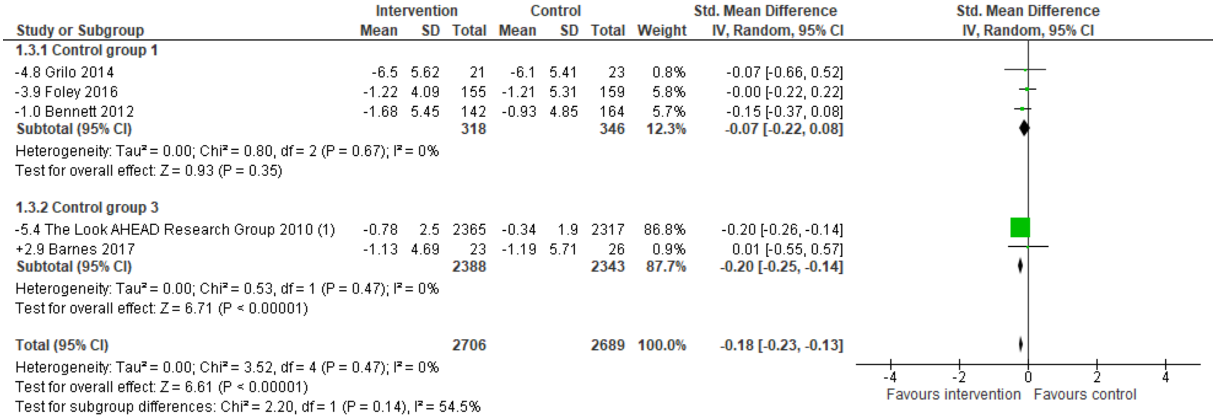

Footnotes
(1) Data from The Look AHEAD Research Group 2010 was estimated from a figure.

Figure S2d. Depression at 19-24 months after programme end

Considerable heterogeneity unexplained by weight change differences.

Study or Subgroup	Intervention Mean	Intervention SD	Intervention Total	Control Mean	Control SD	Control Total	Weight	Std. Mean Difference IV, Random, 95% CI
**1.4.1 Control group 2**								
-4.3 Appel 2011	0.25	0.32	125	-0.47	0.32	111	49.8%	2.24 [1.92, 2.57]
**Subtotal (95% CI)**			**125**			**111**	**49.8%**	**2.24 [1.92, 2.57]**
Heterogeneity: Not applicable								
Test for overall effect: Z = 13.44 (P < 0.00001)								
**1.4.2 Control group 3**								
-4.2 The Look AHEAD Research Group 2010 (1)	-0.72	2.45	2341	-0.39	1.9	2324	50.2%	-0.15 [-0.21, -0.09]
**Subtotal (95% CI)**			**2341**			**2324**	**50.2%**	**-0.15 [-0.21, -0.09]**
Heterogeneity: Not applicable								
Test for overall effect: Z = 5.13 (P < 0.00001)								
**Total (95% CI)**			**2466**			**2435**	**100.0%**	**1.04 [-1.30, 3.39]**
Heterogeneity: Tau² = 2.85; Chi² = 199.52, df = 1 (P < 0.00001); I² = 99%								
Test for overall effect: Z = 0.87 (P = 0.38)								
Test for subgroup differences: Chi² = 199.52, df = 1 (P < 0.00001), I² = 99.5%								

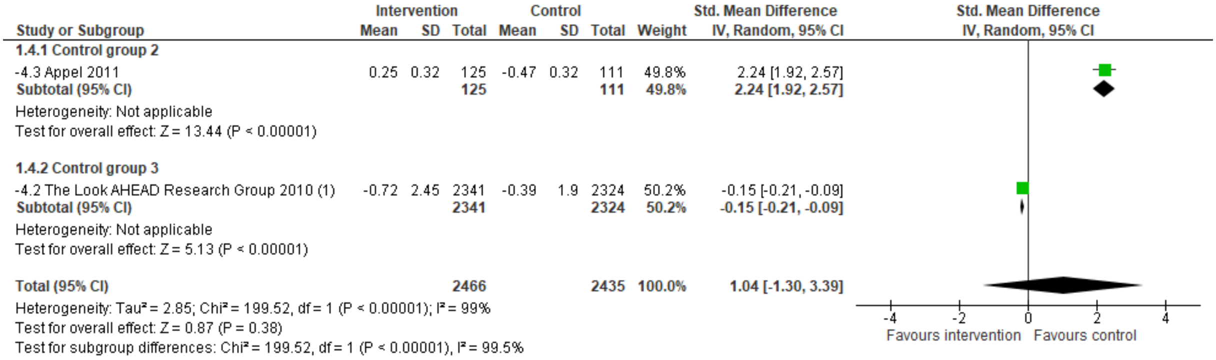

Footnotes
(1) Data from The Look AHEAD Research Group 2010 was estimated from a figure.

Figure S2e. Depression at 31-36 months after programme end

Study or Subgroup	Intervention Mean	Intervention SD	Intervention Total	Control Mean	Control SD	Control Total	Mean Difference IV, Random, 95% CI
-3.6 The Look AHEAD Research Group 2010 (1)	-0.56	2.57	2314	-0.32	1.93	2284	-0.24 [-0.37, -0.11]

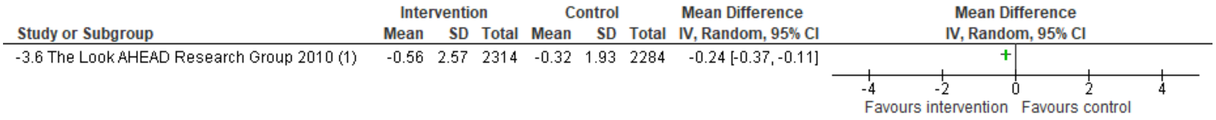

Footnotes
(1) Data from The Look AHEAD Research Group 2010 was estimated from a figure.

Figure S2f. Depression at 79-84 months after programme end

Study or Subgroup	Intervention Mean	Intervention SD	Intervention Total	Control Mean	Control SD	Control Total	Mean Difference IV, Random, 95% CI
-2.0 The Look AHEAD Research Group 2010 (1)	-0.41	2.47	2195	-0.27	1.88	2149	-0.14 [-0.27, -0.01]

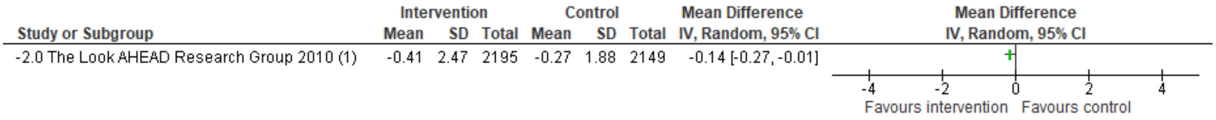

Footnotes
(1) Data from The Look AHEAD Research Group 2010 was estimated from a figure.

Comparison 2 – BWMP (diet and exercise) versus diet only

Figure S2g. Depression at programme end

Considerable heterogeneity unexplained by weight change differences.

Study or Subgroup	Diet and Exercise Mean	Diet and Exercise SD	Diet and Exercise Total	Diet only Mean	Diet only SD	Diet only Total	Weight	Std. Mean Difference IV, Random, 95% CI
-9.1 Grilo 2011	-4.8	2.36	31	-5.1	2.69	34	36.2%	0.12 [-0.37, 0.60]
-5.2 Wadden 1986 (1)	-9.12	5.35	15	-2.21	2.92	17	31.5%	-1.59 [-2.40, -0.78]
-4 Snel 2012	-2	3.61	13	-2	0.92	14	32.3%	0.00 [-0.75, 0.75]
**Total (95% CI)**			**59**			**65**	**100.0%**	**-0.46 [-1.46, 0.54]**

Heterogeneity: Tau² = 0.66; Chi² = 13.15, df = 2 (P = 0.001); I² = 85%
Test for overall effect: Z = 0.90 (P = 0.37)

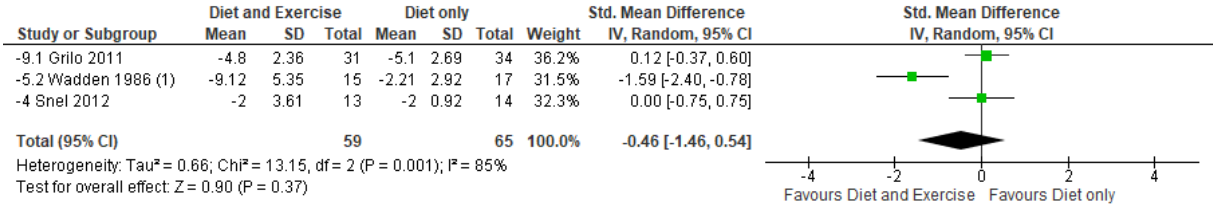

Footnotes
(1) Number of participants (N) not available; N imputed using data at closest follow-up time point

Figure S2h. Depression at 1-6 months after programme end

Considerable heterogeneity unexplained by weight change differences.

Study or Subgroup	Diet and exercise Mean	Diet and exercise SD	Diet and exercise Total	Diety only Mean	Diety only SD	Diety only Total	Weight	Std. Mean Difference IV, Random, 95% CI
-3.1 Grilo 2011	-4.8	4.2	39	-7.1	2.69	37	46.8%	0.64 [0.18, 1.10]
-1.8 Foster-Schubert 2012	-2.1	4.12	108	-1.6	4.66	105	53.2%	-0.11 [-0.38, 0.16]
**Total (95% CI)**			**147**			**142**	**100.0%**	**0.24 [-0.50, 0.98]**

Heterogeneity: Tau² = 0.25; Chi² = 7.67, df = 1 (P = 0.006); I² = 87%
Test for overall effect: Z = 0.64 (P = 0.52)

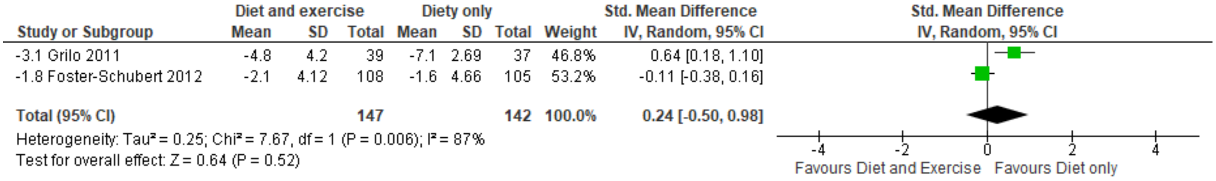

Figure S2i. Depression at 7-12 months after programme end

Study or Subgroup	Diet and Exercise Mean	Diet and Exercise SD	Diet and Exercise Total	Diet only Mean	Diet only SD	Diet only Total	Mean Difference IV, Random, 95% CI
Wadden 1986	-8.51	5.85	17	-0.89	3.73	15	-7.62 [-10.98, -4.26]

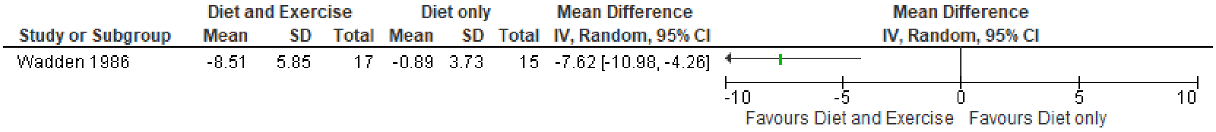

Figure S2j. Depression at 13-18 months after programme end

Study or Subgroup	Intervention Mean	Intervention SD	Intervention Total	Control Mean	Control SD	Control Total	Weight	Std. Mean Difference IV, Random, 95% CI
-6 Snel 2012	-1	1.77	13	0	1.4	14	33.3%	-0.61 [-1.39, 0.16]
-2.5 Grilo 2011	-6.3	4	37	-6.1	2.94	37	66.7%	-0.06 [-0.51, 0.40]
**Total (95% CI)**			**50**			**51**	**100.0%**	**-0.24 [-0.75, 0.27]**

Heterogeneity: Tau² = 0.05; Chi² = 1.46, df = 1 (P = 0.23); I² = 31%
Test for overall effect: Z = 0.92 (P = 0.36)

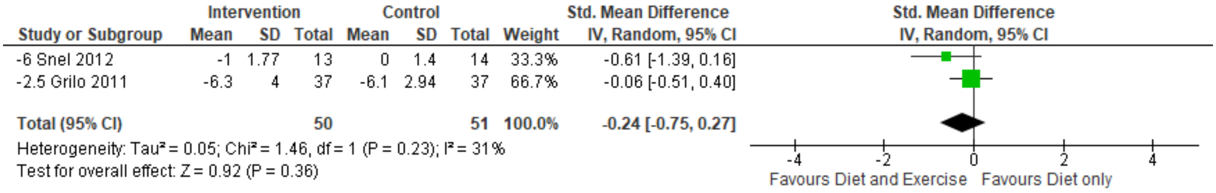

Figure S2k. Depression at 31-36 months after programme end

Study or Subgroup	Intervention Mean	Intervention SD	Intervention Total	Control Mean	Control SD	Control Total	Mean Difference IV, Random, 95% CI
-2.3 Wadden 1986 (1)	-3.06	4.88	17	-0.54	3.01	15	-2.52 [-5.30, 0.26]

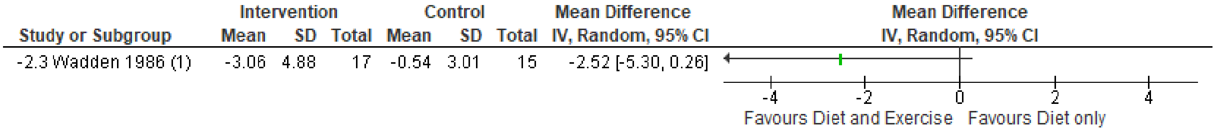

Footnotes
(1) Number of participants (N) not available; N imputed using data at closest follow-up time point

Comparison 3 – BWMP (diet and exercise) versus exercise only

No studies

Comparison 4 – Intervention Vs Comparator intervention

Depression head-to-head intervention comparisons

At programme end, seven studies all had 95% CIs that included no difference in depression scores between intervention and comparator groups, however the direction of effect seemed to favour the study group that had the greatest reduction in weight (Figure S2l.); one of these studies was at high risk of bias.[1] One study[2] favoured the comparator arm (SMD 0.91 [0.27, 1.55]; n= 42) which also had the small yet greater reduction in weight. Another study[3] favoured the intervention group (-1.43 [-2.02, -0.84]; n = 57) despite a greater reduction in weight in the comparator group.

At 1-6 months after programme end, most studies again showed no difference in change in depression scores between intervention and comparator (95% CIs included no difference), one study[1] of which was at high risk of bias. One study group[4] comparison favoured the intervention (SMD -0.41 [-0.80, -0.01]; n = 109), despite greater weight loss in the comparator group (Figure S2m).

At 7-12 months, the direction of effect appeared to mostly favour the group with greatest reduction in weight (Figure S2n); one study[1] showing no difference was at high risk of bias. At 13-18 and 37-42 months after programme end, one study[5] found no difference in change in depression scores, with the direction of effect favouring the intervention however 95% CIs included the possibility of favouring the comparator (Figures 2So, 2Sq). At 31-36 months, another single, small study[6] favoured the intervention group (MD -3.67 [-6.93, -0.41]; n = 33), which also had the greatest reduction in weight (Figure S2p).

Figure S2l. Depression at programme end

Study or Subgroup	Intervention Mean	Intervention SD	Intervention Total	Comparator Mean	Comparator SD	Comparator Total	Std. Mean Difference IV, Random, 95% CI
-5.1 Bacon 2002 (1)	-5.7	5.31	23	-3.6	2.8	36	-0.52 [-1.06, 0.01]
-5.0 Wadden 1986 (2)	-9.12	5.35	17	-6.35	1.88	16	-0.67 [-1.37, 0.04]
-2.6 Forman 2016 (3)	-0.93	1.77	78	-0.61	2.02	72	-0.17 [-0.49, 0.15]
-0.4 Andersen 1999 (4)	-3.8	2.74	19	-4.9	2.19	19	0.43 [-0.21, 1.08]
+2.3 Munsch 2007 (5)	-5.98	2.75	31	-2.63	1.63	26	-1.43 [-2.02, -0.84]
+0.8 Katzer 2008b (6)	-0.17	0.5	48	-0.09	0.4	81	-0.18 [-0.54, 0.18]
+0.7 Ames 2005 (7)	-5.7	4.11	13	-8.5	4.94	13	0.60 [-0.19, 1.39]
+0.4 Cleo 2018 (8)	0.4	2.48	22	-1.9	2.46	20	0.91 [0.27, 1.55]
+0.1 Katzer 2008a (9)	-0.22	0.5	53	-0.09	0.4	81	-0.29 [-0.64, 0.06]

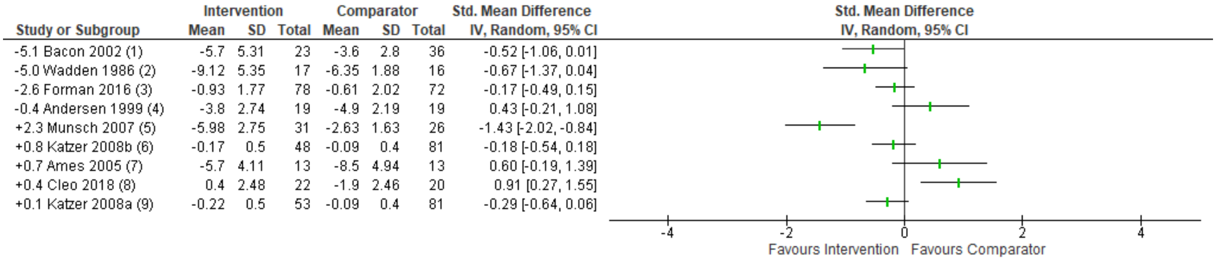

Footnotes
(1) Diet program Vs Non-diet wellness program
(2) Very low calorie diet plus behavior therapy combined Vs Behaviour therapy alone
(3) Acceptance-Based Treatment Vs Standard Behavioural Treatment; Estimated 100kg baseline weight for each group to calculate weight change difference
(4) Diet plus Structured Aerobic Exercise Vs Diet and Lifestyle Activity
(5) Cognitive-behavioural therapy Vs Behavioural weight loss treatment
(6) Group 'non-dieting' program (P2) Vs Mail-delivered 'non-dieting' program (P3); Same study as Katzer 2008a but different intervention group
(7) Reformulated cognitive-behavioural treatment Vs Standard behavioural treatment
(8) DSD Do Something Different Vs The Ten Top Tips
(9) Group 'non-dieting' program based on Relaxation Response Training (P1) Vs Mail-delivered 'non-dieting' program (P3); Same study as Katzer 2008b but different intervention group

Figure S2m. Depression at 1-6 months after programme end

Study or Subgroup	Intervention Mean	Intervention SD	Intervention Total	Comparator Mean	Comparator SD	Comparator Total	Std. Mean Difference IV, Random, 95% CI
-6.2 Bacon 2002 (1)	-5.5	5.27	23	-3.7	3.29	29	-0.41 [-0.97, 0.14]
-3.5 Forman 2016 (2)	-3.49	2.9	64	-2.98	2.63	48	-0.18 [-0.56, 0.19]
-0.2 Ames 2005 (3)	-4.8	3.38	13	-5.8	5.24	13	0.22 [-0.55, 0.99]
+2.5 Katzer 2008b (4)	-0.17	0.5	39	-0.1	0.4	68	-0.16 [-0.55, 0.24]
+1.8 Katzer 2008a (5)	-0.28	0.5	41	-0.1	0.4	68	-0.41 [-0.80, -0.01]

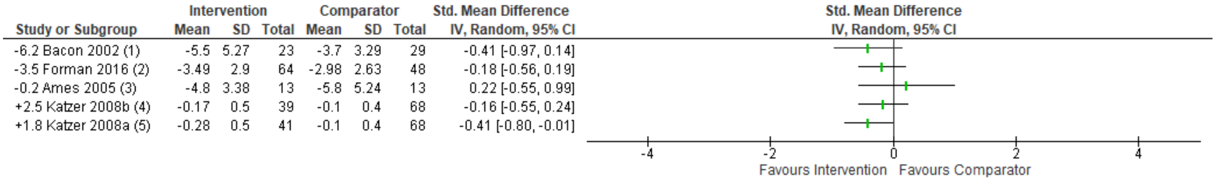

Footnotes
(1) Diet program Vs Non-diet wellness program
(2) Acceptance-Based Treatment Vs Standard Behavioural Treatment; Estimated 100kg baseline weight for each group to calculate weight change difference
(3) Reformulated cognitive-behavioural treatment Vs Standard behavioural treatment
(4) Group 'non-dieting' program (P2) Vs Mail-delivered 'non-dieting' program (P3); Same study as Katzer 2008a but different intervention group
(5) Group 'non-dieting' program based on Relaxation Response Training (P1) Vs Mail-delivered 'non-dieting' program (P3); Same study as Katzer 2008b but different intervention group

Figure S2n. Depression at 7-12 months after programme end

Study or Subgroup	Intervention group Mean	Intervention group SD	Intervention group Total	Comparator group Mean	Comparator group SD	Comparator group Total	Std. Mean Difference IV, Random, 95% CI
-6.2 Bacon 2002 (1)	-5.5	5.27	23	-3.7	3.29	29	-0.41 [-0.97, 0.14]
-3.6 Munsch 2007 (2)	-6.91	5.43	22	-4.06	2.48	21	-0.66 [-1.27, -0.04]
-3.4 Wadden 1986 (3)	-8.51	5.85	17	-5.18	3.31	16	-0.68 [-1.38, 0.03]
-0.6 Katzer 2008a (4)	-0.41	0.5	36	-0.06	0.4	63	-0.79 [-1.22, -0.37]
-0.2 Ames 2005 (5)	-4.8	3.38	13	-5.8	5.24	13	0.22 [-0.55, 0.99]
+1.5 Katzer 2008b (6)	-0.22	0.4	38	-0.06	0.4	63	-0.40 [-0.80, 0.01]
+1.1 Cleo 2018 (7)	-1.7	3.95	22	-2.6	3.19	21	0.25 [-0.35, 0.85]
+1.1 Andersen 1999 (8)	-4.2	2.62	17	-5	3.55	16	0.25 [-0.43, 0.94]

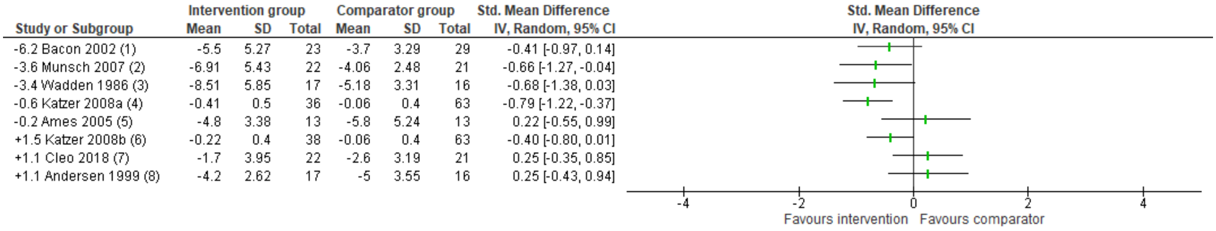

Footnotes
(1) Diet program Vs Non-diet wellness program
(2) Cognitive-behavioural therapy Vs Behavioural weight loss treatment
(3) Very low calorie diet plus behavior therapy combined Vs Behaviour therapy alone
(4) Group 'non-dieting' program based on Relaxation Response Training (P1) Vs Mail-delivered 'non-dieting' program (P3); Same study as Katzer 2008b but different intervention group
(5) Reformulated cognitive-behavioural treatment Vs Standard behavioural treatment
(6) Group 'non-dieting' program (P2) Vs Mail-delivered 'non-dieting' program (P3); Same study as Katzer 2008a but different intervention group
(7) DSD Do Something Different Vs The Ten Top Tips
(8) Diet plus Structured Aerobic Exercise Vs Diet and Lifestyle Activity

Figure S2o. Depression at 13 -18 months after programme end

Study or Subgroup	Intervention Mean	Intervention SD	Intervention Total	Comparator Mean	Comparator SD	Comparator Total	Std. Mean Difference IV, Random, 95% CI
-1.9 Forman 2016 (1)	-0.69	3.2	75	-0.31	3.15	67	-0.12 [-0.45, 0.21]

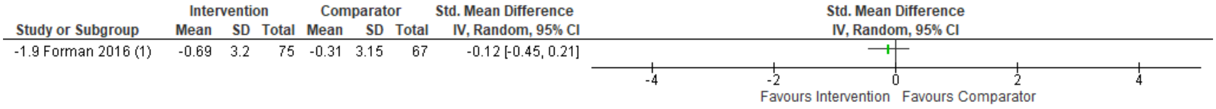

Footnotes
(1) Acceptance-Based Treatment Vs Standard Behavioural Treatment; Estimated 100kg baseline weight for each group to calculate weight change difference between study groups

Figure S2p. Depression at 31 -36 months after programme end

Study or Subgroup	Intervention group Mean	SD	Total	Comparator group Mean	SD	Total	Mean Difference IV, Random, 95% CI
-1.8 Wadden 1986 (1)	-3.06	4.88	17	0.61	4.67	16	-3.67 [-6.93, -0.41]

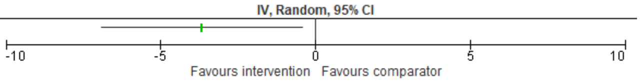

Footnotes
(1) Very low calorie diet plus behavior therapy combined Vs Behaviour therapy alone; Number of participants (N) at follow-up not available - imputed using N at next closest follow-up time point

Figure S2q. Depression at 37 -42 months after programme end

Study or Subgroup	Intervention Mean	SD	Total	Comparator Mean	SD	Total	Mean Difference IV, Random, 95% CI
-1.4 Forman 2016 (1)	-0.37	3.15	70	-0.07	3.2	62	-0.30 [-1.39, 0.79]

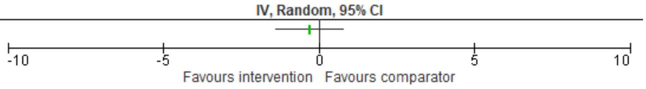

Footnotes
(1) Acceptance-Based Treatment Vs Standard Behavioural Treatment; Estimated 100kg baseline weight for each group to calculate weight change difference between study groups

Anxiety

Outcome measures:

1. Anxiety measured on a four-point ordinal scale
2. Beck Anxiety Inventory (English version; German version)
3. Brief symptom inventory – Anxiety
4. Hospital Anxiety and Depression Scale (HADS) – Anxiety subscale
5. State-Trait Anxiety Questionnaire – State
6. Revised Symptom Checklist (SCL-90-R) – Anxiety dimension

Direction of mental health scale: higher score = greater anxiety.

Comparison 1 – BWMP (diet and/or exercise) versus Control group 1-4

Figure S3a. Anxiety at programme end

	Intervention			Control			Mean Difference
Study or Subgroup	Mean	SD	Total	Mean	SD	Total	IV, Random, 95% CI
**1.7.1 Control group 1**							
-6.7 Fernandez-Ruiz 2018	3	2.55	37	3.5	2.59	37	-0.50 [-1.67, 0.67]

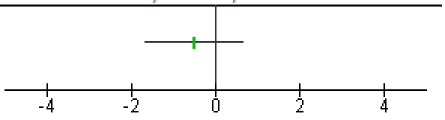

Figure S3b. Anxiety at 1-6 months after programme end

	Intervention			Control				Std. Mean Difference
Study or Subgroup	Mean	SD	Total	Mean	SD	Total	Weight	IV, Random, 95% CI
**1.8.1 Control group 1**								
-1.8 Foster-Schubert 2012	-0.7	1.91	108	0	2.56	80	19.8%	-0.32 [-0.61, -0.02]
**Subtotal (95% CI)**			**108**			**80**	**19.8%**	**-0.32 [-0.61, -0.02]**
Heterogeneity: Not applicable								
Test for overall effect: Z = 2.13 (P = 0.03)								
**1.8.2 Control group 4**								
-6.4 Diabetes Prevention Program R G 2009	-0.89	4.78	998	-0.25	4.8	993	80.2%	-0.13 [-0.22, -0.05]
**Subtotal (95% CI)**			**998**			**993**	**80.2%**	**-0.13 [-0.22, -0.05]**
Heterogeneity: Not applicable								
Test for overall effect: Z = 2.98 (P = 0.003)								
**Total (95% CI)**			**1106**			**1073**	**100.0%**	**-0.17 [-0.31, -0.03]**
Heterogeneity: Tau² = 0.00; Chi² = 1.38, df = 1 (P = 0.24); I² = 27%								
Test for overall effect: Z = 2.34 (P = 0.02)								
Test for subgroup differences: Chi² = 1.38, df = 1 (P = 0.24), I² = 27.4%								

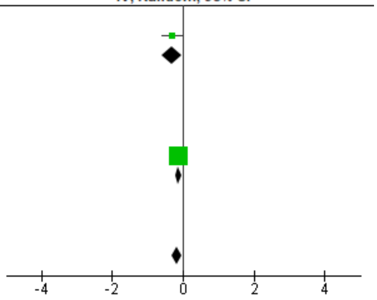

Figure S3c. Anxiety at 7-12 months after programme end

	Intervention			Control			Mean Difference
Study or Subgroup	Mean	SD	Total	Mean	SD	Total	IV, Random, 95% CI
**1.9.1 Control group 1**							
-7.9 Fernandez-Ruiz 2018	5.3	3.53	37	4.7	2.34	37	0.60 [-0.76, 1.96]

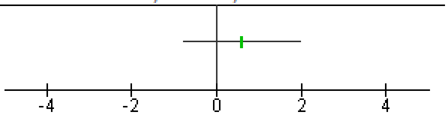

Comparison 2 – BWMP (diet and exercise) versus diet only

Figure S3d. Anxiety at programme end

	Diet and Exercise			Diet only			Mean Difference
Study or Subgroup	Mean	SD	Total	Mean	SD	Total	IV, Random, 95% CI
-4 Snel 2012	-1	3.61	13	-1	0.92	14	0.00 [-2.02, 2.02]

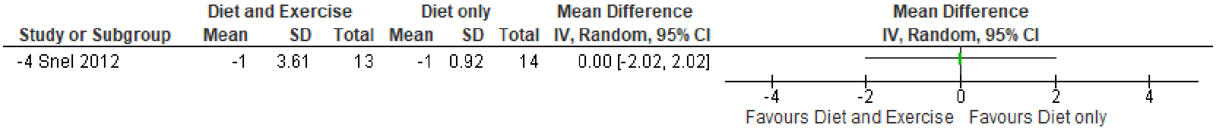

Figure S3e. Anxiety at 1-6 months after programme end

	Diet and Exercise			Diet only			Mean Difference
Study or Subgroup	Mean	SD	Total	Mean	SD	Total	IV, Random, 95% CI
-1.8 Foster-Schubert 2012	-0.7	1.91	108	-1.1	2.05	105	0.40 [-0.13, 0.93]

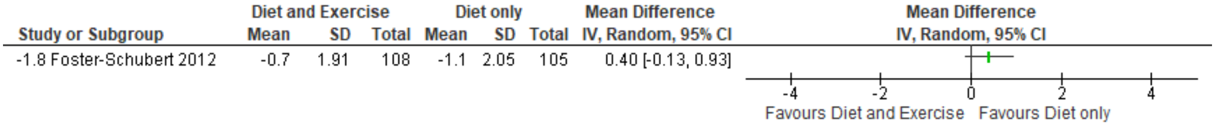

Figure S3f. Anxiety at 13-18 months after programme end

	Diet and Exercise			Diet only			Mean Difference
Study or Subgroup	Mean	SD	Total	Mean	SD	Total	IV, Random, 95% CI
-6 Snel 2012	0	1.77	13	-1	1.4	14	1.00 [-0.21, 2.21]

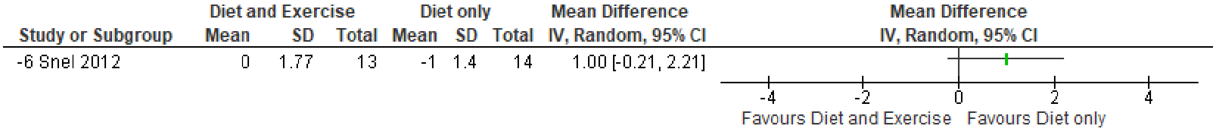

Comparison 3 – BWMP (diet and exercise) versus exercise only

No studies

Comparison 4 – Intervention Vs Comparator intervention

Anxiety head-to-head intervention comparisons

At programme end, one 3-arm study[4] showed no difference when the two most intensive study groups were compared to the least intensive intervention group (Figure S3g). Another study favoured the CBT intervention group versus the behaviours weight loss treatment comparator, despite less weight loss in the CBT group (SMD −1.23 [-1.79, -0.67]; n = 59). Another study[2] favoured the comparator (SMD 0.66 [0.04, 1.28]; n = 42).

At 1-6 months, no difference in anxiety scores remained for the one 3-arm study[4], however the direction of effect favoured the intervention (Figure S3h). This persisted at 7-12 months, where most studies showed a direction of effect that favoured the intervention, however 95% CIs incorporated no difference and favouring of the comparator (Figure S3i). One study[3] favoured the intervention group (SMD -1.35 [-2.01, -0.69] and had CIs excluding no difference; n = 44), which also showed the greatest reduction in weight (weight change difference: -3.6kg). No studies were at overall high risk of bias.

Figure S3g. Anxiety at programme end

Study or Subgroup	Intervention Mean	Intervention SD	Intervention Total	Comparator Mean	Comparator SD	Comparator Total	Std. Mean Difference IV, Random, 95% CI
+0.1 Katzer 2008a (1)	-0.09	0.3	53	-0.09	0.3	81	0.00 [-0.35, 0.35]
+0.4 Cleo 2018 (2)	0.1	0.34	22	-1	2.35	20	0.66 [0.04, 1.28]
+0.8 Katzer 2008b (3)	-0.09	0.4	48	-0.09	0.3	81	0.00 [-0.36, 0.36]
+2.3 Munsch 2007 (4)	-4.07	4.28	32	0.33	2.31	27	-1.23 [-1.79, -0.67]

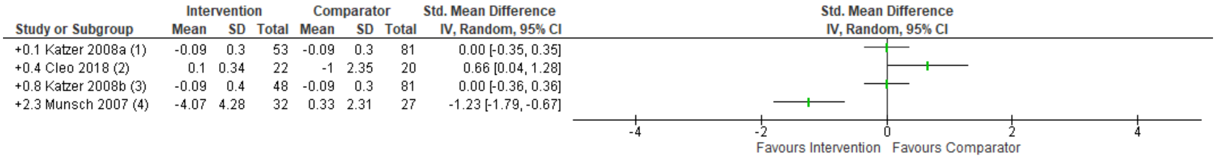

Footnotes
(1) Group 'non-dieting' program based on Relaxation Response Training (P1) Vs Mail-delivered 'non-dieting' program (P3); Same study as Katzer 2008b but different intervention group
(2) DSD Do Something Different Vs The Ten Top Tips
(3) Group 'non-dieting' program (P2) Vs Mail-delivered 'non-dieting' program (P3); Same study as Katzer 2008a but different intervention group
(4) Cognitive-behavioural therapy Vs Behavioural weight loss treatment

Figure S3h. Anxiety at 1-6 months after programme end

Study or Subgroup	Intervention group Mean	Intervention group SD	Intervention group Total	Comparator group Mean	Comparator group SD	Comparator group Total	Mean Difference IV, Random, 95% CI
+1.8 Katzer 2008a (1)	-0.1	0.4	41	-0.07	0.2	68	-0.03 [-0.16, 0.10]
+2.5 Katzer 2008b (2)	-0.18	0.5	39	-0.07	0.2	68	-0.11 [-0.27, 0.05]

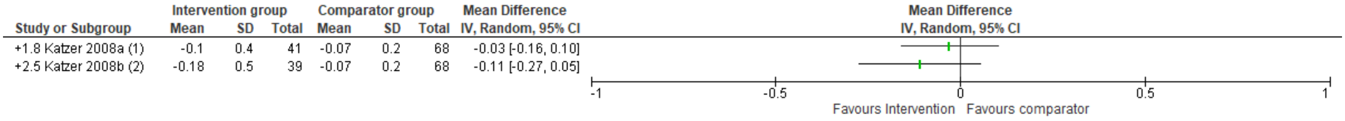

Footnotes
(1) Group 'non-dieting' program based on Relaxation Response Training (P1) Vs Mail-delivered 'non-dieting' program (P3); Same study as Katzer 2008b but different intervention group
(2) Group 'non-dieting' program (P2) Vs Mail-delivered 'non-dieting' program (P3); Same study as Katzer 2008a but different intervention group

Figure S3i. Anxiety at 7-12 months after programme end

Study or Subgroup	Intervention group Mean	Intervention group SD	Intervention group Total	Comparator group Mean	Comparator group SD	Comparator group Total	Std. Mean Difference IV, Random, 95% CI
-3.6 Munsch 2007 (1)	-7.49	6.29	23	0.26	4.86	21	-1.35 [-2.01, -0.69]
-0.6 Katzer 2008a (2)	-0.14	0.3	36	-0.08	0.3	63	-0.20 [-0.61, 0.21]
+1.5 Katzer 2008b (3)	-0.2	0.5	38	-0.08	0.3	63	-0.31 [-0.71, 0.10]
+1.1 Cleo 2018 (4)	-0.7	3.72	22	-0.1	2.86	21	-0.18 [-0.78, 0.42]

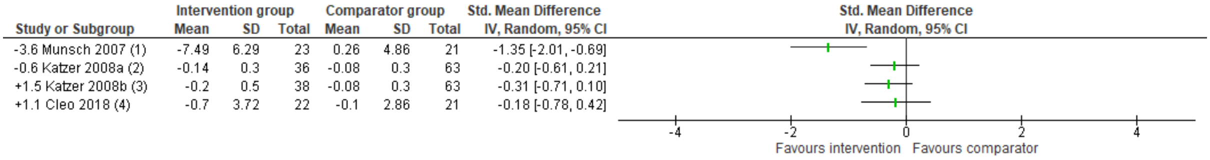

Footnotes
(1) Cognitive-behavioural therapy Vs Behavioural weight loss treatment
(2) Group 'non-dieting' program based on Relaxation Response Training (P1) Vs Mail-delivered 'non-dieting' program (P3); Same study as Katzer 2008b but different intervention group
(3) Group 'non-dieting' program (P2) Vs Mail-delivered 'non-dieting' program (P3); Same study as Katzer 2008a but different intervention group
(4) DSD Do Something Different Vs The Ten Top Tips

Depression and Anxiety

Outcome measures:

1. EQ-5D 5L Anxiety/depression dimension only
2. EQ-5D 3L Anxiety/depression dimension only
3. Depression Anxiety Stress Scale (DASS-21)
4. General Health Questionnaire – 12
5. Hospital Anxiety and Depression Scale (HADS) – Total

Direction of mental health scale: higher score = more severe anxiety or depression

Comparison 1 – BWMP (diet and/or exercise) versus Control group 1-4

Figure S4a. Depression and Anxiety at programme end

	Intervention			Control				Std. Mean Difference
**Study or Subgroup**	**Mean**	**SD**	**Total**	**Mean**	**SD**	**Total**	**Weight**	**IV, Random, 95% CI**
**1.18.1 Control group 1**								
-1.6 van Wier 2011	-0.01	0.33	261	-0.02	0.36	265	42.0%	0.03 [-0.14, 0.20]
-1.0 Bennett 2013	-0.09	0.53	85	-0.11	0.5	92	21.6%	0.04 [-0.26, 0.33]
-0.5 Ash 2006 (1)	-1.27	0.96	35	-0.81	0.81	31	9.4%	-0.51 [-1.00, -0.02]
+0.8 Bennett 2012	-0.109	0.58	110	-0.123	0.459	138	27.1%	0.03 [-0.22, 0.28]
**Subtotal (95% CI)**			**491**			**526**	**100.0%**	**-0.02 [-0.18, 0.14]**
Heterogeneity: Tau² = 0.01; Chi² = 4.34, df = 3 (P = 0.23); I² = 31%								
Test for overall effect: Z = 0.24 (P = 0.81)								
**Total (95% CI)**			**491**			**526**	**100.0%**	**-0.02 [-0.18, 0.14]**
Heterogeneity: Tau² = 0.01; Chi² = 4.34, df = 3 (P = 0.23); I² = 31%								
Test for overall effect: Z = 0.24 (P = 0.81)								
Test for subgroup differences: Not applicable								

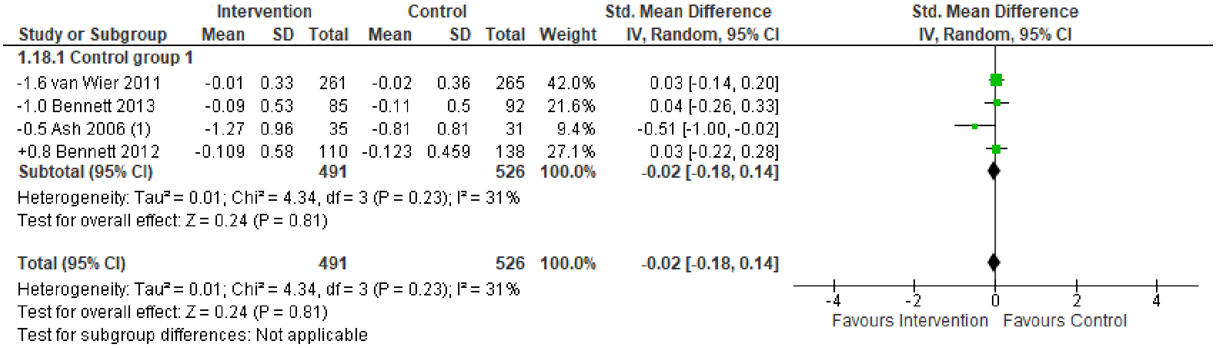

Footnotes
(1) Intervention end at 2 months and follow-up at 3 months

Figure S4b. Depression and Anxiety at 1-6 months after programme end

	Intervention			Control				Std. Mean Difference
**Study or Subgroup**	**Mean**	**SD**	**Total**	**Mean**	**SD**	**Total**	**Weight**	**IV, Random, 95% CI**
**1.17.1 Control Group 1**								
-1.7 Bennett 2013	-0.1	0.49	86	-0.08	0.58	90	29.1%	-0.04 [-0.33, 0.26]
van Wier 2011 (1)	0	0.35	217	-0.03	0.35	211	70.9%	0.09 [-0.10, 0.28]
**Subtotal (95% CI)**			**303**			**301**	**100.0%**	**0.05 [-0.11, 0.21]**
Heterogeneity: Tau² = 0.00; Chi² = 0.47, df = 1 (P = 0.49); I² = 0%								
Test for overall effect: Z = 0.61 (P = 0.54)								
**Total (95% CI)**			**303**			**301**	**100.0%**	**0.05 [-0.11, 0.21]**
Heterogeneity: Tau² = 0.00; Chi² = 0.47, df = 1 (P = 0.49); I² = 0%								
Test for overall effect: Z = 0.61 (P = 0.54)								
Test for subgroup differences: Not applicable								

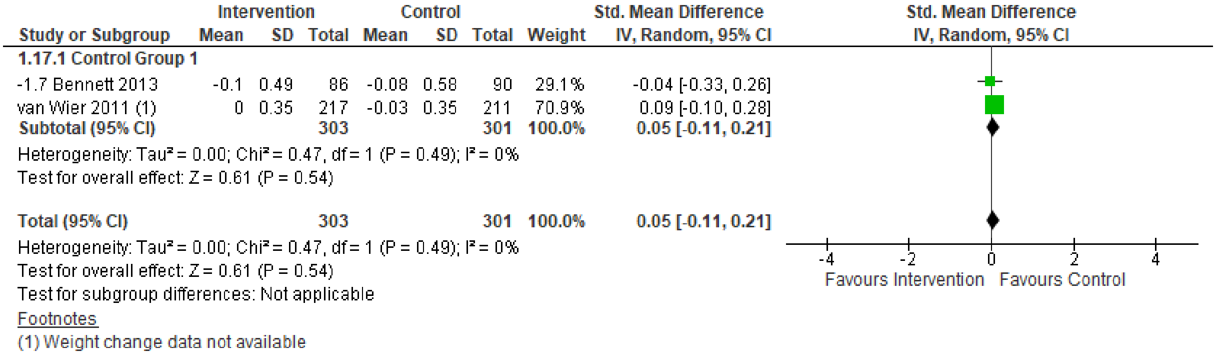

Footnotes
(1) Weight change data not available

Figure S4c. Depression and Anxiety at 7-12 months after programme end

Study or Subgroup	Intervention Mean	Intervention SD	Intervention Total	Control Mean	Control SD	Control Total	Weight	Std. Mean Difference IV, Random, 95% CI
**1.19.1 Control Group 1**								
van Weir 2011 (1)	0.03	0.37	183	-0.03	0.35	173	31.4%	0.17 [-0.04, 0.37]
-3.9 Foley 2016	-0.077	0.477	25	-0.038	0.505	157	22.6%	-0.08 [-0.50, 0.34]
-3.4 Ash 2006	-2.11	2.24	22	-0.14	1.29	21	15.3%	-1.05 [-1.69, -0.41]
-1.0 Bennett 2012	-0.077	0.547	142	-0.098	0.567	164	30.7%	0.04 [-0.19, 0.26]
**Subtotal (95% CI)**			**372**			**515**	**100.0%**	**-0.11 [-0.45, 0.22]**
Heterogeneity: Tau² = 0.08; Chi² = 12.80, df = 3 (P = 0.005); I² = 77%								
Test for overall effect: Z = 0.68 (P = 0.50)								
**Total (95% CI)**			**372**			**515**	**100.0%**	**-0.11 [-0.45, 0.22]**
Heterogeneity: Tau² = 0.08; Chi² = 12.80, df = 3 (P = 0.005); I² = 77%								
Test for overall effect: Z = 0.68 (P = 0.50)								
Test for subgroup differences: Not applicable								

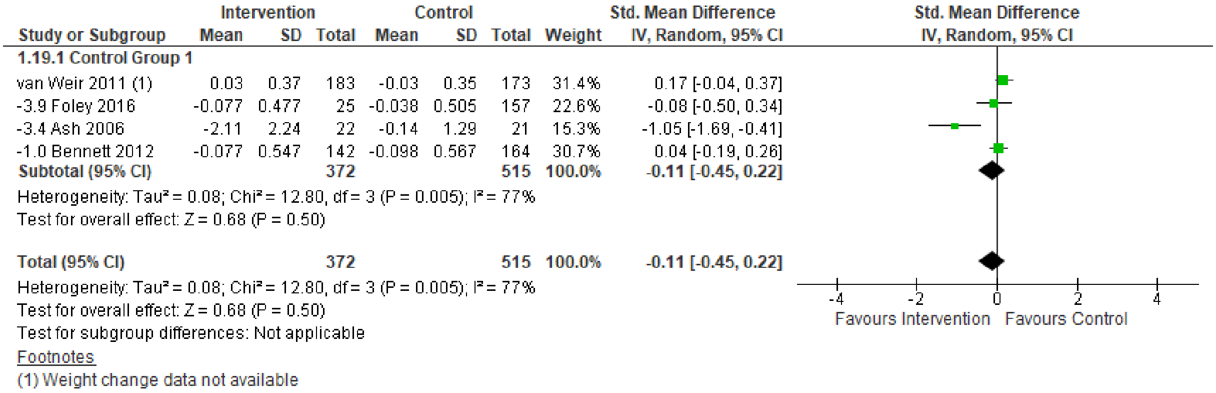

Footnotes
(1) Weight change data not available

Figure S4d. Depression and Anxiety at 13-18months after programme end

Study or Subgroup	Intervention Mean	Intervention SD	Intervention Total	Control Mean	Control SD	Control Total	Mean Difference IV, Random, 95% CI
**1.16.1 Control group 1**							
-0.4 van Weir 2011	-0.03	0.33	260	-0.07	0.3	258	0.04 [-0.01, 0.09]

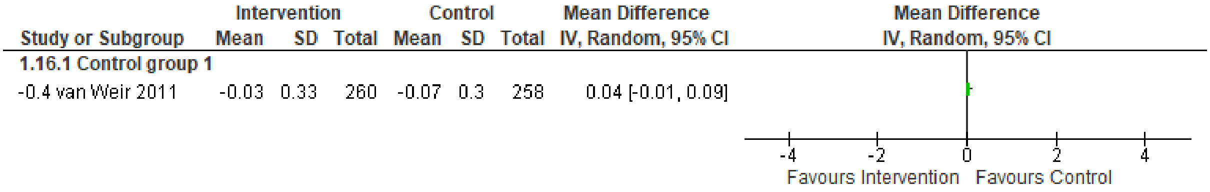

Comparison 2 – BWMP (diet and exercise) versus diet only

Figure S4e. Depression and Anxiety at programme end

Study or Subgroup	Diet and Exercise Mean	Diet and Exercise SD	Diet and Exercise Total	Diet only Mean	Diet only SD	Diet only Total	Mean Difference IV, Random, 95% CI
-4 Snel 2012	-3	1.02	13	-3	0.92	14	0.00 [-0.73, 0.73]

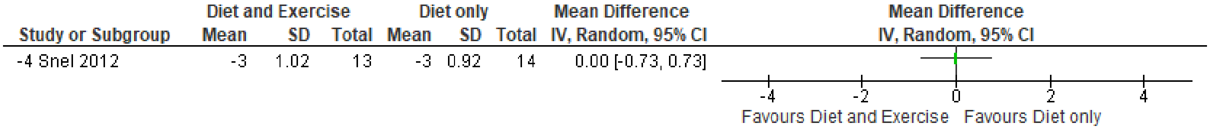

Figure S4f. Depression and Anxiety at 13-18 months after programme end

Study or Subgroup	Diet and Exercise Mean	Diet and Exercise SD	Diet and Exercise Total	Diet only Mean	Diet only SD	Diet only Total	Mean Difference IV, Random, 95% CI
-6 Snel 2012	-1	1.77	13	-1	1.4	14	0.00 [-1.21, 1.21]

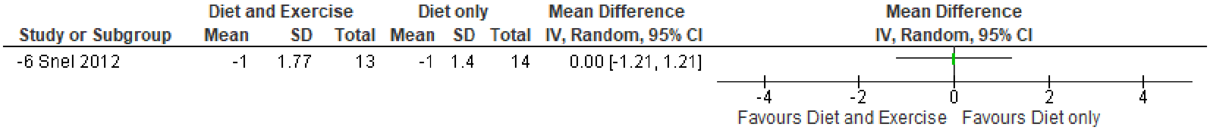

Comparison 3 – BWMP (diet and exercise) versus exercise only

No studies

Comparison 4 – Intervention Vs Comparator intervention

Depression and Anxiety head-to-head intervention comparisons

Most studies at and up to 18 months after programme end found no difference in depression and anxiety scores between active intervention arms (Figure S4g-j), however two of these studies were at

high risk of bias.[7, 8] One study[7] favoured the most intensive intervention arm at programme end (MD -0.82 [-1.26, -0.37]; n=86), however this study was at high risk of bias.

Figure S4g. Depression and Anxiety at programme end

Study or Subgroup	Intervention Mean	Intervention SD	Intervention Total	Comparator Mean	Comparator SD	Comparator Total	Std. Mean Difference IV, Random, 95% CI
-4.1 Mensinger 2016 (1)	-1.9	18.73	33	-0.8	5.5	37	-0.08 [-0.55, 0.39]
-0.9 van Wier 2011 (2)	-0.01	0.33	261	0	0.34	258	-0.03 [-0.20, 0.14]
+0.7 Ash 2006 (3)	-1.27	0.96	35	0.09	1.99	51	-0.82 [-1.26, -0.37]

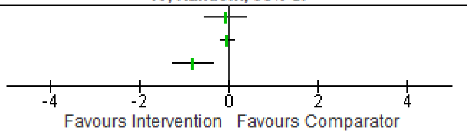

Footnotes
(1) Weight Loss Program Vs Weight-Neutral Program
(2) Phone Group Vs Internet Group
(3) Fat Booters Incorporated Vs Individualised Dietetic Treatment; End of treatment at 2 months and follow-up at 3 months

Figure S4h. Depression and Anxiety at 1-6 months after programme end

Study or Subgroup	Intervention Mean	Intervention SD	Intervention Total	Comparator Mean	Comparator SD	Comparator Total	Mean Difference IV, Random, 95% CI
van Weir 2011 (1)	0	0.35	217	0.01	0.33	183	-0.01 [-0.08, 0.06]

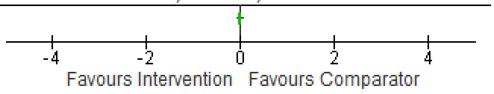

Footnotes
(1) Phone Group Vs Internet Group; No weight change data available

Figure S4i. Depression and Anxiety at 7-12 months after programme end

Study or Subgroup	Intervention Mean	Intervention SD	Intervention Total	Comparator Mean	Comparator SD	Comparator Total	Std. Mean Difference IV, Random, 95% CI
-1.1 Ash 2006 (1)	-2.11	2.24	25	-1.38	1.43	44	-0.41 [-0.91, 0.09]
van Weir 2011 (2)	0.03	0.37	183	-0.03	0.28	155	0.18 [-0.03, 0.39]

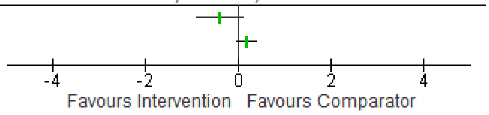

Footnotes
(1) Fat Booters Incorporated Vs Individualised Dietetic Treatment
(2) Phone Group Vs Internet Group; No weight change data available

Figure S4j. Depression and Anxiety at 13-18 months after programme end

Study or Subgroup	Intervention Mean	Intervention SD	Intervention Total	Comparator Mean	Comparator SD	Comparator Total	Std. Mean Difference IV, Random, 95% CI
-2.9 Mensinger 2016 (1)	4.9	18.47	21	3.2	8.02	19	0.11 [-0.51, 0.74]
+0.4 van Wier 2011 (2)	-0.03	0.33	260	-0.06	0.24	253	0.10 [-0.07, 0.28]

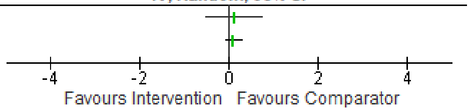

Footnotes
(1) Weight Loss Program Vs Weight-Neutral Program
(2) Phone Group Vs Internet Group

Self-Esteem

Outcome measures:

1. Rosenberg Self-Esteem Scale (RSES)

Direction of mental health scale: higher score = higher self-esteem.

Comparison 1 – BWMP (diet and/or exercise) versus Control group 1-4

*Figure S5a. Self-esteem at programme end**

	Intervention			Control				Std. Mean Difference
**Study or Subgroup**	**Mean**	**SD**	**Total**	**Mean**	**SD**	**Total**	**Weight**	**IV, Random, 95% CI**
**1.10.1 Control group 1**								
-5.4 Hunt 2014	0.3	0.37	330	0.1	0.33	344	68.5%	0.57 [0.42, 0.72]
**Subtotal (95% CI)**			**330**			**344**	**68.5%**	**0.57 [0.42, 0.72]**
Heterogeneity: Not applicable								
Test for overall effect: Z = 7.26 (P < 0.00001)								
**1.10.2 Control group 3**								
+0.8 deZwaan 2017	2.6	1.98	85	1.9	2.07	77	31.5%	0.34 [0.03, 0.66]
**Subtotal (95% CI)**			**85**			**77**	**31.5%**	**0.34 [0.03, 0.66]**
Heterogeneity: Not applicable								
Test for overall effect: Z = 2.17 (P = 0.03)								
**Total (95% CI)**			**415**			**421**	**100.0%**	**0.50 [0.29, 0.71]**
Heterogeneity: Tau² = 0.01; Chi² = 1.63, df = 1 (P = 0.20); I² = 39%								
Test for overall effect: Z = 4.75 (P < 0.00001)								
Test for subgroup differences: Chi² = 1.63, df = 1 (P = 0.20), I² = 38.8%								

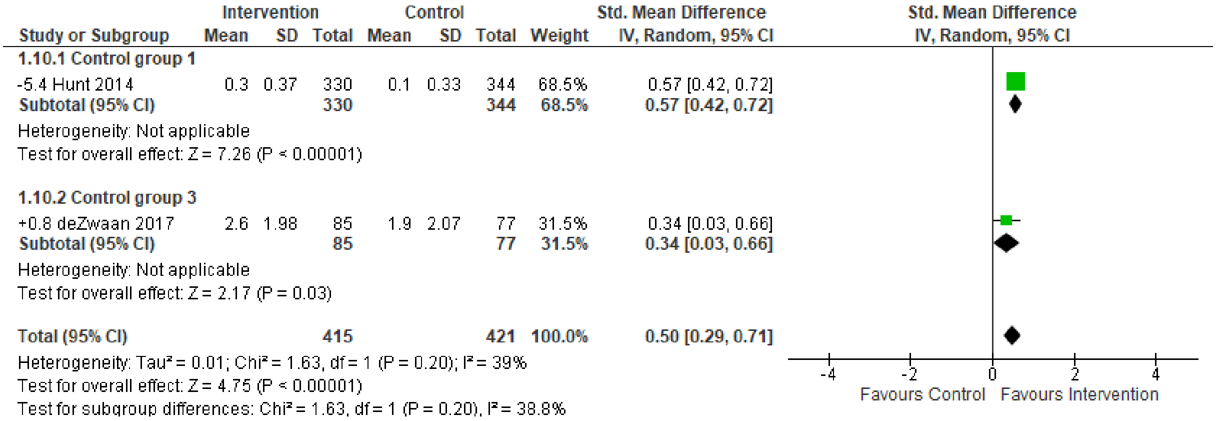

Figure S5b. Self-esteem at 1-6 months after programme end

	Intervention			Control			Mean Difference
**Study or Subgroup**	**Mean**	**SD**	**Total**	**Mean**	**SD**	**Total**	**IV, Random, 95% CI**
**1.11.1 Control group 3**							
-0.5 deZwaan 2017	3.2	3.37	80	2.9	2.91	70	0.30 [-0.71, 1.31]

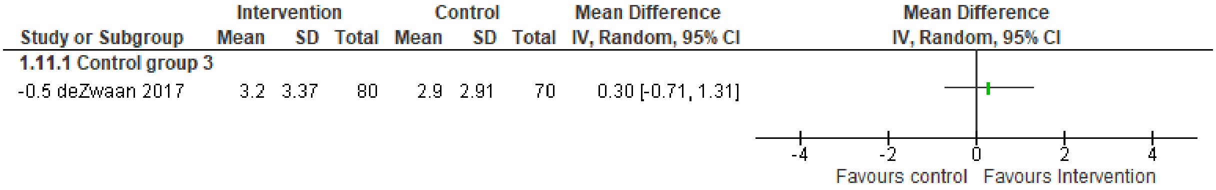

Figure S5c. Self-esteem at 7-12 months after programme end

	Intervention			Control			Mean Difference
**Study or Subgroup**	**Mean**	**SD**	**Total**	**Mean**	**SD**	**Total**	**IV, Random, 95% CI**
**1.12.1 Control group 1**							
-5.0 Hunt 2014	0.3	0.36	213	0.1	0.33	250	0.20 [0.14, 0.26]

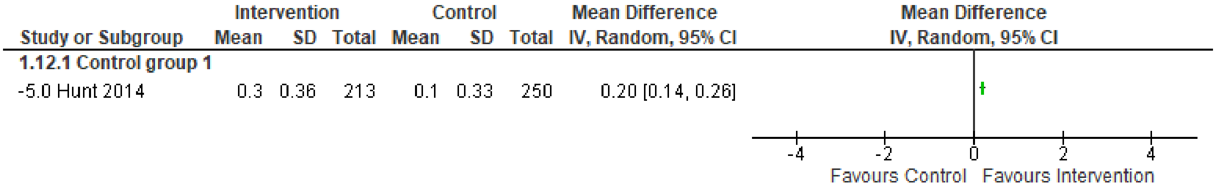

Comparison 2 – BWMP (diet and exercise) versus diet only

No studies

Comparison 3 – BWMP (diet and exercise) versus exercise only

No studies

Comparison 4 – Intervention Vs Comparator intervention

Self-esteem head-to-head intervention comparisons

All studies at and up to 18 months after programme end showed no difference in changes in self-esteem scores between two intervention groups, with 95% CIs including the possibility of no difference as well as favouring both arms. (Figures S5d-g). At programme end, the direction of effect mostly favoured the intervention, however by 1-6 months after programme end, the direction of effect favoured the comparator, with CIs of one study not favouring the intervention arm (Figure S5e). Two studies reporting at programme end, 1-6 months and 13-18 months were at high risk of bias.[1, 8]

Figure S5d. Self-esteem at programme end

Study or Subgroup	Intervention Mean	Intervention SD	Intervention Total	Comparator Mean	Comparator SD	Comparator Total	Std. Mean Difference IV, Random, 95% CI
-5.1 Bacon 2002 (1)	2.1	2.95	23	1.5	1.81	36	0.26 [-0.27, 0.78]
-4.1 Mensinger 2016 (2)	1.6	4.02	33	2.3	3.4	37	-0.19 [-0.66, 0.28]
+0.7 Ames 2005 (3)	5.7	2.8	13	5.5	3.11	13	0.07 [-0.70, 0.83]
+0.5 Ramirez 2001 (4)	4.2	1.78	38	3.8	2.31	27	0.20 [-0.30, 0.69]

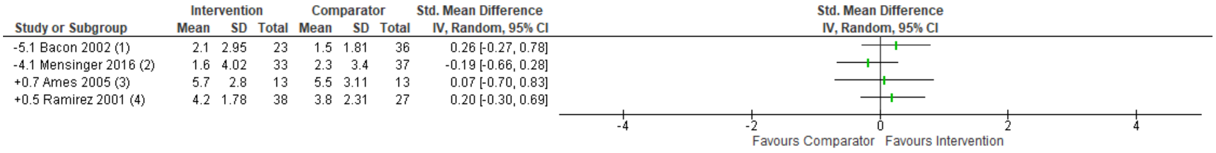

Footnotes
(1) Diet program Vs Non-diet wellness program
(2) Weight Loss Program Vs Weight-Neutral Program
(3) Reformulated cognitive-behavioural treatment Vs Standard behavioural treatment; Mean values multiplied by -1 to ensure scales point in the same direction (higher scores indicate higher self-esteem)
(4) Weight control plus body image therapy Vs Weight control

Figure S5e. Self-esteem at 1-6 months after programme end

Study or Subgroup	Intervention Mean	Intervention SD	Intervention Total	Comparator Mean	Comparator SD	Comparator Total	Std. Mean Difference IV, Random, 95% CI
-0.2 Ames 2005 (1)	3.6	3.63	13	6.4	3.11	13	-0.80 [-1.61, 0.00]
-0.2 Ramirez 2001 (2)	3.9	2.76	38	4.4	2.97	27	-0.17 [-0.67, 0.32]

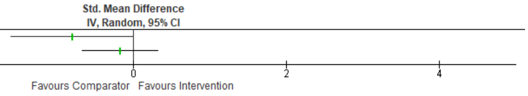

Footnotes
(1) Reformulated cognitive-behavioural treatment Vs Standard behavioural treatment; Mean values multiplied by -1 to ensure scales point in the same direction (higher scores indicate higher self-esteem)
(2) Weight control plus body image therapy Vs Weight control

Figure S5f. Self-esteem at 7 - 12 months after programme end

Study or Subgroup	Intervention Mean	Intervention SD	Intervention Total	Comparator Mean	Comparator SD	Comparator Total	Mean Difference IV, Random, 95% CI
-6.2 Bacon 2002 (1)	2	2.4	29	1.5	3.28	23	0.50 [-1.10, 2.10]
-2.3 Ramirez 2001 (2)	2.7	3.19	38	2.7	2.39	27	0.00 [-1.36, 1.36]

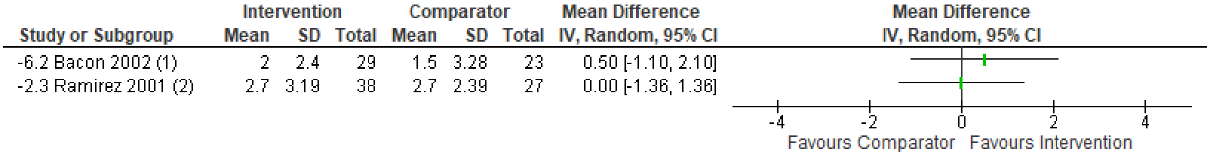

Footnotes
(1) Diet program Vs Non-diet wellness program
(2) Reformulated cognitive-behavioural treatment Vs Standard behavioural treatment

Figure S5g. Self-esteem at 13 - 18 months after programme end

	Intervention			Comparator			Mean Difference
Study or Subgroup	Mean	SD	Total	Mean	SD	Total	IV, Random, 95% CI
-2.9 Mensinger 2016 (1)	1.5	3.59	21	1.9	2.89	19	-0.40 [-2.41, 1.61]

Mean Difference
IV, Random, 95% CI

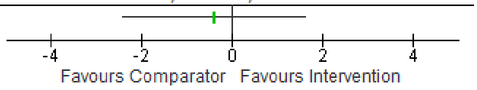

Footnotes
(1) Weight Loss Program Vs Weight-Neutral Program

Mental health composite scales

Outcome measures:

1. RAND 36-Item Health Survey - emotional wellbeing
2. Rand 36-item Health Survey mental health composite score
3. Short Form-12 (MCS)
4. Short Form-36v2 Mental Health Component

Direction of mental health scale: higher score = better mental health

Comparison 1 – BWMP (diet and/or exercise) versus Control group 1-4

Figure S6a. Component Summaries at programme end

Study or Subgroup	Intervention Mean	Intervention SD	Intervention Total	Control Mean	Control SD	Control Total	Weight	Std. Mean Difference IV, Random, 95% CI
**1.17.1 Control group 1**								
-6.7 Fernandez-Ruiz 2018	10.9	6.41	37	-5.5	4.78	37	6.4%	2.87 [2.21, 3.53]
-5.4 Hunt 2014	3.2	8.24	328	1.5	8.17	345	10.4%	0.21 [0.06, 0.36]
-3.7 Huseinovic 2016	3.7	10.2	47	-1.3	9	53	8.6%	0.52 [0.12, 0.92]
-1.6 van Wier 2011 (1)	0.04	12.04	257	1.52	10.23	258	10.3%	-0.13 [-0.31, 0.04]
-0.6 Conroy 2015	-1.74	10.95	41	-1.5	7.77	27	7.8%	-0.02 [-0.51, 0.46]
**Subtotal (95% CI)**			**710**			**720**	**43.6%**	**0.60 [0.05, 1.16]**
Heterogeneity: Tau² = 0.36; Chi² = 80.07, df = 4 (P < 0.00001); I² = 95%								
Test for overall effect: Z = 2.14 (P = 0.03)								
**1.17.2 Control group 3**								
-7.9 The Look AHEAD Research Group 2010	0.06	2.5	2429	-0.6	2.22	2386	10.8%	0.28 [0.22, 0.34]
-3.8 Schubel 2016 (2)	-3.2	3.8	44	8.1	20.3	47	8.4%	-0.76 [-1.18, -0.33]
+0.2 Tapsell 2017	2.77	4.38	96	3.27	2.28	51	9.1%	-0.13 [-0.47, 0.21]
**Subtotal (95% CI)**			**2569**			**2484**	**28.3%**	**-0.17 [-0.75, 0.41]**
Heterogeneity: Tau² = 0.24; Chi² = 27.21, df = 2 (P < 0.00001); I² = 93%								
Test for overall effect: Z = 0.58 (P = 0.56)								
**1.17.3 Control group 4**								
-4.7 Ard 2004 (3)	0.9	9.22	236	0.1	9.18	234	10.3%	0.09 [-0.09, 0.27]
-3.7 Goodwin 2014	1.7	9.7	151	1.6	9.9	144	10.0%	0.01 [-0.22, 0.24]
-3.4 Ng 2015 (4)	5.1	3.08	61	-1.5	3.54	43	7.9%	2.00 [1.52, 2.48]
**Subtotal (95% CI)**			**448**			**421**	**28.2%**	**0.66 [-0.16, 1.48]**
Heterogeneity: Tau² = 0.49; Chi² = 58.41, df = 2 (P < 0.00001); I² = 97%								
Test for overall effect: Z = 1.58 (P = 0.11)								
**Total (95% CI)**			**3727**			**3625**	**100.0%**	**0.36 [0.10, 0.62]**
Heterogeneity: Tau² = 0.16; Chi² = 167.97, df = 10 (P < 0.00001); I² = 94%								
Test for overall effect: Z = 2.69 (P = 0.007)								
Test for subgroup differences: Chi² = 4.42, df = 2 (P = 0.11), I² = 54.8%								

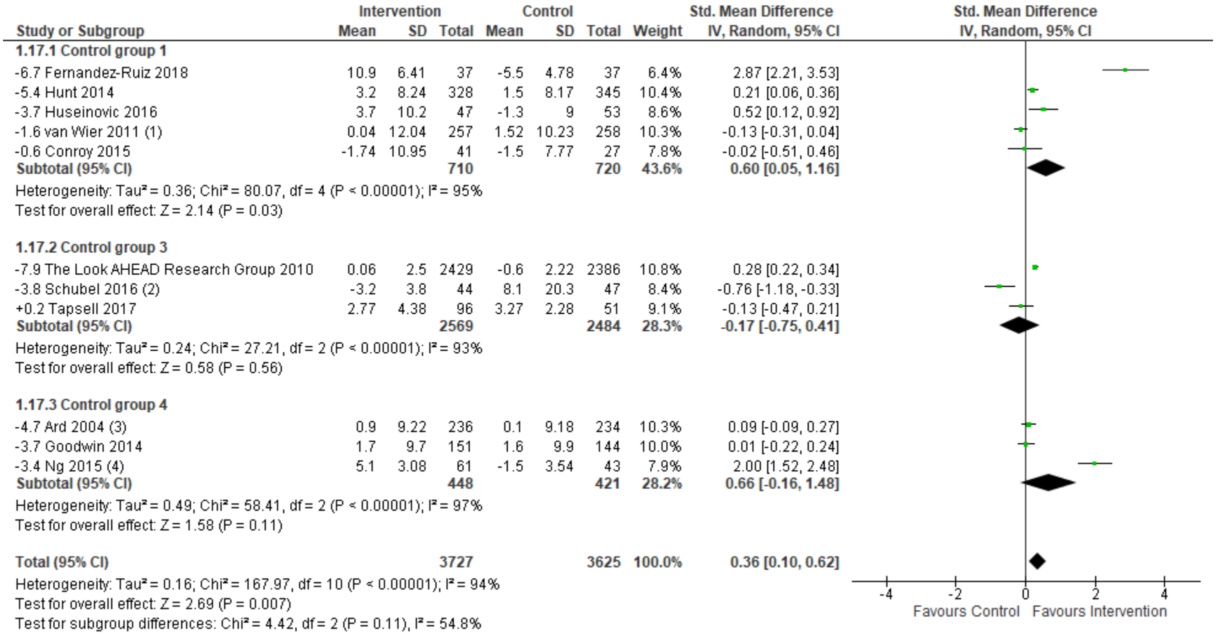

Footnotes
(1) Phone Group Vs Control - Brochure
(2) Intermittent calorie restriction Vs Control group - healthy diet
(3) Established plus Dietary Approaches to Stop Hypertension (DASH) Vs Advice only
(4) Number of participants (N) imputed from N at baseline

Figure S6a1. Assessment for non-reporting bias: Funnel plot

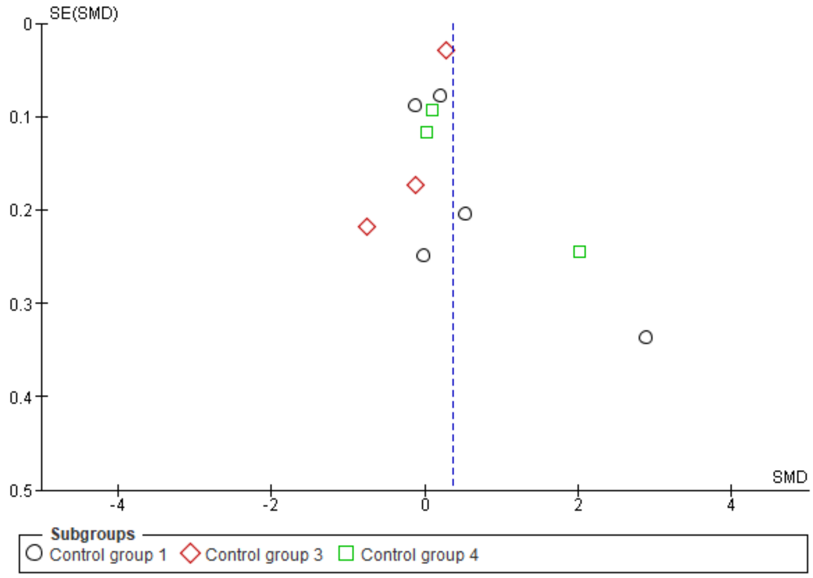

Figure S6b. Component Summaries at 1-6 months after programme end

Considerable heterogeneity unexplained by weight change differences, control subgroups or by the removal of studies at high risk of bias.

Study or Subgroup	Intervention Mean	Intervention SD	Intervention Total	Control Mean	Control SD	Control Total	Weight	Std. Mean Difference IV, Random, 95% CI
**1.15.1 Control group 1**								
van Weir 2011 (1)	1.1	13.6	216	0.71	13.56	207	25.7%	0.03 [-0.16, 0.22]
-8.2 Foster-Schubert 2012 (2)	3.2	3.53	108	0.2	3.57	80	22.2%	0.84 [0.54, 1.14]
**Subtotal (95% CI)**			**324**			**287**	**47.8%**	**0.43 [-0.37, 1.22]**
Heterogeneity: Tau² = 0.31; Chi² = 19.97, df = 1 (P < 0.00001); I² = 95%								
Test for overall effect: Z = 1.05 (P = 0.29)								
**1.15.2 Control group 4**								
-6.4 Diabetes Prevention Program R G 2009	-0.7	8.67	1017	-1.16	8.33	1018	28.0%	0.05 [-0.03, 0.14]
-3.9 Goodwin 2014	0.7	8.7	131	2.4	8.7	132	24.1%	-0.19 [-0.44, 0.05]
**Subtotal (95% CI)**			**1148**			**1150**	**52.2%**	**-0.04 [-0.28, 0.19]**
Heterogeneity: Tau² = 0.02; Chi² = 3.59, df = 1 (P = 0.06); I² = 72%								
Test for overall effect: Z = 0.36 (P = 0.72)								
**Total (95% CI)**			**1472**			**1437**	**100.0%**	**0.16 [-0.13, 0.46]**
Heterogeneity: Tau² = 0.08; Chi² = 30.07, df = 3 (P < 0.00001); I² = 90%								
Test for overall effect: Z = 1.07 (P = 0.28)								
Test for subgroup differences: Chi² = 1.23, df = 1 (P = 0.27), I² = 18.6%								

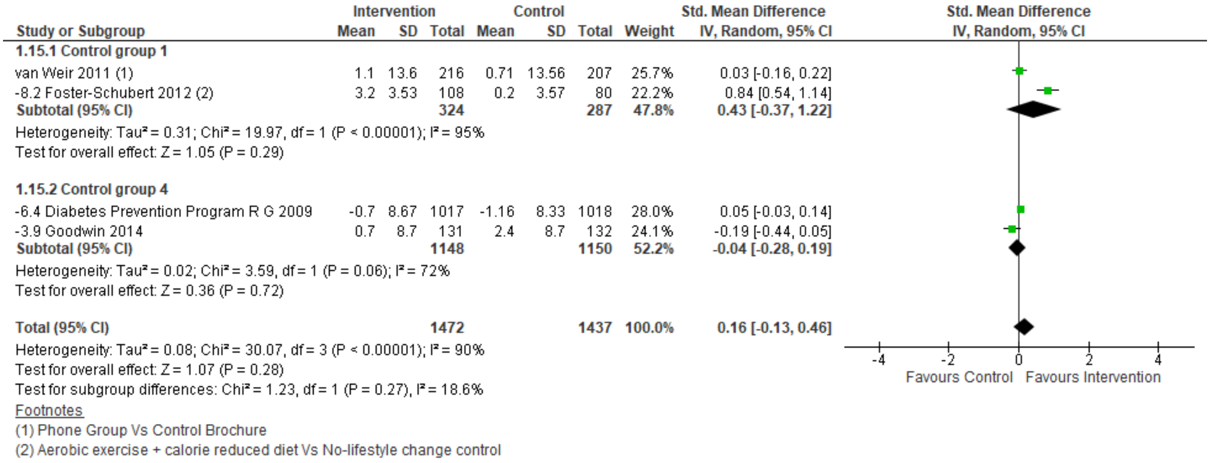

Footnotes
(1) Phone Group Vs Control Brochure
(2) Aerobic exercise + calorie reduced diet Vs No-lifestyle change control

Figure S6c. Component Summaries at 7-12 months after programme end

Considerable heterogeneity unexplained by weight change differences, control subgroups or by the removal of studies at high risk of bias.

Study or Subgroup	Intervention Mean	Intervention SD	Intervention Total	Control Mean	Control SD	Control Total	Weight	Std. Mean Difference IV, Random, 95% CI
**1.18.1 Control group 1**								
van Weir 2011 (1)	0.07	13.66	183	1.73	11.76	168	10.6%	-0.13 [-0.34, 0.08]
-7.9 Fernandez-Ruiz 2018	15.5	6.6	37	-8.5	5.04	37	4.7%	4.04 [3.23, 4.85]
-5.0 Hunt 2014	1.9	8.54	316	1.6	7.62	351	11.1%	0.04 [-0.11, 0.19]
-3.7 Huseinovic 2016	3.3	11	45	-1.7	11.8	48	8.4%	0.43 [0.02, 0.85]
-3.5 Schubel 2016 (2)	3.4	28.6	43	6.4	18.6	46	8.4%	-0.12 [-0.54, 0.29]
0.0 Conroy 2015 (3)	1.04	10.12	37	1.21	8.18	24	7.3%	-0.02 [-0.53, 0.50]
**Subtotal (95% CI)**			**661**			**674**	**50.6%**	**0.58 [0.01, 1.16]**
Heterogeneity: Tau² = 0.47; Chi² = 100.07, df = 5 (P < 0.00001); I² = 95%								
Test for overall effect: Z = 1.98 (P = 0.05)								
**1.18.2 Control group 3**								
-5.4 The Look AHEAD Research Group 2010	-0.7	4.37	2372	-0.47	3.44	2326	11.6%	-0.06 [-0.12, -0.00]
-1.4 Tapsell 2017	4.87	6.55	44	2.3	4.79	60	8.6%	0.46 [0.06, 0.85]
**Subtotal (95% CI)**			**2416**			**2386**	**20.2%**	**0.16 [-0.34, 0.66]**
Heterogeneity: Tau² = 0.11; Chi² = 6.40, df = 1 (P = 0.01); I² = 84%								
Test for overall effect: Z = 0.63 (P = 0.53)								
**1.18.3 Control group 4**								
-3.0 Goodwin 2014	0.7	10.2	117	2.2	9.1	126	10.2%	-0.16 [-0.41, 0.10]
-2.8 Ng 2015	2.3	5.5	44	1.2	4.15	37	8.1%	0.22 [-0.22, 0.66]
-2.8 Ard 2004 (4)	0.2	9.37	244	0.1	9.41	246	10.9%	0.01 [-0.17, 0.19]
**Subtotal (95% CI)**			**405**			**409**	**29.2%**	**-0.02 [-0.18, 0.14]**
Heterogeneity: Tau² = 0.00; Chi² = 2.38, df = 2 (P = 0.30); I² = 16%								
Test for overall effect: Z = 0.22 (P = 0.83)								
**Total (95% CI)**			**3482**			**3469**	**100.0%**	**0.24 [0.01, 0.47]**
Heterogeneity: Tau² = 0.12; Chi² = 112.93, df = 10 (P < 0.00001); I² = 91%								
Test for overall effect: Z = 2.08 (P = 0.04)								
Test for subgroup differences: Chi² = 4.13, df = 2 (P = 0.13), I² = 51.6%								

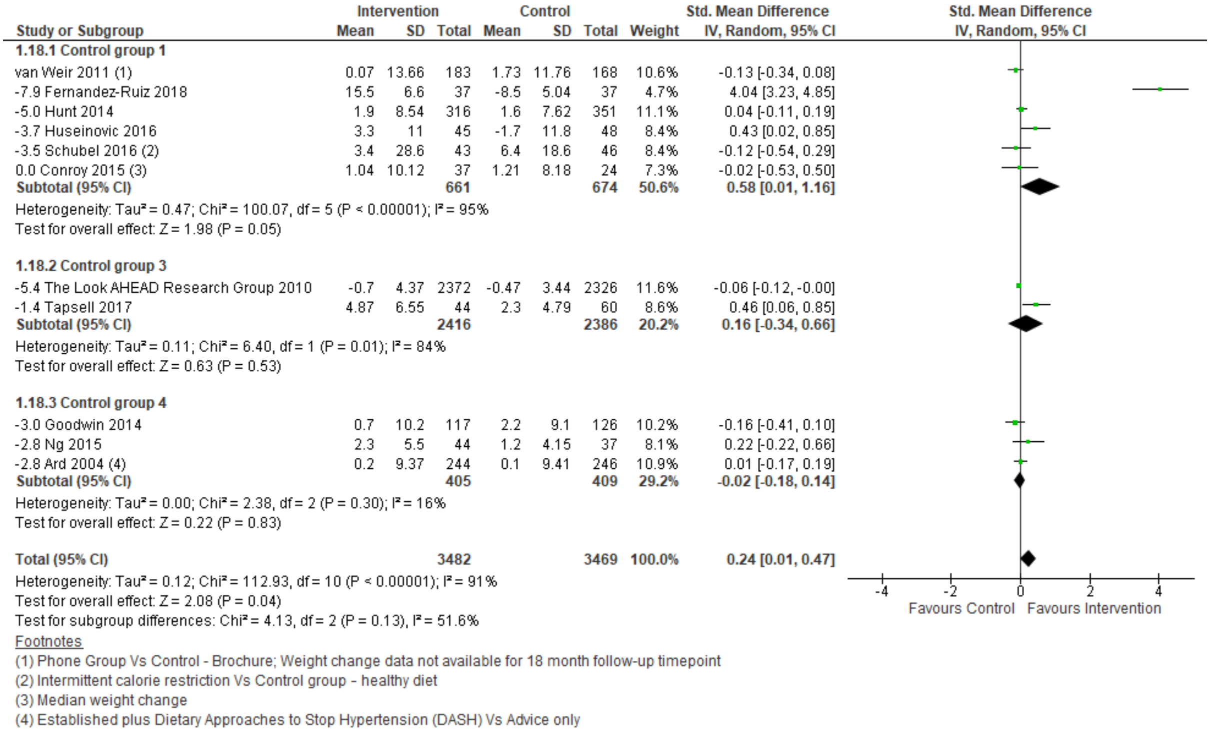

Footnotes
(1) Phone Group Vs Control - Brochure; Weight change data not available for 18 month follow-up timepoint
(2) Intermittent calorie restriction Vs Control group - healthy diet
(3) Median weight change
(4) Established plus Dietary Approaches to Stop Hypertension (DASH) Vs Advice only

Figure S6d. Component Summaries at 13-18 months after programme end

Study or Subgroup	Intervention Mean	Intervention SD	Intervention Total	Control Mean	Control SD	Control Total	Weight	Std. Mean Difference IV, Random, 95% CI
**1.15.1 Control group 1**								
-0.4 van Weir 2011 (1)	0.55	13.83	199	2.6	11.93	173	58.0%	-0.16 [-0.36, 0.05]
**Subtotal (95% CI)**			**199**			**173**	**58.0%**	**-0.16 [-0.36, 0.05]**
Heterogeneity: Not applicable								
Test for overall effect: Z = 1.51 (P = 0.13)								
**1.15.2 Control group 4**								
-2.8 Goodwin 2014	1.4	10.1	120	1.1	10.7	124	42.0%	0.03 [-0.22, 0.28]
**Subtotal (95% CI)**			**120**			**124**	**42.0%**	**0.03 [-0.22, 0.28]**
Heterogeneity: Not applicable								
Test for overall effect: Z = 0.22 (P = 0.82)								
**Total (95% CI)**			**319**			**297**	**100.0%**	**-0.08 [-0.26, 0.10]**
Heterogeneity: Tau² = 0.00; Chi² = 1.27, df = 1 (P = 0.26); I² = 22%								
Test for overall effect: Z = 0.86 (P = 0.39)								
Test for subgroup differences: Chi² = 1.27, df = 1 (P = 0.26), I² = 21.5%								

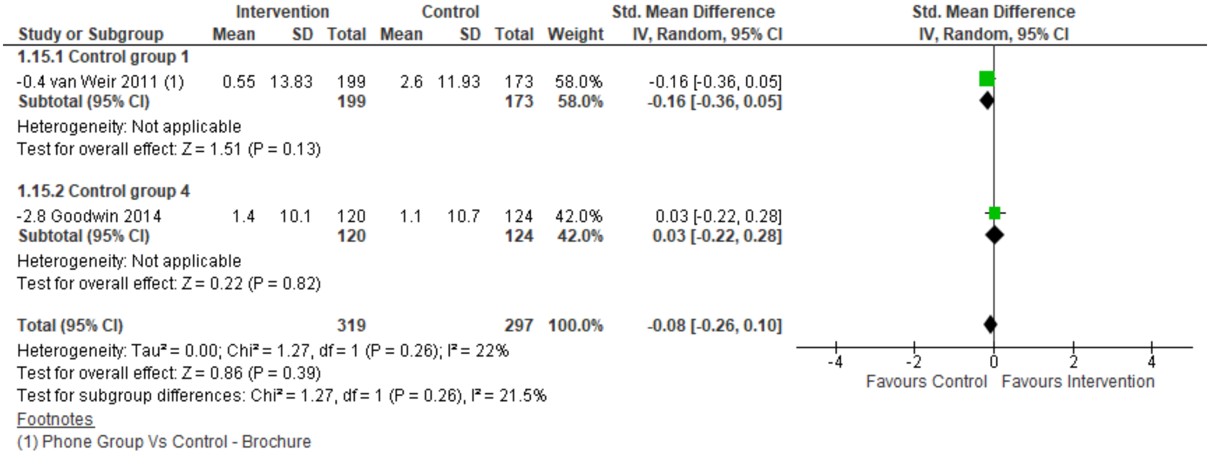

Footnotes
(1) Phone Group Vs Control - Brochure

Figure S6e. Component Summaries at 19-24 months after programme end

Study or Subgroup	Intervention Mean	Intervention SD	Intervention Total	Control Mean	Control SD	Control Total	Weight	Std. Mean Difference IV, Random, 95% CI
**1.15.1 Control group 1**								
-2.3 Huseinovic 2016	-1.8	13.6	42	-5.3	12.9	47	32.0%	0.26 [-0.16, 0.68]
**Subtotal (95% CI)**			**42**			**47**	**32.0%**	**0.26 [-0.16, 0.68]**
Heterogeneity: Not applicable								
Test for overall effect: Z = 1.23 (P = 0.22)								
**1.15.2 Control group 2**								
-4.3 Appel 2011	-0.5	0.76	100	0.62	0.95	88	33.2%	-1.31 [-1.62, -0.99]
**Subtotal (95% CI)**			**100**			**88**	**33.2%**	**-1.31 [-1.62, -0.99]**
Heterogeneity: Not applicable								
Test for overall effect: Z = 8.10 (P < 0.00001)								
**1.15.3 Control group 3**								
-4.2 The Look AHEAD Research Group 2010	-0.7	4.46	2343	-0.54	3.57	2326	34.8%	-0.04 [-0.10, 0.02]
**Subtotal (95% CI)**			**2343**			**2326**	**34.8%**	**-0.04 [-0.10, 0.02]**
Heterogeneity: Not applicable								
Test for overall effect: Z = 1.35 (P = 0.18)								
**Total (95% CI)**			**2485**			**2461**	**100.0%**	**-0.36 [-1.20, 0.47]**
Heterogeneity: Tau² = 0.52; Chi² = 62.25, df = 2 (P < 0.00001); I² = 97%								
Test for overall effect: Z = 0.85 (P = 0.39)								
Test for subgroup differences: Chi² = 62.25, df = 2 (P < 0.00001), I² = 96.8%								

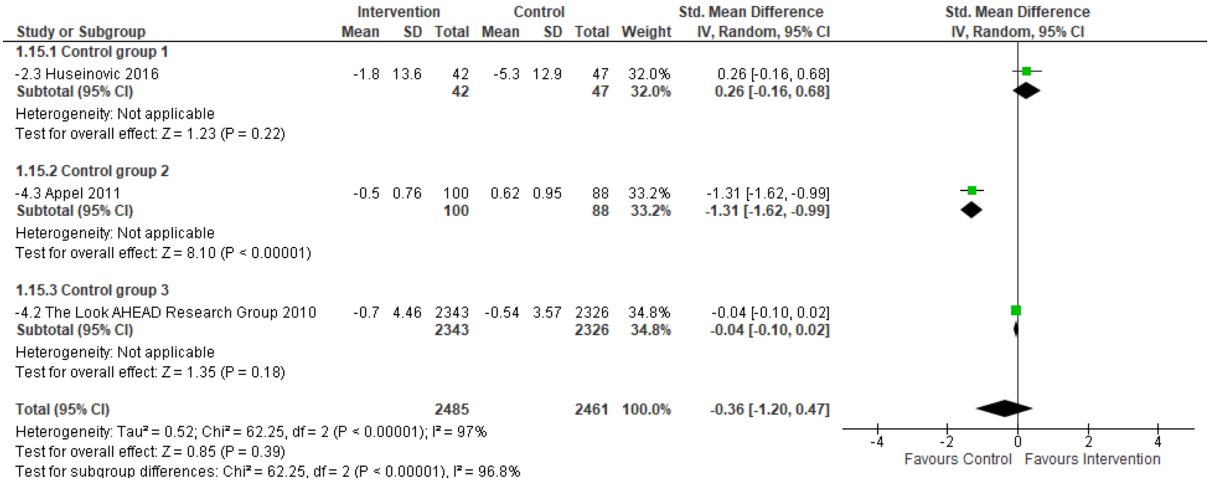

Figure S6f. Component Summaries at 31-36 months after programme end

Study or Subgroup	Intervention Mean	Intervention SD	Intervention Total	Control Mean	Control SD	Control Total	Mean Difference IV, Random, 95% CI
**1.15.1 Control group 3**							
-3.6 The Look AHEAD Research Group 2010 (1)	-0.36	2.84	2304	-0.25	2.54	2290	-0.11 [-0.27, 0.05]

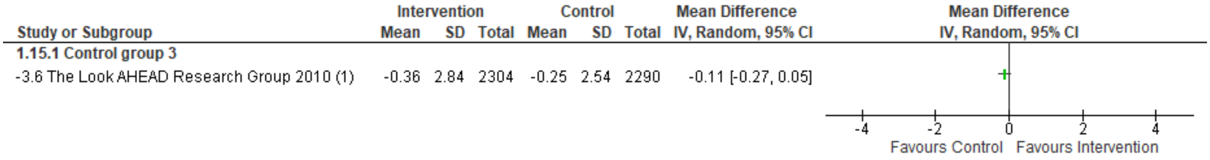

Footnotes
(1) Data from The Look AHEAD Research Group 2010 was estimated from a figure.

Figure S6g. Component Summaries at 49-54 months after programme end

Study or Subgroup	Intervention Mean	Intervention SD	Intervention Total	Control Mean	Control SD	Control Total	Mean Difference IV, Random, 95% CI
**1.16.1 Control group 3**							
-3.4 The Look AHEAD Research Group 2010 (1)	-0.12	4.27	2266	-0.16	3.29	2237	0.04 [-0.18, 0.26]

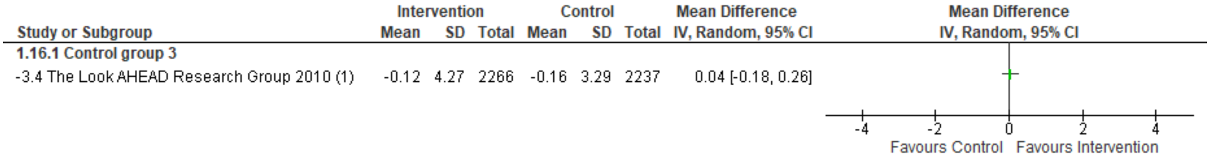

Footnotes
(1) Data from The Look AHEAD Research Group 2010 was estimated from a figure.

Figure S6h. Component Summaries at 55-60 months after programme end

Study or Subgroup	Intervention Mean	Intervention SD	Intervention Total	Control Mean	Control SD	Control Total	Mean Difference IV, Random, 95% CI
**1.16.1 Control group 3**							
-2.8 The Look AHEAD Research Group 2010 (1)	-0.03	4.31	2253	-0.23	3.35	2211	0.20 [-0.03, 0.43]

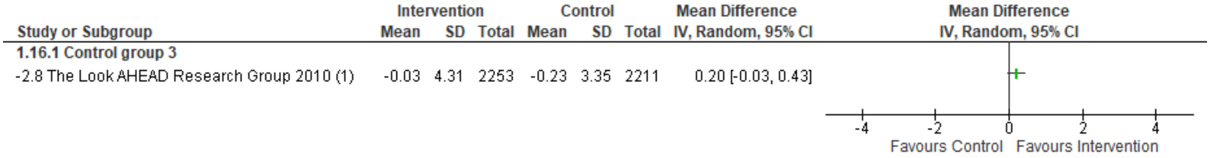

Footnotes
(1) Data from The Look AHEAD Research Group 2010 was estimated from a figure.

Figure S6i. Component Summaries at 67-72 months after programme end

Study or Subgroup	Intervention Mean	Intervention SD	Intervention Total	Control Mean	Control SD	Control Total	Mean Difference IV, Random, 95% CI
**1.16.1 Control group 3**							
-2.8 The Look AHEAD Research Group 2010 (1)	-0.09	2.74	2225	-0.13	2.34	2185	0.04 [-0.11, 0.19]

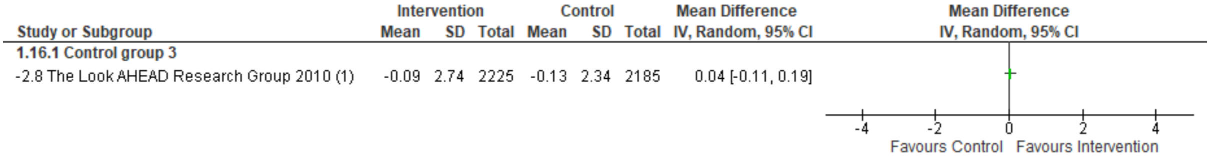

Footnotes
(1) Data from The Look AHEAD Research Group 2010 was estimated from a figure.

Figure S6j. Component Summaries at 79-84 months after programme end

Study or Subgroup	Intervention Mean	Intervention SD	Intervention Total	Control Mean	Control SD	Control Total	Mean Difference IV, Random, 95% CI
**1.17.1 Control group 3**							
-2.0 The Look AHEAD Research Group 2010 (1)	-0.22	4.44	2207	-0.47	3.57	2157	0.25 [0.01, 0.49]

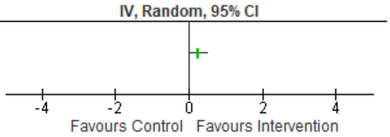

Footnotes
(1) Data from The Look AHEAD Research Group 2010 was estimated from a figure.

Figure S6k. Component Summaries at 91--96 months after programme end

Study or Subgroup	Intervention Mean	Intervention SD	Intervention Total	Control Mean	Control SD	Control Total	Mean Difference IV, Random, 95% CI
**1.18.1 Control group 3**							
The Look AHEAD Research Group 2010 (1)	0.01	4.21	1797	-0.4	3.19	1768	0.41 [0.17, 0.65]

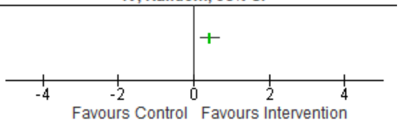

Footnotes
(1) Data from The Look AHEAD Research Group 2010 was estimated from a figure; Weight change data not available

Figure S6l. Component Summaries at 103--108 months after programme end

Study or Subgroup	Intervention Mean	Intervention SD	Intervention Total	Control Mean	Control SD	Control Total	Mean Difference IV, Random, 95% CI
**1.19.1 Control group 3**							
The Look AHEAD Research Group 2010 (1)	-0.19	3.91	961	-0.25	2.99	956	0.06 [-0.25, 0.37]

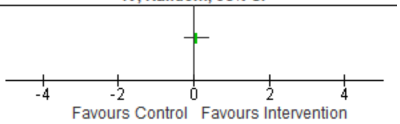

Footnotes
(1) Data from The Look AHEAD Research Group 2010 was estimated from a figure; Weight change data not available

Comparison 2 – BWMP (diet and exercise) versus diet only

Figure S6m. Component Summaries at programme end

Study or Subgroup	Diet and Exercise Mean	Diet and Exercise SD	Diet and Exercise Total	Diet only Mean	Diet only SD	Diet only Total	Weight	Std. Mean Difference IV, Random, 95% CI
-0.8 Messier 2013	-0.3	2.16	138	-0.9	2.37	123	59.9%	0.26 [0.02, 0.51]
-0.2 deRoon 2017	0.8	2.53	88	-0.1	2.3	88	40.1%	0.37 [0.07, 0.67]
**Total (95% CI)**			**226**			**211**	**100.0%**	**0.31 [0.12, 0.50]**

Heterogeneity: Tau² = 0.00; Chi² = 0.29, df = 1 (P = 0.59); I² = 0%
Test for overall effect: Z = 3.19 (P = 0.001)

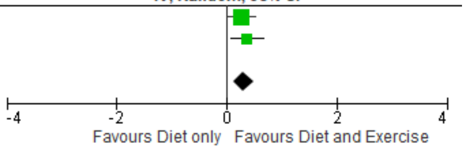

Figure S6n. Component Summaries at 1-6 months after programme end

Study or Subgroup	Diet and Exercise Mean	Diet and Exercise SD	Diet and Exercise Total	Diet only Mean	Diet only SD	Diet only Total	Mean Difference IV, Random, 95% CI
-1.8 Foster-Schubert 2012	3.2	3.53	108	3.4	3.69	105	-0.20 [-1.17, 0.77]

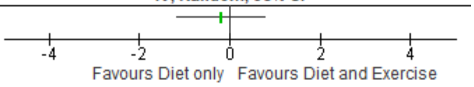

Figure S6o. Component Summaries at 7-12 months programme end

Study or Subgroup	Diet and Exercise Mean	Diet and Exercise SD	Diet and Exercise Total	Diet only Mean	Diet only SD	Diet only Total	Mean Difference IV, Random, 95% CI
-1.7 Messier 2013	-1.1	3.27	136	-1	4.01	129	-0.10 [-0.98, 0.78]

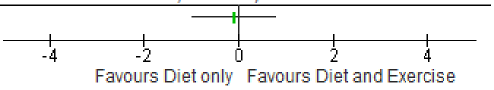

Comparison 3 – BWMP (diet and exercise) versus exercise only

Figure S6p. Component Summaries at programme end

Considerable heterogeneity unexplained by weight change differences observed.

Study or Subgroup	Diet and exercise Mean	SD	Total	Exercise only Mean	SD	Total	Weight	Std. Mean Difference IV, Random, 95% CI
-8.8 Messier 2013	-0.3	2.16	138	-0.4	2.06	130	50.5%	0.05 [-0.19, 0.29]
-2.5 Ard 2018 (1)	0.1	1.1	55	-2.2	1.1	54	49.5%	2.08 [1.61, 2.55]
**Total (95% CI)**			**193**			**184**	**100.0%**	**1.05 [-0.94, 3.04]**

Heterogeneity: Tau² = 2.02; Chi² = 57.05, df = 1 (P < 0.00001); I² = 98%
Test for overall effect: Z = 1.04 (P = 0.30)

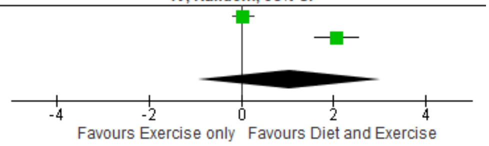

Footnotes
(1) Number of participants (N) at follow-up not available; imputed using N at baseline

Figure S6q. Component Summaries at 1-6 months after programme end

Study or Subgroup	Diet and Exercise Mean	SD	Total	Exercise only Mean	SD	Total	Mean Difference IV, Random, 95% CI
-2.6 Ard 2018	0	1.5	48	-0.3	1.2	45	0.30 [-0.25, 0.85]

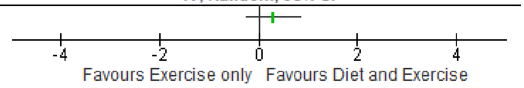

Figure S6r. Component Summaries at 7-12 months programme end

Study or Subgroup	Diet and Exercise Mean	SD	Total	Exercise only Mean	SD	Total	Mean Difference IV, Random, 95% CI
-8.8 Messier 2013	-1.1	3.27	136	-1.1	3.1	134	0.00 [-0.76, 0.76]

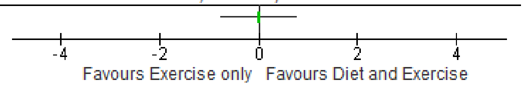

Comparison 4 – Intervention Vs Comparator intervention

Mental health composite scales head-to-head intervention comparisons

Most studies showed no difference in change in mental health component summary scores between intervention and comparator (95% CIs included crossed no difference) at programme end regardless of weight change differences between study groups (Figure S6s). One study favoured the comparator, but the 95% CIs incorporated the possibility of no difference (MD -0.42 [-0.85, 0.00]; n=87). By 1-6 months, one study favoured the intervention group which showed the greatest weight loss (MD 1.53 [1.07, 1.99]; n=94); no differences were found in the remaining two studies (Figure S6t) or the studies at 7-12 months and 13-18 months after programme end (Figures S6u-v).

Figure S6s. Component Summaries at programme end

Study or Subgroup	Intervention Mean	Intervention SD	Intervention Total	Comparator Mean	Comparator SD	Comparator Total	Std. Mean Difference IV, Random, 95% CI
-8.0 Messier 2013 (1)	-0.9	2.37	123	-0.4	2.06	130	-0.22 [-0.47, 0.02]
-2.4 Ard 2018 (2)	0.1	1.1	55	0.2	1.2	55	-0.09 [-0.46, 0.29]
-1.0 Schubel 2016 (3)	-3.2	3.8	44	3.4	21.6	43	-0.42 [-0.85, 0.00]
-0.9 van Wier 2011 (4)	0.04	12.04	257	0.02	9.58	256	0.00 [-0.17, 0.17]
-0.9 Ard 2004 (5)	0.9	9.22	236	-1	9.16	233	0.21 [0.02, 0.39]
+1.3 Burke 2015 (6)	2.14	5.11	48	2.48	3.92	59	-0.08 [-0.46, 0.31]

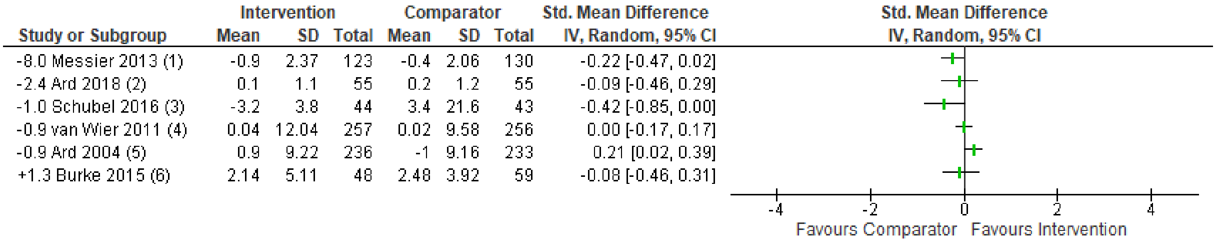

Footnotes
(1) Diet-induced weight loss only (D) Vs Exercise only (E)
(2) Diet plus Structured Aerobic Exercise Vs Diet and Lifestyle Activity; Number of participants (N) at follow-up not available; imputed using N at baseline
(3) Intermittent calorie restriction Vs Continuous Calorie Restriction
(4) Phone Group Vs Internet Group
(5) Established plus Dietary Approaches to Stop Hypertension (DASH) Vs Established
(6) Self-efficacy enhancement plus standard behavioural weight loss treatment (SBT) Vs SBT

Figure S6t. Component Summaries at 1-6 months after programme end

Study or Subgroup	Intervention Mean	Intervention SD	Intervention Total	Comparator Mean	Comparator SD	Comparator Total	Std. Mean Difference IV, Random, 95% CI
van Weir 2011 (1)	1.1	13.63	216	-0.63	10.66	182	0.14 [-0.06, 0.34]
-3.0 Ard 2018 (2)	0	1.5	48	-2.1	1.2	46	1.53 [1.07, 1.99]
+0.2 Burke 2015 (3)	1.99	5.14	47	2.96	4.27	57	-0.21 [-0.59, 0.18]

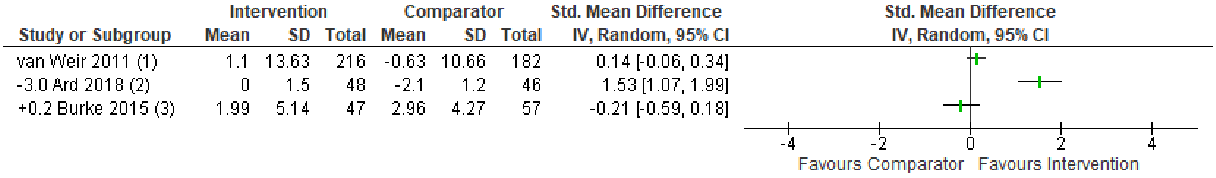

Footnotes
(1) Phone Group Vs Internet Group; No weight change data available
(2) Diet modification and energy restriction (Weight Loss) Vs Diet modification and body weight maintenance (Maintenance)
(3) Self-efficacy enhancement plus standard behavioural weight loss treatment (SBT) Vs SBT

Figure S6u. Component Summaries at 7-12 months after programme end

Study or Subgroup	Intervention Mean	Intervention SD	Intervention Total	Comparator Mean	Comparator SD	Comparator Total	Std. Mean Difference IV, Random, 95% CI
van Weir 2011 (1)	0.07	13.66	183	0.29	11.12	154	-0.02 [-0.23, 0.20]
-7.1 Messier 2013 (2)	-1	4.01	129	-1.1	3.1	134	0.03 [-0.21, 0.27]
-0.5 Ard 2004 (3)	0.2	9.37	244	-0.3	9.28	239	0.05 [-0.12, 0.23]
+0.2 Schubel 2016 (4)	3.4	28.6	43	3.3	15.9	40	0.00 [-0.43, 0.43]

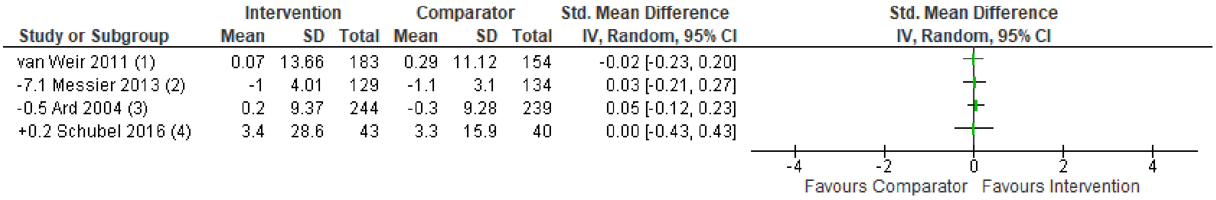

Footnotes
(1) Phone Group Vs Internet Group; No weight change data available
(2) Diet-induced weight loss only (D) Vs Exercise only (E)
(3) Established plus Dietary Approaches to Stop Hypertension (DASH) Vs Established
(4) Intermittent calorie restriction Vs Continuous Calorie Restriction

Figure S6v. Component Summaries at 13-18 months after programme end

Study or Subgroup	Intervention Mean	Intervention SD	Intervention Total	Comparator Mean	Comparator SD	Comparator Total	Std. Mean Difference IV, Random, 95% CI
+0.4 van Wier 2011 (1)	0.55	13.83	199	0.09	12.08	168	0.04 [-0.17, 0.24]

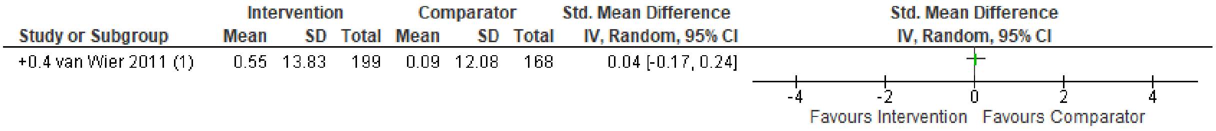

Footnotes
(1) Phone Group Vs Internet Group

Stress

Outcome measures:

1. Brief symptom inventory
2. Global Perceived Stress Scale (STS) score (Jackson Heart study)
3. Global Severity Index-90 (GSI-90) based on the German version of the Symptom Checklist (SCL-90R)
4. OQ-45.2 (Italian language version)
5. Perceived Stress Scale
6. The 10-item Perceived Stress Scale

Direction of mental health scale: higher score = greater perceived stress/psychological distress

Comparison 1 – BWMP (diet and/or exercise) versus Control group 1-4

Figure S7a. Stress at 1-6 months after programme end

	Intervention			Control				Std. Mean Difference
Study or Subgroup	Mean	SD	Total	Mean	SD	Total	Weight	IV, Random, 95% CI
**1.15.1 Control group 1**								
-8.2 Foster-Schubert 2012	-0.38	0.66	108	0.18	0.67	80	49.4%	-0.84 [-1.14, -0.54]
-4.4 Foley 2016	-0.03	4.31	145	-0.29	3.77	153	50.6%	0.06 [-0.16, 0.29]
**Subtotal (95% CI)**			**253**			**233**	**100.0%**	**-0.38 [-1.27, 0.50]**
Heterogeneity: $Tau^2 = 0.39$; $Chi^2 = 22.01$, df = 1 (P < 0.00001); $I^2 = 95\%$								
Test for overall effect: Z = 0.85 (P = 0.40)								
**Total (95% CI)**			**253**			**233**	**100.0%**	**-0.38 [-1.27, 0.50]**
Heterogeneity: $Tau^2 = 0.39$; $Chi^2 = 22.01$, df = 1 (P < 0.00001); $I^2 = 95\%$								
Test for overall effect: Z = 0.85 (P = 0.40)								
Test for subgroup differences: Not applicable								

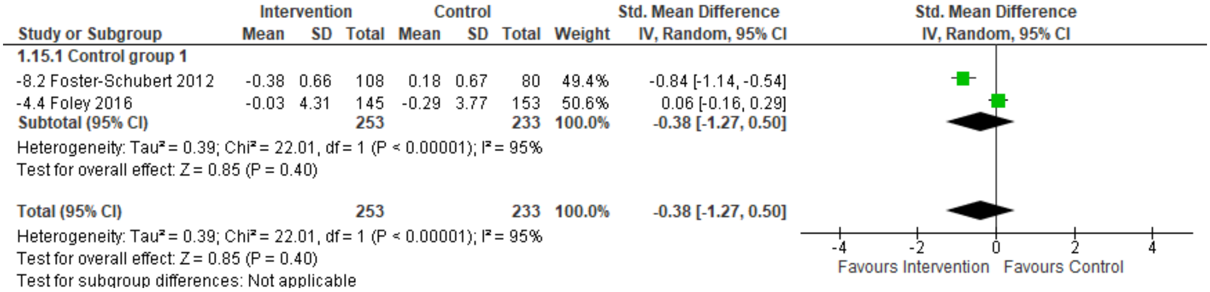

Figure S7b. Stress at 7-12 months after programme end

	Intervention			Control			Mean Difference
Study or Subgroup	Mean	SD	Total	Mean	SD	Total	IV, Random, 95% CI
**1.21.1 Control group 1**							
-3.9 Foley 2016	-0.51	3.97	152	-0.68	4.56	155	0.17 [-0.79, 1.13]

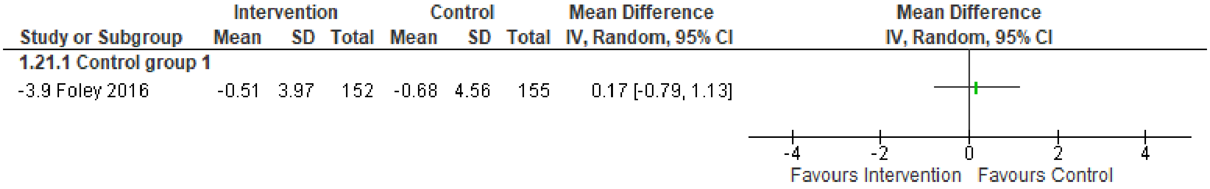

Comparison 2 – BWMP (diet and exercise) versus diet only

Figure S7c. Stress at 1-6 months after programme end

	Diet and Exercise			Diet Only			Mean Difference
Study or Subgroup	Mean	SD	Total	Mean	SD	Total	IV, Random, 95% CI
-1.8 Foster-Schubert 2012	-0.38	0.66	108	0.04	0.75	105	-0.42 [-0.61, -0.23]

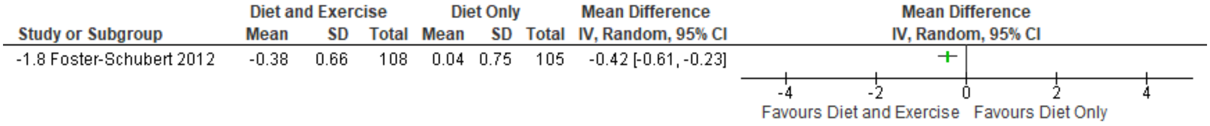

Comparison 3 – BWMPs (diet and exercise) versus exercise only (Control group 5)

No studies

Comparison 4 – Intervention Vs Comparator intervention

Stress head-to-head intervention comparisons

Five studies measured stress at programme end (Figure S7d). One study[9] favoured a mindfulness intervention in which greater weight loss was achieved versus an active control intervention (SMD -0.46 [-0.77, -0.14]; n=161). The direction of effect of three additional study arm comparisons[4, 10] also favoured the intervention, however 95% CIs included the possibility of favouring the comparator. One study[11] comparing psychodynamic versus behavioural treatment showed no difference between stress scores (SMD 0.00 [-0.29, 0.29]; n=161). The final study[12] favoured the *Brief strategic therapy* comparator arm over the *CBT* arm (SMD 2.42 [1.74, 3.10]; n=60), which may have been driven by the mean 8.5kg greater weight loss difference in this group. This study continued to favour this group which continued to show considerably greater mean weight loss differences at 1-6 months (SMD 3.58 [2.75, 4.42]; n=60) and 7-12 months (SMD 4.64 [3.64, 5.63]; n=60) after programme end (-10.0-13.1 kg mean weight loss difference in comparator arm; Figure S7f-S7g).

The direction of effect of all other comparisons at these time periods favoured the intervention with and without 95% CIs the included the possibility of favouring the comparator (Figure S7d-S7g).

Figure S7d. Stress at programme end

Study or Subgroup	Intervention Mean	Intervention SD	Intervention Total	Comparator Mean	Comparator SD	Comparator Total	Std. Mean Difference IV, Random, 95% CI
-1.2 Daubenmier 2016 (1)	-0.94	1.73	84	-0.22	1.36	77	-0.46 [-0.77, -0.14]
-0.8 Beutel 2006 (2)	-0.51	0.18	93	-0.51	0.17	93	0.00 [-0.29, 0.29]
+8.5 Jackson 2018 (3)	-14.47	6.22	30	-27.2	3.9	30	2.42 [1.74, 3.10]
+0.8 Katzer 2008b (4)	-0.13	0.3	48	-0.09	0.2	81	-0.16 [-0.52, 0.19]
+0.5 Ramirez 2001 (5)	-0.29	0.22	38	-0.27	0.18	27	-0.10 [-0.59, 0.40]
+0.1 Katzer 2008a (6)	-0.13	0.3	53	-0.09	0.2	81	-0.16 [-0.51, 0.18]

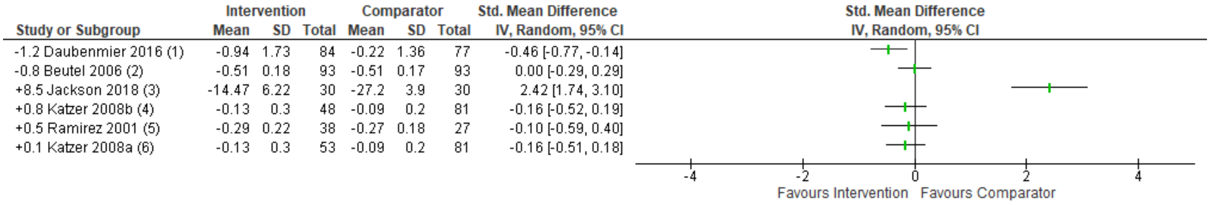

Footnotes
(1) Mindfulness intervention Vs Active control intervention
(2) Psychodynamic treatment Vs Behavioural treatment; Number of participants (N) imputed from N at nearest available timepoint
(3) Cognitive-Behavioral Therapy Vs Brief Strategic Therapy
(4) Group 'non-dieting' program (P2) Vs Mail-delivered 'non-dieting' program (P3); Same study as Katzer 2008a but different intervention group
(5) Weight control plus body image therapy Vs Weight control
(6) Group 'non-dieting' program based on Relaxation Response Training (P1) Vs Mail-delivered 'non-dieting' program (P3); Same study as Katzer 2008b but different intervention group

Figure S7f. Stress at 1 to 6 months after programme end

Study or Subgroup	Intervention Mean	Intervention SD	Intervention Total	Comparator Mean	Comparator SD	Comparator Total	Std. Mean Difference IV, Random, 95% CI
-2.1 Daubenmier 2016 (1)	0.01	3.07	79	0.23	2.54	70	-0.08 [-0.40, 0.24]
-0.2 Ramirez 2001 (2)	-0.25	0.24	38	-0.23	0.22	27	-0.09 [-0.58, 0.41]
+2.5 Katzer 2008b (3)	-0.16	0.4	39	-0.1	0.2	68	-0.21 [-0.60, 0.19]
+10.0 Jackson 2018 (4)	-10.84	5.69	30	-29.44	4.49	30	3.58 [2.75, 4.42]
+1.8 Katzer 2008a (5)	-0.16	0.3	41	-0.1	0.2	68	-0.25 [-0.63, 0.14]

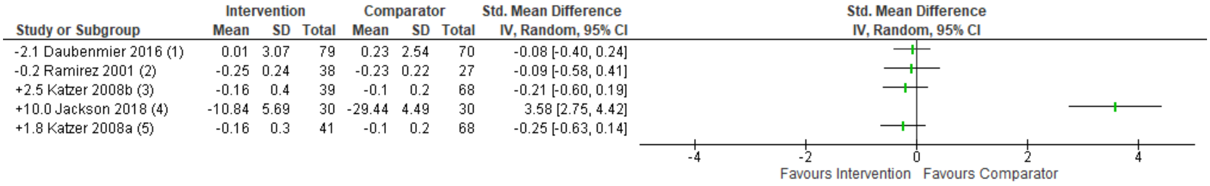

Footnotes
(1) Mindfulness intervention Vs Active control intervention
(2) Weight control plus body image therapy Vs Weight control
(3) Group 'non-dieting' program (P2) Vs Mail-delivered 'non-dieting' program (P3); Same study as Katzer 2008a but different intervention group
(4) Cognitive-Behavioral Therapy Vs Brief Strategic Therapy
(5) Group 'non-dieting' program based on Relaxation Response Training (P1) Vs Mail-delivered 'non-dieting' program (P3); Same study as Katzer 2008b but different intervention group

Figure S7g. Stress at 7 to 12 months after programme end

	Intervention			Comparator			Std. Mean Difference
Study or Subgroup	Mean	SD	Total	Mean	SD	Total	IV, Random, 95% CI
-2.3 Ramirez 2001 (1)	-0.18	0.24	38	-0.04	0.2	27	-0.62 [-1.12, -0.11]
-2.1 Daubenmier 2016 (2)	-0.81	2.97	81	0.14	2.17	71	-0.36 [-0.68, -0.04]
-1.3 Beutel 2006 (3)	-0.28	0.29	93	-0.21	0.26	93	-0.25 [-0.54, 0.04]
-0.6 Katzer 2008a (4)	-0.22	0.3	36	-0.09	0.2	63	-0.54 [-0.95, -0.12]
+13.1 Jackson 2018 (5)	-7.94	6.39	30	-32.77	3.88	30	4.64 [3.64, 5.63]
+1.5 Katzer 2008b (6)	-0.19	0.3	38	-0.09	0.2	63	-0.41 [-0.82, -0.00]

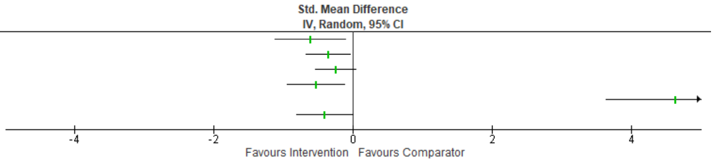

Footnotes
(1) Weight control plus body image therapy Vs Weight control
(2) Mindfulness intervention Vs Active control intervention
(3) Psychodynamic treatment Vs Behavioural treatment; Number of participants (N) at baseline used as N at follow-up was not available; Estimated 100kg baseline weight for each arm to calculate weight change difference.
(4) Group 'non-dieting' program based on Relaxation Response Training (P1) Vs Mail-delivered 'non-dieting' program (P3); Same study as Katzer 2008b but different intervention group
(5) Cognitive-Behavioral Therapy Vs Brief Strategic Therapy
(6) Group 'non-dieting' program (P2) Vs Mail-delivered 'non-dieting' program (P3); Same study as Katzer 2008a but different intervention group

Psychological wellbeing

Outcome measures:

1. Eight-item wellbeing questionnaire
2. Fragebogen zur Lebenszufriedenheit (FLZ)
3. Profile of Mood States Short Form scale of Total Mood Disturbance
4. Satisfaction with Life Scale (SWLS)
5. Short Form of the positive and negative affect scale (PANAS) – Positive Affect
6. Short Form of the positive and negative affect scale (PANAS) – Negative Affect

Direction of scale: higher score = better psychological wellbeing (measured through mood, satisfaction with life and 'psychological wellbeing' measured by quality of life, ability to cope, ease of decision making, personal value and happiness)

Comparison 1 – BWMP (diet and/or exercise) versus Control group 1-4

Figure S8a1. Psychological wellbeing at programme end

	Intervention			Control				Std. Mean Difference
**Study or Subgroup**	**Mean**	**SD**	**Total**	**Mean**	**SD**	**Total**	**Weight**	**IV, Random, 95% CI**
**1.15.1 Control group 1**								
-5.4 Hunt 2014 (1)	0.2	0.46	329	0.1	0.47	344	91.3%	0.21 [0.06, 0.37]
-0.5 Ash 2006 (2)	1.21	2.11	35	0.69	1.81	30	8.7%	0.26 [-0.23, 0.75]
**Subtotal (95% CI)**			**364**			**374**	**100.0%**	**0.22 [0.07, 0.36]**
Heterogeneity: Tau² = 0.00; Chi² = 0.03, df = 1 (P = 0.86); I² = 0%								
Test for overall effect: Z = 2.96 (P = 0.003)								
**Total (95% CI)**			**364**			**374**	**100.0%**	**0.22 [0.07, 0.36]**
Heterogeneity: Tau² = 0.00; Chi² = 0.03, df = 1 (P = 0.86); I² = 0%								
Test for overall effect: Z = 2.96 (P = 0.003)								
Test for subgroup differences: Not applicable								

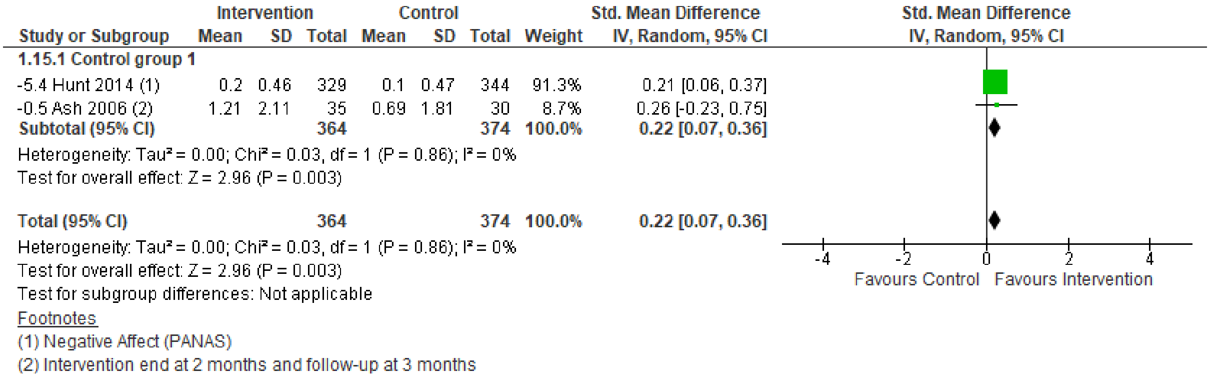

Footnotes
(1) Negative Affect (PANAS)
(2) Intervention end at 2 months and follow-up at 3 months

Figure S8a2. Psychological wellbeing at programme end

	Intervention			Control				Std. Mean Difference
**Study or Subgroup**	**Mean**	**SD**	**Total**	**Mean**	**SD**	**Total**	**Weight**	**IV, Random, 95% CI**
**1.16.1 Control group 1**								
-5.4 Hunt 2014 (1)	0.5	0.64	328	0.1	0.61	344	69.5%	0.64 [0.48, 0.79]
-0.5 Ash 2006 (2)	1.21	2.11	35	0.69	1.81	30	30.5%	0.26 [-0.23, 0.75]
**Subtotal (95% CI)**			**363**			**374**	**100.0%**	**0.52 [0.18, 0.87]**
Heterogeneity: Tau² = 0.04; Chi² = 2.10, df = 1 (P = 0.15); I² = 52%								
Test for overall effect: Z = 3.00 (P = 0.003)								
**Total (95% CI)**			**363**			**374**	**100.0%**	**0.52 [0.18, 0.87]**
Heterogeneity: Tau² = 0.04; Chi² = 2.10, df = 1 (P = 0.15); I² = 52%								
Test for overall effect: Z = 3.00 (P = 0.003)								
Test for subgroup differences: Not applicable								

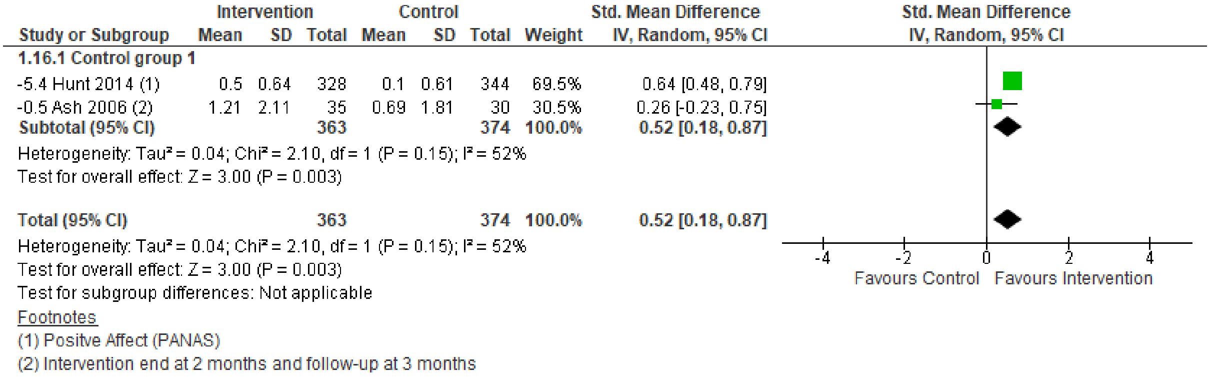

Footnotes
(1) Positve Affect (PANAS)
(2) Intervention end at 2 months and follow-up at 3 months

Figure S8b1. Psychological wellbeing at 7-12 months after programme end

Considerable heterogeneity unexplained by weight change differences observed.

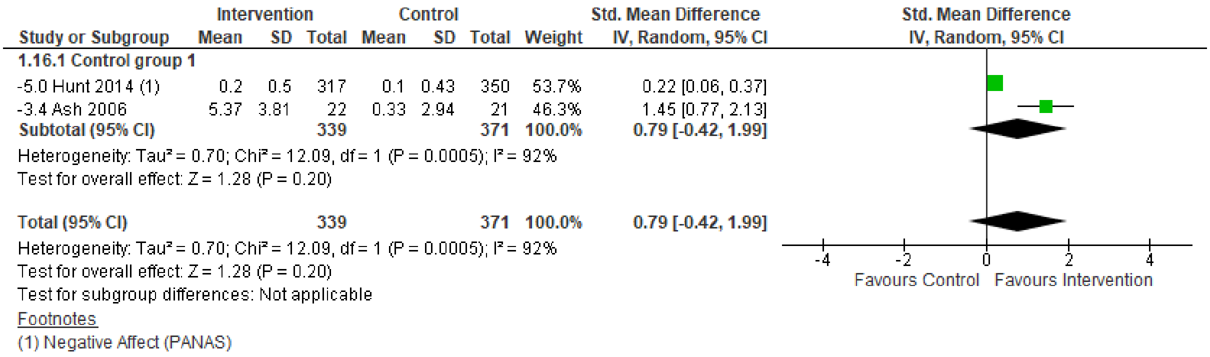

Figure S8b2. Psychological wellbeing at 7-12 months after programme end

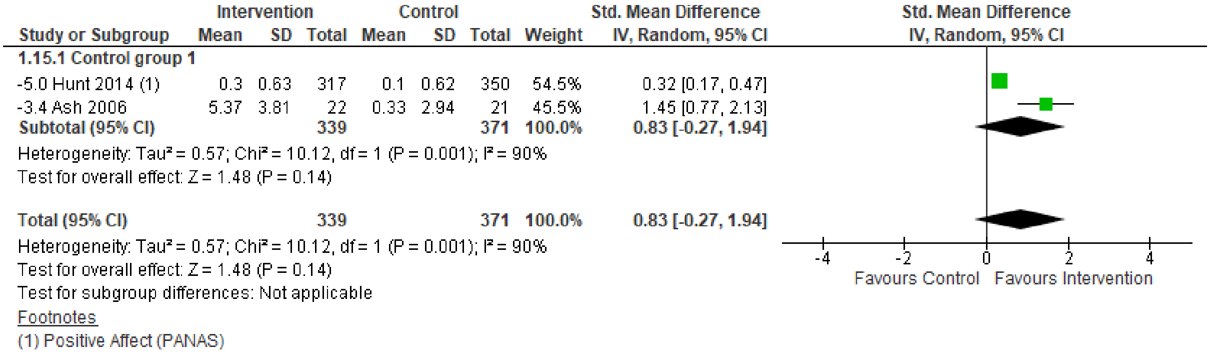

Comparison 2 – BWMP (diet and exercise) versus diet only

No studies

Comparison 3 – BWMP (diet and exercise) versus exercise only

No studies

Comparison 4 – Intervention Vs Comparator intervention

Psychological wellbeing head-to-head intervention comparisons

Two studies[13, 14] which showed greater weight loss in the more intensive study arm favoured this intervention at programme end, and at 1-6 months and 13-18 months after programme end, with 95% CIs excluding no difference (Figures S8c-d, S8f).

Another study[3] at programme end also favoured the *CBT* intervention over *behavioural weight loss treatment* comparator (SMD 0.91 [0.31, 1.50]; n=48). Two other studies[2, 7] favoured the comparator arms in which greater mean weight loss was achieved, however one study was at high risk of bias (Figure S8c).[7] By 7-12 months after programme end, the direction of effect of these two studies[2, 7] continued to favour the arm in which mean weight loss was greatest, however the possibility of no difference or of favouring the other arm could not be ruled out for one study[2] (Figure S8c).

Figure S8c. Wellbeing at programme end

Study or Subgroup	Intervention Mean	Intervention SD	Intervention Total	Comparator Mean	Comparator SD	Comparator Total	Std. Mean Difference IV, Random, 95% CI
-3.7 Annesi 2016 (1)	21.14	7.62	55	7.82	4.3	55	2.14 [1.67, 2.61]
-3.6 Annesi 2017 (2)	21.46	17.23	53	8	10.39	54	0.94 [0.54, 1.34]
+2.3 Munsch 2007 (3)	1.16	0.87	25	0.42	0.72	23	0.91 [0.31, 1.50]
+0.7 Ash 2006 (4)	1.21	2.11	35	2.23	2.48	51	-0.43 [-0.87, 0.00]
+0.4 Cleo 2018 (5)	66.8	82.9	22	93	106.6	17	-0.27 [-0.91, 0.36]

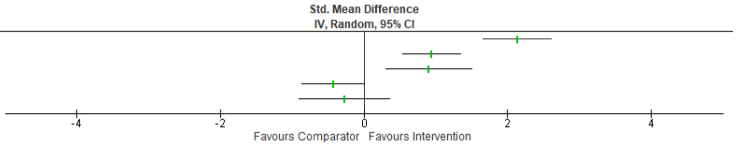

Footnotes
(1) The Coach Approach exercise-support protocol plus group nutrition sessions Vs Print manual plus telephone follow-ups
(2) Group social cognitive theory-based weight-management sessions emphasizing physical activity-derived self-regulation Vs manual and phone support; Number of participants at baseline used as N at follow-up was not available
(3) Cognitive-behavioural therapy Vs Behavioural weight loss treatment
(4) Fat Booters Incorporated Vs Individualised Dietetic Treatment; End of treatment at 2 months and follow-up at 3 months
(5) DSD Do Something Different Vs The Ten Top Tips

Figure S8d. Wellbeing at 1-6 months after programme end

Study or Subgroup	Intervention Mean	Intervention SD	Intervention Total	Comparator Mean	Comparator SD	Comparator Total	Std. Mean Difference IV, Fixed, 95% CI
-3.9 Annesi 2017 (1)	17.79	17.17	53	8.59	11.07	54	0.63 [0.24, 1.02]
-3.7 Annesi 2016 (2)	17.73	7.54	55	8.93	4.39	55	1.42 [1.00, 1.84]

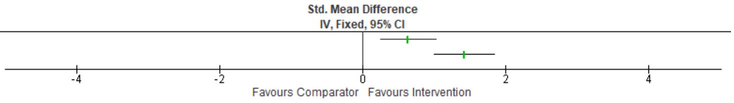

Footnotes
(1) Group social cognitive theory-based weight-management sessions emphasizing physical activity-derived self-regulation Vs manual and phone support; Number of participants at baseline used as N at follow-up was not available
(2) The Coach Approach exercise-support protocol plus group nutrition sessions Vs Print manual plus telephone follow-ups

Figure S8e. Wellbeing at 7-12 months after programme end

Study or Subgroup	Intervention Mean	Intervention SD	Intervention Total	Comparator Mean	Comparator SD	Comparator Total	Std. Mean Difference IV, Random, 95% CI
-3.6 Munsch 2007 (1)	2.87	1.97	22	0.79	1.28	20	1.22 [0.55, 1.88]
-1.1 Ash 2006 (2)	5.37	3.81	22	2.81	3.63	44	0.69 [0.16, 1.21]
+1.1 Cleo 2018 (3)	65.6	197.01	22	112.2	163.28	17	-0.25 [-0.88, 0.39]

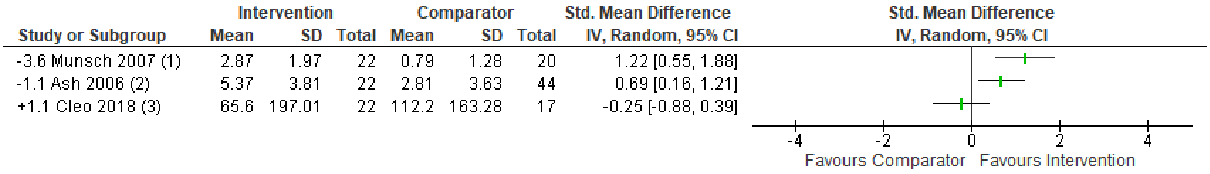

Footnotes
(1) Cognitive-behavioural therapy Vs Behavioural weight loss treatment
(2) Fat Booters Incorporated Vs Individualised Dietetic Treatment
(3) DSD Do Something Different Vs The Ten Top Tips

Figure S8f. Wellbeing at 13-18 months after programme end

Study or Subgroup	Intervention Mean	Intervention SD	Intervention Total	Comparator Mean	Comparator SD	Comparator Total	Std. Mean Difference IV, Random, 95% CI
-3.9 Annesi 2016 (1)	16.31	7.34	55	8.24	4.3	55	1.33 [0.92, 1.75]
-3.8 Annesi 2017 (2)	16.4	16.93	63	8.04	11.08	54	0.57 [0.20, 0.94]

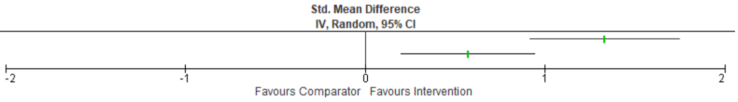

Footnotes
(1) The Coach Approach exercise-support protocol plus group nutrition sessions Vs Print manual plus telephone follow-ups
(2) Group social cognitive theory-based weight-management sessions emphasizing physical activity-derived self-regulation Vs manual and phone support; Number of participants at baseline used as N at follow-up was not available

Impact of weight on Quality of Life (QoL)

Outcome measures:

1. Impact of Weight on Quality of Life-Lite (IWQOL-Lite)

Direction of mental health scale: higher score = poorer weight related QoL

Findings

Two studies measured obesity-specific QoL using the Impact of Weight change on Quality of life-Lite questionnaire (IWQOL-Lite). For this analysis, higher scores indicate poorer obesity-specific QoL. No studies were at high risk of bias. A summary of all effect estimates is presented in Table 3 in the main paper.

BWMP versus Control

One study compared a BWMP intervention to a non/minimal control group 3.[15] This study showed a reduction in scores which indicate an improvement obesity-specific QoL which favoured the intervention arm at programme end (MD -3.20 [-5.24, -1.18]; n=162) and 1-6 months after programme end (MD –3.50 [-6.92, -0.08]; n=150).

Direct comparisons between BWMPs

No studies compared a diet and exercise intervention to a diet only comparator.

Diet and exercise intervention versus Exercise only

A single study showed mixed changes in IWQOL-Lite scores between a diet and exercise intervention and exercise only comparison group at and 1-6 after programme end. At programme end, a MD of -1.40 [-1.95, -0.85; n=109] favoured the intervention group, which also showed greater weight loss (Figure S9c). By 1-6 months, the exercise only arm was favoured (0.80 [0.19, 1.41]; n=93) despite a great weight loss remaining in the intervention group (Figure S7d).

Comparison 1 – BWMP (diet and/or exercise) versus Control group 1-4

Figure S9a. Impact of weight on QoL at programme end

	Intervention			Control			Mean Difference
Study or Subgroup	Mean	SD	Total	Mean	SD	Total	IV, Random, 95% CI
**1.13.1 Control group 3**							
+0.8 deZwaan 2017	-12.6	6.73	85	-9.4	6.52	77	-3.20 [-5.24, -1.16]

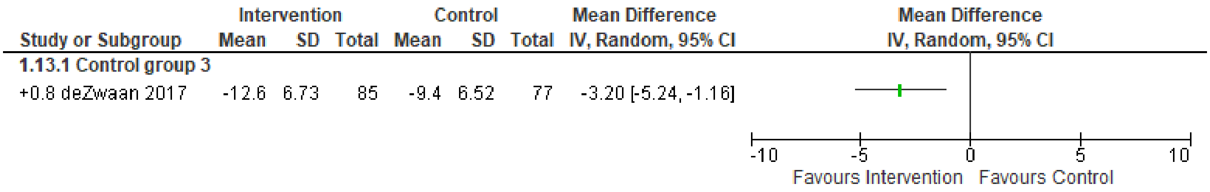

Figure S9b. Impact of weight on QoL after 1-6 months after programme end

Study or Subgroup	Intervention Mean	Intervention SD	Intervention Total	Control Mean	Control SD	Control Total	Mean Difference IV, Random, 95% CI
**1.14.1 Control group 3**							
-0.5 deZwaan 2017	-16.9	11.59	80	-13.4	9.79	70	-3.50 [-6.92, -0.08]

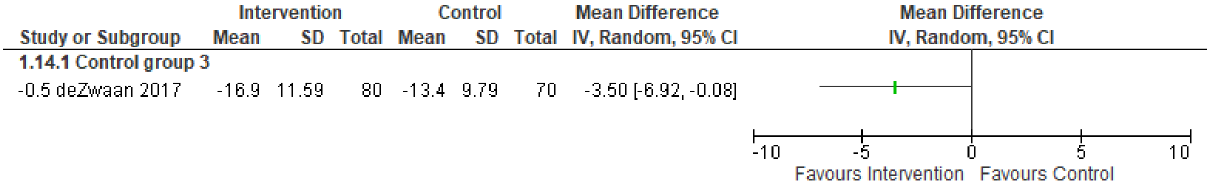

Comparison 2 – BWMP (diet and exercise) versus diet only

No studies

Comparison 3 – BWMP (diet and exercise) versus exercise only

Figure S9c. Impact of weight on QoL at programme end

Study or Subgroup	Diet and Exercise Mean	Diet and Exercise SD	Diet and Exercise Total	Exercise only Mean	Exercise only SD	Exercise only Total	Mean Difference IV, Random, 95% CI
-2.5 Ard 2018 (1)	-8.8	1.6	55	-7.4	1.3	54	-1.40 [-1.95, -0.85]

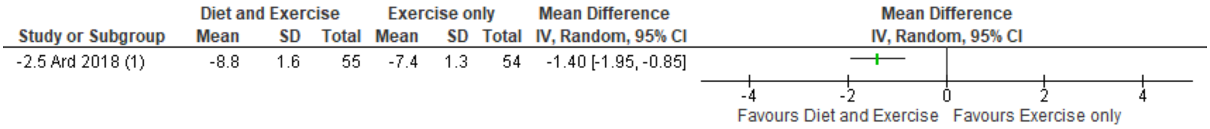

Footnotes
(1) Number of participants (N) at follow-up not available; imputed using N at baseline

Figure S9d. Impact of weight on QoL after 1-6 months after programme end

Study or Subgroup	Diet and Exercise Mean	Diet and Exercise SD	Diet and Exercise Total	Exercise only Mean	Exercise only SD	Exercise only Total	Mean Difference IV, Random, 95% CI
-2.6 Ard 2018	-11.1	1.5	48	-11.9	1.5	45	0.80 [0.19, 1.41]

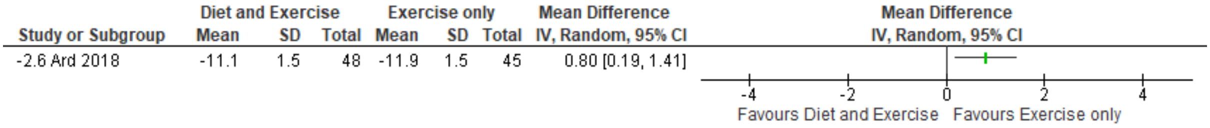

Comparison 4 – Intervention Vs Comparator intervention

Impact of weight on QoL head-to-head intervention comparisons

One study made a direct comparison between BWMPs.[16] This study found improved obesity-specific QoL between 1-6 months after an *exercise, diet quality and weight loss* intervention compared with an *exercise, diet quality and weight maintenance* intervention (-1.40 [-2.01, -0.79]; n=94; Figure S9f). No statistically significant difference was found at programme end (Figure S9e).

Figure S9e. Impact of weight on QoL at programme end

Study or Subgroup	Intervention Mean	Intervention SD	Intervention Total	Comparator Mean	Comparator SD	Comparator Total	Mean Difference IV, Random, 95% CI
-2.4 Ard 2018 (1)	-8.8	1.6	55	-8.6	1.6	55	-0.20 [-0.80, 0.40]

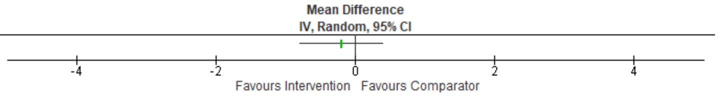

Footnotes
(1) Diet modification and energy restriction (Weight Loss) Vs Diet modification and body weight maintenance (Maintenance); Number of participants (N) at follow-up not available; imputed using N at baseline

Figure S9f. Impact of weight on QoL at 1-6 months after programme end

	Intervention			Comparator			Mean Difference
**Study or Subgroup**	**Mean**	**SD**	**Total**	**Mean**	**SD**	**Total**	**IV, Random, 95% CI**
-3.0 Ard 2018 (1)	-11.1	1.5	48	-9.7	1.5	46	-1.40 [-2.01, -0.79]

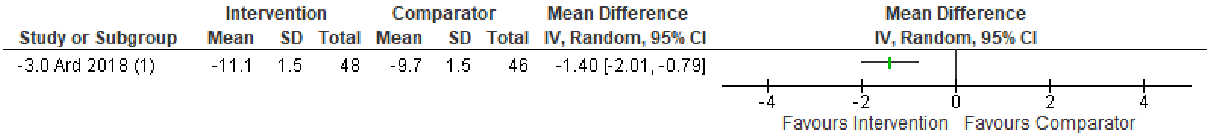

Footnotes
(1) Diet modification and energy restriction (Weight Loss) Vs Diet modification and body weight maintenance (Maintenance)

Body image

Outcome measures:

1. Body Image Avoidance Questionnaire (BIAQ)
2. Body Shape Questionnaire
3. Body Dysmorphic Disorder Examination - Self-Administration
4. Body Image Assessment questionnaire
5. Eating Disorder Examination Interview (EDE): Shape Concern
6. Physical Self-Perception Profile questionnaire - global physical self-worth scale
7. Physical Self-Perception Profile - body attractiveness scale
8. Multidimensional Body-Self Relations Questionnaire Appearance Scale (MBSRQ-AS)
9. Multidimensional Body-Self Relations Questionnaire Body Areas Satisfaction Scale (MBSRQ-BAS)

Direction of mental health scale: higher score = poorer mental health regarding body image

Findings

Five studies report nine different scales of body image. For our analysis, a higher score indicates poorer mental health regarding body image (e.g. greater body image-related dissatisfaction or concern).

BWMP versus Control

One study compared a BWMP intervention based on self-determination theory to control group 3, and reported changes in body image measured using four scales. [17] Sample size for each intervention arm was not reported so mean change and SD could not be calculated. For all scales, a greater reduction in mean change scores was reported for the intervention group, where mean weight loss was greatest.

Direct comparisons between BWMPs

No studies compared a diet and exercise intervention to a diet only comparator or an exercise only comparator.

Comparison 1 – BWMP (diet and/or exercise) versus Control group 1-4

No studies able to be pooled in meta-analysis

Comparison 2 – BWMP (diet and exercise) versus diet only

No studies

Comparison 3 – BWMP (diet and exercise) versus exercise only

No studies

Comparison 4 – Intervention Vs Comparator intervention

Body image head-to-head intervention comparisons

Four studies made other direct comparisons between BWMPs, some measuring body image using multiple scales. At programme end, one study reporting two body image scales showed improved mental health regarding body image in the intervention group which included body image therapy.[10] This statistically significant decrease in body image score persisted at 1-6 months and 7-12 months for one scale only. Another study found a reduced body image scores at the end of a non-diet wellness programme compared with a diet program, which persisted at 1-6 months after programme end.[18]

Another study showed reduced body image scores in a CBT versus behavioural weight loss intervention at programme end.[3] No other comparisons found differences at any other timepoints measured (Figure S10a-c).

Figure S10a. Body image at programme end

	Intervention			Comparator			Std. Mean Difference
Study or Subgroup	Mean	SD	Total	Mean	SD	Total	IV, Random, 95% CI
-5.1 Bacon 2002 (1)	-4.3	4.25	23	-8.2	3.14	36	1.07 [0.51, 1.63]
+2.3 Munsch 2007 (2)	-1.94	0.54	25	-1.56	0.4	23	-0.78 [-1.37, -0.19]
+0.7 Ames 2005b (3)	-4.4	1.71	13	-3.8	1.48	13	-0.36 [-1.14, 0.41]
+0.7 Ames 2005a (4)	-4.4	10.91	13	-1.9	4.3	13	-0.29 [-1.07, 0.48]
+0.5 Ramirez 2001b (5)	-40.3	8.13	38	-28.2	6.96	27	-1.56 [-2.12, -0.99]
+0.5 Ramirez 2001a (6)	-41.5	9.4	38	-32.7	7.05	27	-1.02 [-1.55, -0.50]

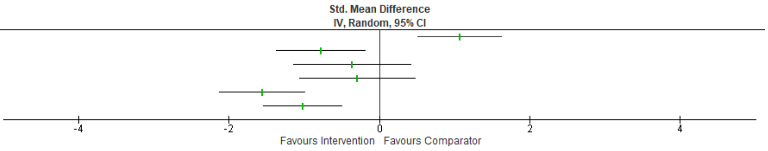

Footnotes
(1) Diet program Vs Non-diet wellness program
(2) Cognitive-behavioural therapy Vs Behavioural weight loss treatment
(3) Reformulated cognitive-behavioural treatment Vs Standard behavioural treatment; Same study as Ames 2005b but with different bidy image outcome measure; Number of participants (N) imputed from N at nearest available timepoint
(4) Reformulated cognitive-behavioural treatment Vs Standard behavioural treatment; Same study as Ames 2005a but with different body image outcome measure; Number of participants (N) imputed from N at nearest available timepoint
(5) Weight control plus body image therapy Vs Weight control; Same study as Ramirez 2001a but with different body image outcome measure
(6) Weight control plus body image therapy Vs Weight control; Same study as Ramirez 2001b but with different body image outcome measure

Figure S10b. Body image at 1-6 months after programme end

	Intervention			Comparator			Std. Mean Difference
Study or Subgroup	Mean	SD	Total	Mean	SD	Total	IV, Random, 95% CI
-6.2 Bacon 2002 (1)	-4.3	4.04	23	-6.9	3.77	29	0.66 [0.10, 1.22]
-0.2 Ramirez 2001b (2)	-38.3	12.2	38	-30.8	10.69	27	-0.64 [-1.14, -0.13]
-0.2 Ramirez 2001a (3)	-41.7	13.11	38	-36	10.08	27	-0.47 [-0.97, 0.03]
-0.2 Ames 2005b (4)	-2.1	2.06	13	-3.6	1.73	13	0.76 [-0.04, 1.56]
-0.2 Ames 2005a (5)	-4.4	5.83	13	-5.7	4.37	13	0.24 [-0.53, 1.02]

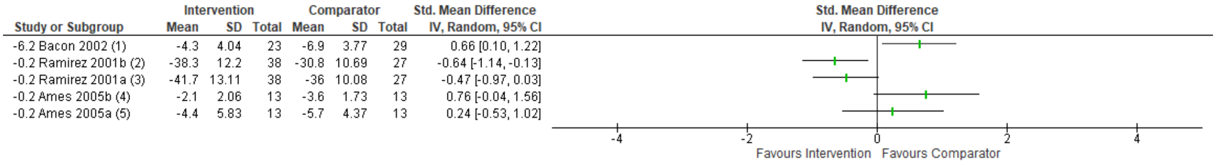

Footnotes
(1) Diet program Vs Non-diet wellness program
(2) Weight control plus body image therapy Vs Weight control; Same study as Ramirez 2001a but with different body image outcome measure
(3) Weight control plus body image therapy Vs Weight control; Same study as Ramirez 2001b but with different body image outcome measure
(4) Reformulated cognitive-behavioural treatment Vs Standard behavioural treatment; Same study as Ames 2005a but with different body image outcome measure
(5) Reformulated cognitive-behavioural treatment Vs Standard behavioural treatment; Same study as Ames 2005b but with different body image outcome measure

Figure S10c. Body image at 7-12 months after programme end

	Intervention			Comparator			Std. Mean Difference
Study or Subgroup	Mean	SD	Total	Mean	SD	Total	IV, Random, 95% CI
-3.6 Munsch 2007 (1)	-2.11	0.68	16	-1.97	0.49	18	-0.23 [-0.91, 0.44]
-2.3 Ramirez 2001b (2)	-31.7	14.14	38	-18.8	11.25	27	-0.98 [-1.50, -0.46]
-2.3 Ramirez 2001a (3)	-29.5	14.68	38	-24.2	13.54	27	-0.37 [-0.87, 0.13]

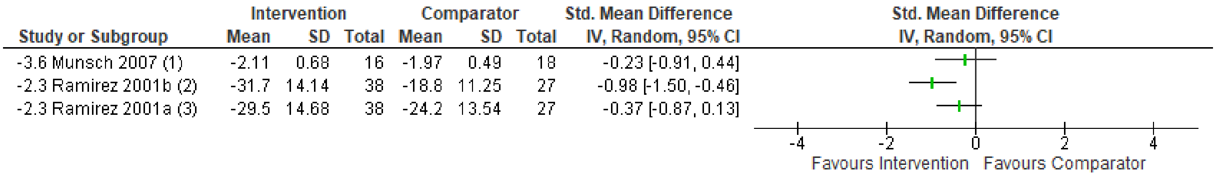

Footnotes
(1) Cognitive-behavioural therapy Vs Behavioural weight loss treatment
(2) Weight control plus body image therapy Vs Weight control; Same study as Ramirez 2001a but with different body image outcome measure
(3) Weight control plus body image therapy Vs Weight control; Same study as Ramirez 2001b but with different body image outcome measure

Eating Disorders

Outcome measures:

1. Binge Eating Scale
2. Eating Disorder Examination Interview (EDE) Global score
3. EDE: Dietary Restraint
4. EDE: Weight Concern
5. EDE: Eating Concern
6. EDE: Concern subscale

Direction of mental health scale: higher score = greater eating disorder symptomology or binge eating tendencies

Findings

Seven studies report on eating disorder measured using six different scales/subscales. For this analysis, higher scores indicate greater eating disorder symptomology or binge eating tendencies. A summary of all effect estimates is presented in Table 3.

BWMP versus Control

Two studies compared a BWMP intervention to a non/minimal control groups 1 and 3. At programme end, the direction of effect favoured the intervention group, suggesting a decrease in eating disorder scores which was particularly evident when a greater weight reduction was found in the intervention group, however statistical heterogeneity was moderate (SMD -0.42 [-0.85, 0.01]; I^2=44% n=207). No differences were found at 1-6 and 12 months after programme end, however the direction of effect favoured the intervention but 95% CI could not exclude the possibility of favouring the control (Figures S11a-S11c).

Direct comparisons between BWMPs

No studies compared a diet and exercise intervention to an exercise only comparator.

Diet and exercise intervention versus Diet only

One study showed a reduction in eating disorder scores which favoured the diet only comparator for all timepoint at and up to 18 months after programme end, with a greater weight loss in the intervention group; 95% CI showed no possibility of no difference or of favouring the intervention.[19] The study sample size was notably small, ranging from 65-76 across all time periods.

Comparison 1 – BWMP (diet and/or exercise) versus Control group 1-4

Figure S11a. Eating Disorders at programme end

Study or Subgroup	Intervention Mean	Intervention SD	Intervention Total	Control Mean	Control SD	Control Total	Weight	Std. Mean Difference IV, Random, 95% CI
**1.20.1 Control group 1**								
-2.5 Grilo 2014	-0.8	0.42	23	-0.5	0.39	22	33.7%	-0.73 [-1.33, -0.12]
**Subtotal (95% CI)**			**23**			**22**	**33.7%**	**-0.73 [-1.33, -0.12]**
Heterogeneity: Not applicable								
Test for overall effect: Z = 2.35 (P = 0.02)								
**1.20.2 Control group 3**								
+0.8 deZwaan 2017	-1	0.42	85	-0.9	0.33	77	66.3%	-0.26 [-0.57, 0.05]
**Subtotal (95% CI)**			**85**			**77**	**66.3%**	**-0.26 [-0.57, 0.05]**
Heterogeneity: Not applicable								
Test for overall effect: Z = 1.66 (P = 0.10)								
**Total (95% CI)**			**108**			**99**	**100.0%**	**-0.42 [-0.85, 0.01]**
Heterogeneity: Tau² = 0.05; Chi² = 1.79, df = 1 (P = 0.18); I² = 44%								
Test for overall effect: Z = 1.91 (P = 0.06)								
Test for subgroup differences: Chi² = 1.79, df = 1 (P = 0.18), I² = 44.3%								

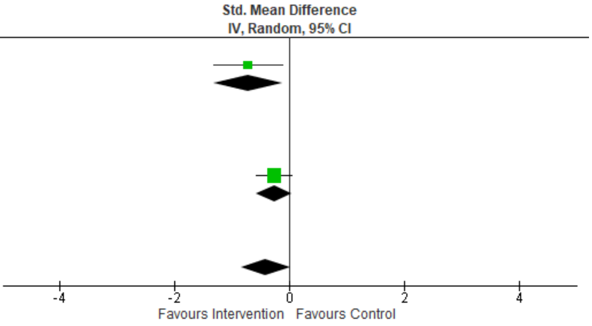

Figure S11b. Eating Disorders at 1-6 months after programme end

Study or Subgroup	Intervention Mean	Intervention SD	Intervention Total	Control Mean	Control SD	Control Total	Weight	Std. Mean Difference IV, Random, 95% CI
**1.16.1 Control group 1**								
-0.7 Grilo 2014	-0.8	0.44	23	-0.8	0.37	22	41.5%	0.00 [-0.58, 0.58]
**Subtotal (95% CI)**			**23**			**22**	**41.5%**	**0.00 [-0.58, 0.58]**
Heterogeneity: Not applicable								
Test for overall effect: Z = 0.00 (P = 1.00)								
**1.16.2 Control group 3**								
-0.5 deZwaan 2017	-1.2	0.53	80	-0.9	0.46	70	58.5%	-0.60 [-0.93, -0.27]
**Subtotal (95% CI)**			**80**			**70**	**58.5%**	**-0.60 [-0.93, -0.27]**
Heterogeneity: Not applicable								
Test for overall effect: Z = 3.58 (P = 0.0003)								
**Total (95% CI)**			**103**			**92**	**100.0%**	**-0.35 [-0.93, 0.23]**
Heterogeneity: Tau² = 0.12; Chi² = 3.06, df = 1 (P = 0.08); I² = 67%								
Test for overall effect: Z = 1.19 (P = 0.24)								
Test for subgroup differences: Chi² = 3.06, df = 1 (P = 0.08), I² = 67.4%								

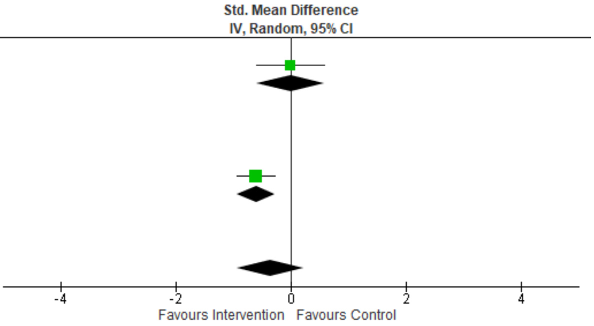

Figure S11c. Eating Disorders at 7-12 months after programme end

Study or Subgroup	Intervention Mean	Intervention SD	Intervention Total	Control group 1 Mean	Control group 1 SD	Control group 1 Total	Mean Difference IV, Random, 95% CI
-4.8 Grilo 2014	-0.9	0.48	21	-0.8	0.42	23	-0.10 [-0.37, 0.17]

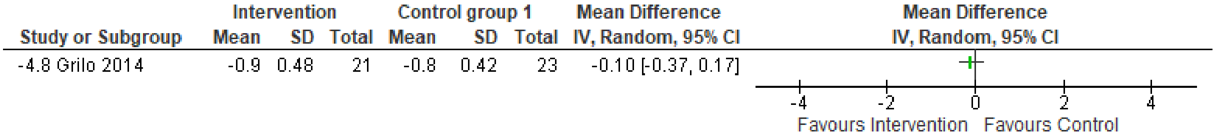

Comparison 2 – BWMP (diet and exercise) versus diet only

Figure S11d. Eating Disorders at programme end

Study or Subgroup	Diet and Exercise Mean	Diet and Exercise SD	Diet and Exercise Total	Diet only Mean	Diet only SD	Diet only Total	Mean Difference IV, Random, 95% CI
-9.1 Grilo 2011	-0.7	0.23	31	-0.9	0.22	34	0.20 [0.09, 0.31]

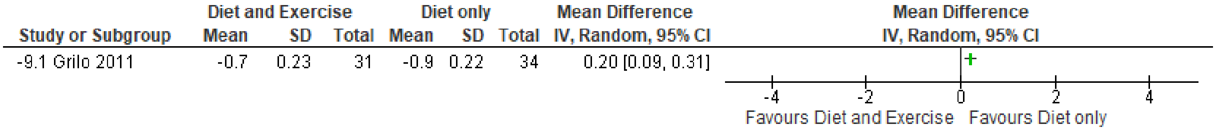

Figure S11e. Eating disorders at 1 to 6 months after

Study or Subgroup	Diet and Exercise Mean	Diet and Exercise SD	Diet and Exercise Total	Diet only Mean	Diet only SD	Diet only Total	Mean Difference IV, Random, 95% CI
-3.1 Grilo 2011	-0.7	0.38	39	-1	0.33	37	0.30 [0.14, 0.46]

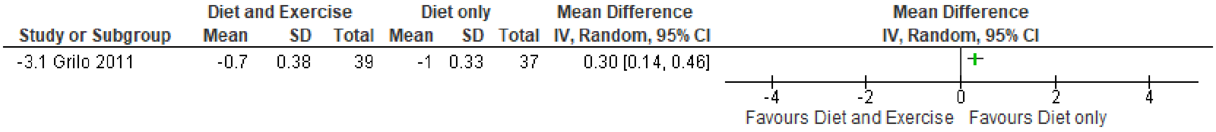

Figure S11f. Eating disorders at 13 to 18 months after

Study or Subgroup	Diet and Exercise Mean	SD	Total	Diet only Mean	SD	Total	Mean Difference IV, Random, 95% CI
-2.4 Grilo 2011	-0.9	0.39	37	-1.1	0.33	37	0.20 [0.04, 0.36]

Mean Difference
IV, Random, 95% CI

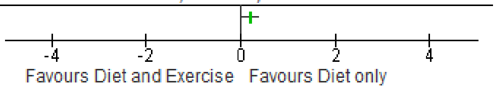

Comparison 3 – BWMP (diet and exercise) versus exercise only

No studies

Comparison 4 – Intervention Vs Comparator intervention

Eating disorders head-to-head intervention comparisons

Four studies reporting on seven eating disorder scales made other direct comparisons between BWMPs. At programme end, one study[10] showed reduced eating disorder symptomology scores in the intervention group that included body image therapy; this trend persisted at 1-6 months and 7-12 months after programme end. Reduced scores were also found in a CBT plus intensive versus minimal technological support intervention at programme end, and at 1-6 months after programme end. One study[8] showed reduced scores in a weight neutral versus weight loss intervention at programme end, however the opposite was found at 13-18 months after programme end. No other comparisons found differences at any other timepoints measured (Figure S11-g-h).

Figure S11g. Eating Disorders at programme end

Study or Subgroup	Intervention Mean	SD	Total	Comparator Mean	SD	Total	Std. Mean Difference IV, Random, 95% CI
-4.1 Mensinger 2016 (1)	-0.16	0.03	33	-0.83	0.03	37	22.09 [18.29, 25.88]
-0.3 Zwickert 2016 (2)	-10.1	2.19	26	-6.7	1.96	27	-1.61 [-2.24, -0.99]
+2.3 Munsch 2007 (3)	-1.5	0.46	25	-1.32	0.79	23	-0.28 [-0.85, 0.29]
+2.3 Munsch 2007 (4)	-1.23	0.39	25	-0.98	0.48	23	-0.56 [-1.14, 0.01]
+2.3 Munsch 2007 (5)	-1.65	0.48	25	-1.44	0.39	23	-0.47 [-1.04, 0.10]
+0.5 Ramirez 2001 (6)	-6.67	3.16	38	-3.82	1.87	27	-1.04 [-1.57, -0.51]
+0.5 Ramirez 2001 (7)	4.11	1.67	38	7.23	3.71	27	-1.14 [-1.67, -0.61]

Std. Mean Difference
IV, Random, 95% CI

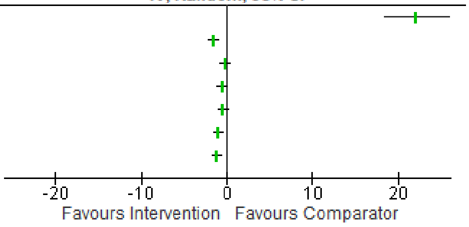

Footnotes
(1) Weight Loss Program Vs Weight-Neutral Program
(2) Cognitive-behaviour therapy plus intensive technological support Vs Cognitive-behaviour therapy plus minimal technological support
(3) Cognitive-behavioural therapy Vs Behavioural weight loss treatment; Same study as Munsch 2007 but with different eating disorder outcome...
(4) Cognitive-behavioural therapy Vs Behavioural weight loss treatment; Same study as Munsch 2007 but with different eating disorder outcome...
(5) Cognitive-behavioural therapy Vs Behavioural weight loss treatment; Same study as Munsch 2007 but with different eating disorder outcome...
(6) Weight control plus body image therapy Vs Weight control; Same study as Ramirez 2001 but with different eating disorder outcome measure
(7) Weight control plus body image therapy Vs Weight control; Same study as Ramirez 2001 but with different eating disorder outcome measure

Figure S11h. Eating Disorders at 1-6 months after programme end

Study or Subgroup	Intervention Mean	Intervention SD	Intervention Total	Comparator Mean	Comparator SD	Comparator Total	Std. Mean Difference IV, Random, 95% CI
-1.7 Zwickert 2016 (1)	-9.4	3.82	21	-5.8	2.99	24	-1.04 [-1.67, -0.41]
-0.2 Ramirez 2001 (2)	-6.09	3.15	38	-3.2	2.61	27	-0.97 [-1.49, -0.45]
-0.2 Ramirez 2001 (3)	-0.05	2.81	38	3.83	2.61	27	-1.40 [-1.96, -0.85]

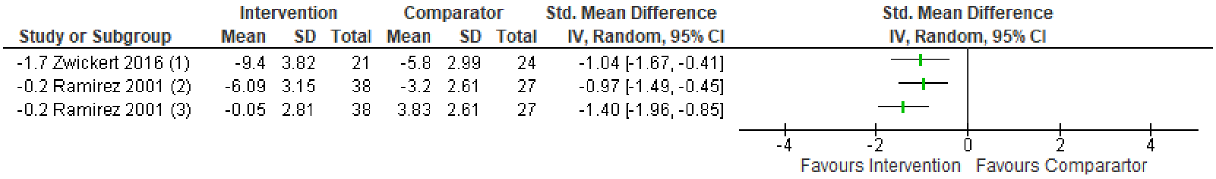

Footnotes
(1) Cognitive-behaviour therapy plus intensive technological support Vs Cognitive-behaviour therapy plus minimal technological support
(2) Weight control plus body image therapy Vs Weight control; Same study as Ramirez 2001 but with different eating disorder outcome measure
(3) Weight control plus body image therapy Vs Weight control; Same study as Ramirez 2001 but with different eating disorder outcome measure

Figure S11i. Eating Disorders at 7-12 months after programme end

Study or Subgroup	Intervention Mean	Intervention SD	Intervention Total	Comparator Mean	Comparator SD	Comparator Total	Std. Mean Difference IV, Random, 95% CI
-3.6 Munsch 2007 (1)	-1.96	0.65	16	-1.76	0.57	18	-0.32 [-1.00, 0.36]
-3.6 Munsch 2007 (2)	-1.62	0.8	16	-1.55	1.08	18	-0.07 [-0.74, 0.60]
-3.6 Munsch 2007 (3)	-1.06	0.63	16	-0.75	0.52	18	-0.53 [-1.21, 0.16]
-2.3 Ramirez 2001 (4)	-0.58	2.71	38	1.46	2.56	27	-0.76 [-1.27, -0.25]
-2.3 Ramirez 2001 (5)	-5.69	2.95	38	-1.65	2.56	27	-1.43 [-1.98, -0.87]
-2.0 Zwickert 2016 (6)	-5.5	3.74	15	-5	2.97	14	-0.14 [-0.87, 0.59]

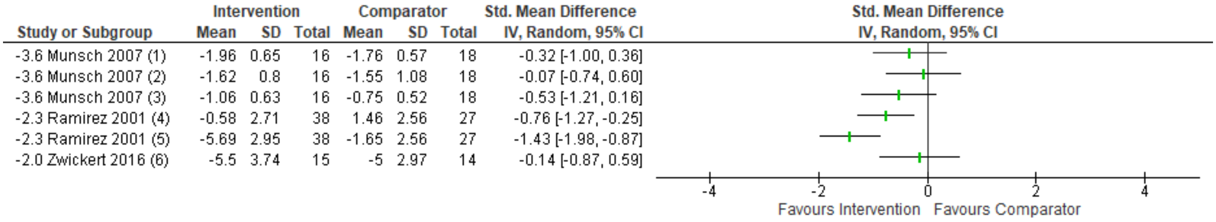

Footnotes
(1) Cognitive-behavioural therapy Vs Behavioural weight loss treatment; Same study as Munsch 2007 but with different eating disorder outcome measure
(2) Cognitive-behavioural therapy Vs Behavioural weight loss treatment; Same study as Munsch 2007 but with different eating disorder outcome measure
(3) Cognitive-behavioural therapy Vs Behavioural weight loss treatment; Same study as Munsch 2007 but with different eating disorder outcome measure
(4) Weight control plus body image therapy Vs Weight control; Same study as Ramirez 2001 but with different eating disorder outcome measure
(5) Weight control plus body image therapy Vs Weight control; Same study as Ramirez 2001 but with different eating disorder outcome measure
(6) Cognitive-behaviour therapy plus intensive technological support Vs Cognitive-behaviour therapy plus minimal technological support

Figure S11j. Eating Disorders at 13-18 months after programme end

Study or Subgroup	Intervention Mean	Intervention SD	Intervention Total	Comparator Mean	Comparator SD	Comparator Total	Mean Difference IV, Random, 95% CI
-2.9 Mensinger 2016 (1)	-0.25	0.07	21	1.9	2.89	19	-2.15 [-3.45, -0.85]

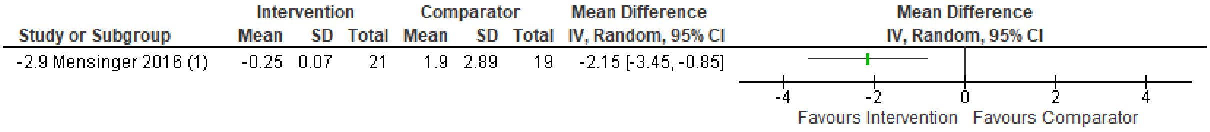

Footnotes
(1) Weight Loss Program Vs Weight-Neutral Program

DEVIATIONS FROM PROTOCOL

We had originally set out to test if any effect on mental health was modified by characteristics of the population but had insufficient data with which to do so. We had also planned to undertake step-wise meta-regression incorporating population and programme characteristics but were unable to do so due to insufficient data (≤10 studies) for the large majority of analyses. Instead, we conducted post-hoc analyses to model change in depression and/or anxiety over time and to test for associations between weight change and depression and/or anxiety, using data from studies reporting 'Depression and Anxiety', 'Depression', or 'Anxiety' as outcomes.

REFERENCES

1. Ames GE, Perri MG, Fox LD, et al. Changing weight-loss expectations: a randomized pilot study. *Eat Behav*. 2005;6(3):259-69. doi:10.1016/j.eatbeh.2005.01.003
2. Cleo G, Glasziou P, Beller E, Isenring E, Thomas R. Habit-based interventions for weight loss maintenance in adults with overweight and obesity: a randomized controlled trial. *Internat J Obes (Lond)*.2019;43(2):374-383. doi:10.1038/s41366-018-0067-4
3. Munsch S, Biedert E, Meyer A, et al. A randomized comparison of cognitive behavioral therapy and behavioral weight loss treatment for overweight individuals with binge eating disorder. *Int J Eat Disord*.2007;40(2):102-13. doi:10.1002/eat.20350
4. Katzer L, Bradshaw AJ, Horwath CC, Gray AR, O'Brien S, Joyce J. Evaluation of a "nondieting" stress reduction program for overweight women: a randomized trial. *Am J Health Promot*. 2008;22(4):264-74. doi:10.4278/060728113r1.1
5. Forman EM, Butryn ML, Manasse SM, et al. Acceptance-based versus standard behavioral treatment for obesity: Results from the mind your health randomized controlled trial. *Obesity (Silver Spring)*. 2016;24(10):2050-6. doi:10.1002/oby.21601
6. Wadden TA, Stunkard AJ. Controlled trial of very low calorie diet, behavior therapy, and their combination in the treatment of obesity. *J Consult Clin Psychol*. 1986;54(4):482-8. doi:10.1037//0022-006x.54.4.482
7. Ash S, Reeves M, Bauer J, et al. A randomised control trial comparing lifestyle groups, individual counselling and written information in the management of weight and health outcomes over 12 months. *Int J Obes (Lond)*. 2006;30(10):1557-64. doi:10.1038/sj.ijo.0803263
8. Mensinger JL, Calogero RM, Stranges S, Tylka TL. A weight-neutral versus weight-loss approach for health promotion in women with high BMI: A randomized-controlled trial. *Appetite*. 2016;105:364-74. doi:10.1016/j.appet.2016.06.006
9. Daubenmier J, Moran PJ, Kristeller J, et al. Effects of a mindfulness-based weight loss intervention in adults with obesity: A randomized clinical trial. *Obesity (Silver Spring)*. 2016;24(4):794-804. doi:10.1002/oby.21396
10. Ramirez EM, Rosen JC. A comparison of weight control and weight control plus body image therapy for obese men and women. *J Consult Clin Psychol*. 2001;69(3):440-6. doi:10.1037//0022-006x.69.3.440
11. Beutel ME, Dippel A, Szczepanski M, Thiede R, Wiltink J. Mid-term effectiveness of behavioral and psychodynamic inpatient treatments of severe obesity based on a randomized study. *Psychother Psychosom*. 2006;75(6):337-45. doi:10.1159/000095439
12. Jackson JB, Pietrabissa G, Rossi A, Manzoni GM, Castelnuovo G. Brief strategic therapy and cognitive behavioral therapy for women with binge eating disorder and comorbid obesity: A randomized clinical trial one-year follow-up. *J Consult Clin Psuchol*. 2018;86(8):688-701. doi:10.1037/ccp0000313
13. Annesi JJ. Mediation of the relationship of behavioural treatment type and changes in psychological predictors of healthy eating by body satisfaction changes in women with obesity. *Obes Res Clin Pract*. 2017;11(1):97-107. doi:10.1016/j.orcp.2016.03.011
14. Annesi JJ, Johnson PH, Tennant GA, Porter KJ, McEwen KL. Weight Loss and the Prevention of Weight Regain: Evaluation of a Treatment Model of Exercise Self-Regulation Generalizing to Controlled Eating. *Perm J*. 2016;20(3):15-146. doi:10.7812/tpp/15-146
15. de Zwaan M, Herpertz S, Zipfel S, et al. Effect of Internet-Based Guided Self-help vs Individual Face-to-Face Treatment on Full or Subsyndromal Binge Eating Disorder in Overweight or Obese Patients: The INTERBED Randomized Clinical Trial. *JAMA psychiatry*. 2017;74(10):987-995. doi:10.1001/jamapsychiatry.2017.2150

16. Ard JD, Gower B, Hunter G, et al. Effects of Calorie Restriction in Obese Older Adults: The CROSSROADS Randomized Controlled Trial. *J Gerontol A Biol Sci Med Sci*. 12 2017;73(1):73-80. doi:10.1093/gerona/glw237
17. Silva MN, Vieira PN, Coutinho SR, et al. Using self-determination theory to promote physical activity and weight control: a randomized controlled trial in women. *J Behav Med*. 2010;33(2):110-22. doi:10.1007/s10865-009-9239-y
18. Bacon L, Keim NL, Van Loan MD, et al. Evaluating a 'non-diet' wellness intervention for improvement of metabolic fitness, psychological well-being and eating and activity behaviors. *Int J Obes Relat Metab Disord*. 2002;26(6):854-65. doi:10.1038/sj.ijo.0802012
19. Grilo CM, Masheb RM, Wilson GT, Gueorguieva R, White MA. Cognitive-behavioral therapy, behavioral weight loss, and sequential treatment for obese patients with binge-eating disorder: a randomized controlled trial. *J Consult Clin Psychol*. 2011;79(5):675-85. doi:10.1037/a0025049
